# A Modular DNAzyme for Precise Visualization and Intervention of Alternative Splicing Isoforms in Live Cells

**DOI:** 10.1002/advs.202517895

**Published:** 2026-01-21

**Authors:** Mengru Lin, Jiale Sun, Yuqing Mao, Yuhao Tang, Fuan Wang, Zhihong Liu, Jing Wang

**Affiliations:** ^1^ College of Health Science and Engineering Hubei Province Key Laboratory of Biotechnology of Chinese Traditional Medicine Hubei University Wuhan Hubei P. R. China; ^2^ Department of Gastroenterology College of Chemistry and Molecular Sciences Zhongnan Hospital of Wuhan University Wuhan University Wuhan Hubei P. R. China

**Keywords:** alternative splicing, DNAzyme, live‐cell imaging, mRNA regulation

## Abstract

Alternative splicing is a fundamental mechanism that enhances proteomic diversity and modulates gene function, with its dysregulation being a hallmark of numerous diseases. Despite its biological significance, the real‐time monitoring of spliced mRNA isoforms in living cells remains challenging due to limited specificity and sensitivity in existing methods. Herein, we present a Stringent dUPlex‐activated Error‐Robust (SUPER) platform, an in situ, split‐DNAzyme‐based system enabling precise imaging of mRNA splicing events in live cells. SUPER employs an identical parental DNAzyme reassembled via isoform‐specific intron‐exon junctions, providing high‐fidelity discrimination of closely related splicing variants. Its dual‐site‐activated fluorescence design ensures error‐robust, background‐minimized imaging with spatial colocalization as an intrinsic validation mechanism. Beyond dynamic isoform profiling, the programmable nature of SUPER enables its conversion into a spatially confined catalytic antenna, locally activating therapeutic aptamers without affecting off‐target transcripts. This approach further allows for real‐time tracking of variant integrity and decay by monitoring subtle changes in probe colocalization. Our platform offers a powerful tool for dissecting splicing mechanisms and holds promise for therapeutic intervention in splicing‐associated diseases by enabling isoform‐selective gene regulation while mitigating oligonucleotide toxicity.

## Graphical abstract

A Stringent dUPlex‐activated Error Robust (SUPER) DNAzyme system enables real‐time imaging of alternative mRNA splicing (e.g., Bcl‐xL/Bcl‐xS) in living cells via target‐triggered split‐DNAzyme reassembly and dual‐color fluorescence. It also achieves mRNA‐selective knockdown through DNAzyme‐based gene regulation, serving as a versatile tool for splicing dynamics research and RNA‐guided precision medicine.

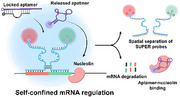

## 1 Introduction

Alternative mRNA splicing represents a critical mechanism of gene expression regulation that enables a single gene to produce multiple transcript isoforms by selectively including or excluding specific exons during pre‐mRNA processing [1, 2, 3, 4]. This finely tuned mechanism dramatically expands the functional and proteomic diversity of eukaryotic organisms, facilitating key biological processes such as tissue‐specific gene expression, developmental regulation, and rapid adaptation to environmental cues. Dysregulated splicing is increasingly recognized as a driver of pathogenesis in numerous diseases, including cancer [5, 6, 7], neurodegenerative disorders [8, 9], cardiovascular diseases [10, 11, 12], and immune dysfunction [13, 14], where aberrant isoform expression often leads to dysfunctional proteins, altered signaling pathways, and disrupted cellular homeostasis. While advances in next‐generation sequencing and transcriptomics have deepened the understanding of splicing landscapes at the population level [15, 16], much remains unknown about the spatial and temporal dynamics of splicing events within individual living cells. The ability to monitor alternative splicing in real time, within its native cellular context, is essential for elucidating the regulatory mechanisms governing isoform production, understanding how splicing is coordinated with transcriptional and translational processes, and uncovering how splicing is perturbed in diseases or modulated by therapeutic interventions.

To date, most existing technologies for splicing analysis rely on fixed‐cell approaches, limiting their ability to capture the dynamic nature of splicing regulation. Single‐molecule fluorescence in situ hybridization (smFISH) is widely utilized for visualizing distinct mRNA isoforms at subcellular resolution, providing valuable insights into isoform distribution and abundance [17]. However, smFISH requires multiple fluorescent probes for each isoform, necessitating complex probe design and increasing assay cost [18]. Furthermore, the requirement for chemical fixation restricts its application to static imaging, precluding the observation of transient or real‐time splicing dynamics in living cells. Other strategies, such as SpliceRCA—based on padlock probe‐mediated rolling circle amplification—have improved sensitivity and single‐cell spatial resolution by enabling specific targeting of exon‐exon junctions [19]. Nonetheless, the reliance on intracellular delivery of enzymes such as phi29 DNA polymerase and T4 ligase limits SpliceRCA to fixed‐cell systems, while increasing the risk of processing‐induced artifacts that could misrepresent isoform distribution and abundance.

To overcome these limitations, live‐cell‐compatible systems have been developed. For instance, plasmonic in situ dimerization using gold nanoparticles (AuNPs) enables the detection of spliced mRNA isoforms in living cells by exploiting nanoparticle proximity for signal generation [20]. However, this method is hindered by the tendency of AuNPs to aggregate and form nonspecific protein‐nanoparticle complexes in physiological environments, which reduces probe stability and introduces variability into signal output. Similarly, reduction‐triggered fluorescence (RETF) strategies based on Staudinger chemistry show promise for RNA imaging but suffer from low signal‐to‐noise ratios in the complex intracellular milieu [21, 22]. A common limitation of these live‐cell strategies is their dependence on proximity‐mediated activation, which increases the susceptibility to nonspecific interactions and false‐positive signals due to unintended probe aggregation or interactions with cellular macromolecules. In this context, deoxyribozymes (DNAzymes) represent a powerful alternative for intracellular biosensing. DNAzymes are synthetic single‐stranded DNA catalysts capable of performing target‐specific cleavage or chemical modification reactions [23, 24, 25]. Their programmable structure, robust catalytic activity, and capacity for autonomous signal amplification make them well‐suited for live‐cell applications [26, 27, 28] where high sensitivity, specificity, and low background noise are critical. Unlike the proximity‐based systems, DNAzymes can be engineered to remain catalytically inactive until precisely triggered by their target sequence, offering improved control over signal activation and minimizing nonspecific interference. Importantly, DNAzymes can be rationally engineered to remain catalytically inert until activated by their cognate target, enabling precise control over signal generation and minimizing nonspecific interference. Moreover, recent advances have demonstrated that engineered 8‐17‐family DNAzymes maintain target‐responsive activity and stability in vivo, including successful applications in small‐animal imaging [29] and tumor models [30], as well as clinical evaluation of DNAzyme therapeutics in human tissues [31], collectively underscoring their translational potential in complex biological environments.

Herein, we introduce a modular, DNAzyme‐based molecular circuit termed Stringent dUPlex‐activated Error‐Robust (SUPER). This live‐cell‐compatible system is designed to achieve the real‐time detection and spatial localization of alternative mRNA splicing events with high specificity and minimal background activation. Inspired by nature's multimeric nanomachines, which rely on the cooperative self‐assembly to perform complex tasks [32], we engineered a split DNAzyme system in which two inactive DNAzyme fragments are grafted onto target‐specific probes. Upon recognition of a specific mRNA splice junction, the probes hybridize to adjacent sites on the target transcript, triggering the reassembly of the catalytically active DNAzyme and enabling signal generation. The SUPER system integrates dual‐color, in situ “signal‐on” readouts, providing a built‐in ratiometric verification mechanism that significantly reduces the incidence of false positives through the co‐localization analysis. The system's performance was validated in live‐cell models, where it demonstrated strong concordance with RT‐qPCR, the current gold standard for isoform quantification. Moreover, SUPER enables the monitoring of mRNA decay dynamics in real time by reporting on the spatial separation of dual‐color probes during transcript degradation, as reflected by the reduced Pearson's correlation coefficients of the co‐localized fluorescence signals. This unique capability makes SUPER a valuable tool for probing not only the static presence but also the dynamic turnover of splicing isoforms within living cells. Beyond molecular imaging, the modularity of the SUPER platform can be leveraged for therapeutic applications. By serving as a molecular antenna, the reassembled DNAzyme can locally activate therapeutic oligonucleotides or small‐molecule effectors at sites of specific splicing events, offering new possibilities for targeted RNA regulation and minimizing off‐target toxicity often encountered in antisense or RNA interference‐based therapies. In summary, the SUPER platform presents a versatile and powerful solution for the real‐time visualization, quantification, and manipulation of alternative mRNA splicing isoforms in living cells. It holds broad potential for fundamental research in RNA biology, the discovery of novel therapeutic targets, and the development of RNA‐guided precision medicine strategies.

## 2 Results and Discussion

### 2.1 Design and Mechanism of the SUPER System for Discrimination of Bcl‐x Variants

**SCHEME 1:**
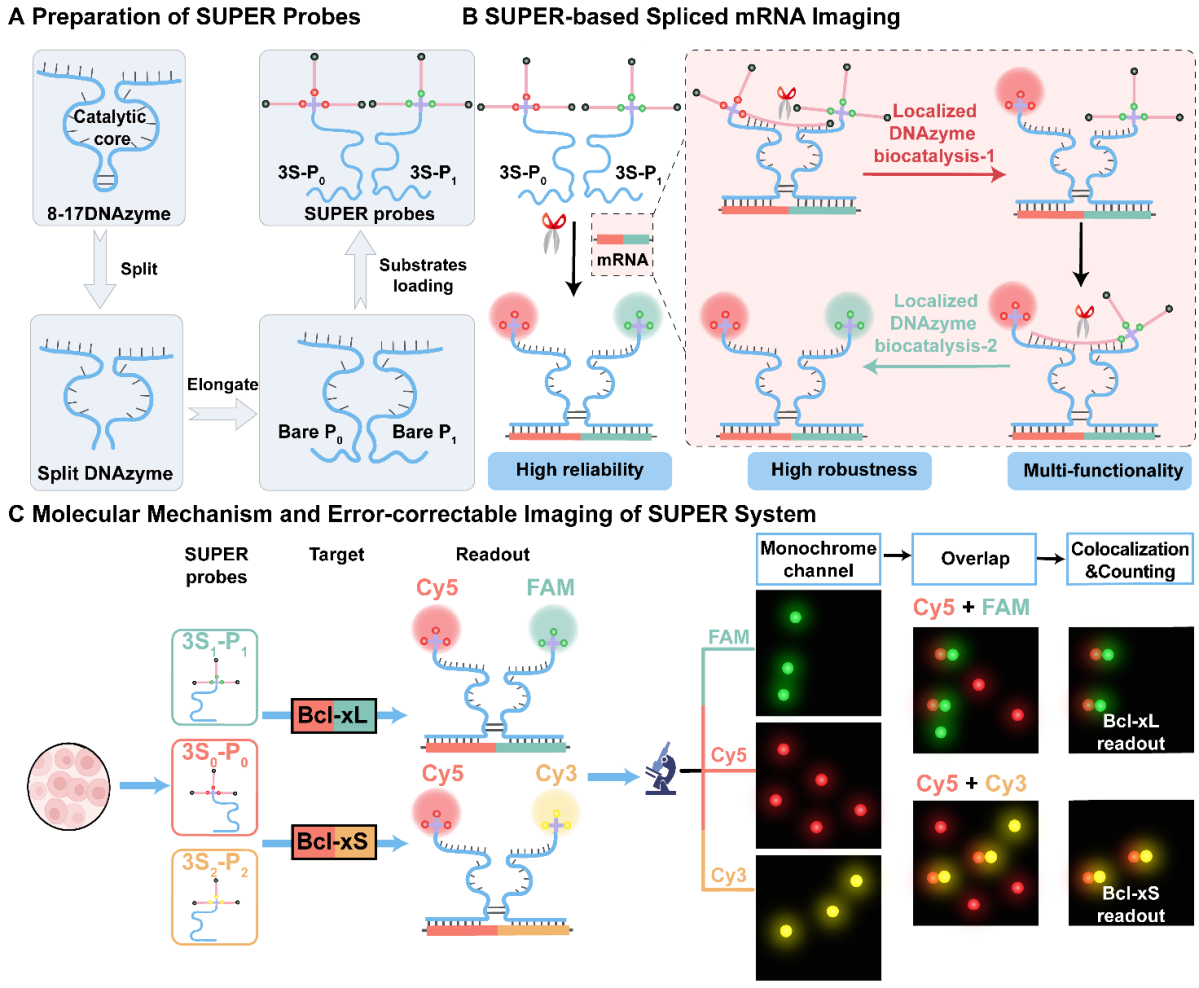
Design and operating principle of the SUPER system for spliced mRNA detection. (A) The preparation of SUPER probes. The 8–17 RNA‐cleaving DNAzyme is split into two inactive partzymes, each engineered to selectively recognize spliced mRNA fragments, and the biotin‐streptavidin coupling system is integrated into the SUPER probe design to enhance both detection sensitivity and robustness. (B) Schematic illustration of SUPER‐based spliced mRNA imaging. Upon target recognition, the partzymes reassemble into the active DNAzyme form and initiate cleavage of bilateral substrates, generating a dual‐color fluorescence signal and indicating the presence of target mRNA. (C) Working mechanism of the spliced mRNA‐activated SUPER system and error‐correctable imaging of the SUPER system. The specific target mRNA selectively recruited corresponding partzymes and triggered in situ dual‐color outputs. The co‐localized dual‐color signals are retained as authentic target signals, while independent signals are considered as false‐positive signals and subsequently eliminated.

The B‐cell lymphoma‐x (Bcl‐x) gene undergoes an alternative splicing process to generate two isoforms with distinct functions: the anti‐apoptotic Bcl‐xL (long isoform) and the pro‐apoptotic Bcl‐xS (short isoform) [33]. As illustrated in Figure S1, the two isoforms arise from differential splicing of exons 2 and 3, and the schematic also illustrates how the designed SUPER probes align with the splice junctions of Bcl‐xL and Bcl‐xS transcripts. The balance between these isoforms is critical for maintaining cellular homeostasis and is particularly important for cancer, where an elevated Bcl‐xL/Bcl‐xS ratio is closely associated with metastasis and chemoresistance [34, 35]. Discriminating these splice isoforms in live cells is essential for understanding the apoptotic regulation and its pathological implications, thereby offering valuable insights for therapeutic interventions. To achieve the precise, real‐time imaging of Bcl‐x spliced variants, we developed a splice junction‐anchored, in situ activated DNAzyme system for the simultaneous detection of Bcl‐xL and Bcl‐xS mRNA. As illustrated in Scheme 1A, we employed the RNA‐cleaving 8–17 DNAzyme, a widely adopted and well‐validated scaffold for split DNAzyme designs. In designing the split system, the 8–17 DNAzyme was divided at its catalytic core, following the canonical multicomponent nucleic acid enzyme (MNAzyme) strategy established by Mokany et al. [36] Splitting at the core rather than at peripheral arm regions ensures that the split fragments remain inert until they are reassembled into a functional DNAzyme upon the specific target recognition. The two sensor arms, complementary to these two splice mRNA fragments, were respectively grafted onto the partzymes to ensure high target specificity. For example, the bare partzyme (P) system designed for Bcl‐xL mRNA detection is composed of bare P_0_ and bare P_1_ probes. To enhance the detection robustness and sensitivity, the bare P_0_ and P_1_ probes were engineered via biotin‐streptavidin‐mediated conjugation to anchor different fluorophore/quencher pair‐labeled substrates (S_0_ and S_1_), thus generating the three‐substrate‐conjugated partzyme probes (referred to as 3S_0_‐P_0_ and 3S_1_‐P_1_). Upon recognition of Bcl‐xL mRNA, the 3S_0_‐P_0_ and 3S_1_‐P_1_ probes are recruited and refolded into the native DNAzyme conformation with high biocatalytic activity. This reactivation initiates the subsequent self‐confined successive cleavage of both substrates S_0_ and S_1_, thus resulting in the localized, dual‐color fluorescence readout signal (Scheme 1B). The underlying molecular reaction mechanism of the specific mRNA recognition and dual‐activated fluorescence generation is illustrated in Scheme 1C. To accurately discriminate between these different isoforms, Bcl‐xL and Bcl‐xS, we designed three SUPER‐based probes, 3S_0_‐P_0_, 3S_1_‐P_1,_ and 3S_2_‐P_2_, tailored for their respective targets. Since Bcl‐xL and Bcl‐xS isoforms share an identical exon 2 sequence [33], 3S_0_‐P_0_ serves as a common reference probe, while 3S_1_‐P_1_ and 3S_2_‐P_2_ are engineered to specifically recognize Bcl‐xL and Bcl‐xS, respectively. Bcl‐xL mRNA triggers the co‐localization of 3S_0_‐P_0_ and 3S_1_‐P_1_ for initiating DNAzyme‐amplified generation of Cy5 (cleavage of S_0_) and FAM (cleavage of S_1_) fluorescence, while Bcl‐xS mRNA triggers the co‐localization of 3S_0_‐P_0_ and 3S_2_‐P_2_ for initiating DNAzyme‐amplified co‐activation of Cy5 (cleavage of S_0_) and Cy3 (cleavage of S_2_) fluorescence. Here, the true expression of Bcl‐xL mRNA is indicated by the co‐localization of FAM and Cy5 fluorescence signals, while the co‐localized fluorescence signals of Cy3 and Cy5 confirm the true expression of Bcl‐xS mRNA. Note that all of these non‐co‐localized fluorescence signals are classified as false‐positive readouts, thereby reducing the nonspecific signal leakage. This in situ self‐confined, dual‐activated DNAzyme biocatalysis acts as a stringent molecular‐recognition‐to‐signal‐transduction “double‐check” mechanism, ensuring that only authentic splicing isoforms can generate true readout signals. Driven by the precisely reconstituted DNAzyme topology, the spatially self‐confined DNAzyme‐catalyzed signal transduction further guarantees the high specificity and accuracy for detecting target mRNAs. By combining the high‐resolution biomolecular recognition with error‐robust DNAzyme catalysis, this dual‐color imaging strategy enables simultaneous visualization of specific mRNA isoforms with low ambiguity, thus offering a powerful tool for the unbiased, multiplexed profiling of mRNA splicing variants. This approach holds significant potential for investigating these splicing dynamics, improving cancer diagnostics, and advancing splice‐switching therapeutic strategies.

### 2.2 In Vitro SUPER‐Mediated Responsive Sensing of Bcl‐xL and Bcl‐xS mRNA

**FIGURE 1:**
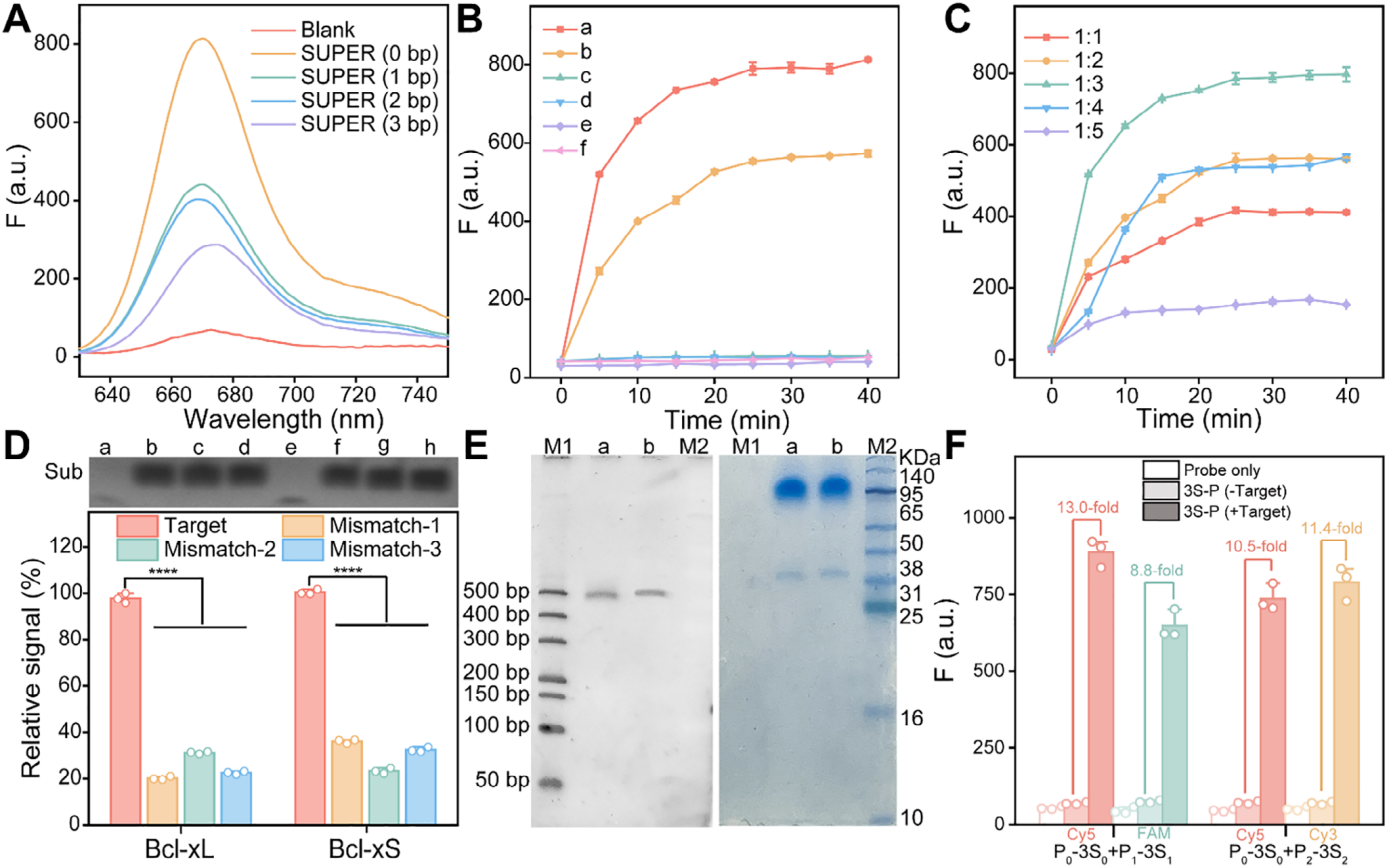
Bare P system‐mediated detection. (A) Fluorescence spectra of the different combinations of bare P_0_ and P_1_ for analyzing target Bcl‐xL mRNA. Blank represents the group containing only substrate (Sub), without DNAzyme and Bcl‐xL mRNA. All other groups contain the corresponding SUPER probes, Bcl‐xL mRNA, and Sub. (B) Kinetic analysis showing fluorescence generation by partzyme pairs requires both target mRNA and Mn^2+^ cofactor. a: P_0_+P_1_+Sub+Bcl‐xL+Mn^2+^, b: P_0_+P_2_+Sub+Bcl‐xS+Mn^2+^, c: P_0_+P_1_+Sub+Mn^2+^, d: P_0_+P_2_+Sub+Mn^2+^, e: P_0_+P_1_+Sub+Bcl‐xL, f: P_0_+P_2_+Sub+Bcl‐xS. (C) Real‐time fluorescence of bare P system at different ratios of DNAzyme‐to‐substrate upon Bcl‐xL mRNA under identical conditions. (D) Specificity of the bare P system for detecting target mRNA against a series of mismatched mRNA sequences. (Top) denaturing polyacrylamide gel electrophoresis (dPAGE) analysis of substrate cleavage. Lanes a, e: target mRNA (Bcl‐xL, Bcl‐xS); Lanes b–d, f–h: corresponding mismatched sequences. (Bottom) Fluorescence quantification of specificity. Relative signal was estimated from (F_m−_F_0_)/ (F_t−_F_0_) × 100%, where F_m_, F_0_, and F_t_ are the fluorescence peak of corresponding mismatched mRNA, blank and corresponding target mRNA, respectively. The relative signal of the target mRNA was defined as 100%. (E) Gel images showing the separation between one substrate conjugated‐P (1S‐P) and excess streptavidin. M1: 50 bp‐500 bp DNA ladder, a: biotin‐modified P_0_ and excess streptavidin, b: biotin‐modified P_1_ and excess streptavidin, M2: 10 KDa‐140 KDa protein marker. (F) Fluorescence intensities of the SUPER probes (3S_0_‐P_0_, 3S_1_‐P_1_, 3S_2_‐P_2_) with or without target mRNA, showing strong fluorescence activation only in the presence of Bcl‐xL or Bcl‐xS mRNA. Data are presented as mean ± SD (*n* = 3 independent experiments). p‐values were determined by a two‐tailed unpaired t‐test. ns: no significance, ^*^ *p* < 0.05, ^**^ *p* < 0.01, ^***^ *p* < 0.001, ^****^ *p* < 0.0001.

To evaluate the feasibility of the SUPER system, we first assessed the performance of the bare non‐self‐confined P system for detecting Bcl‐xL and Bcl‐xS mRNA. Here, the DNAzyme‐catalyzed signal transduction was monitored by using the non‐tethered free substrate that was labeled with a Cy5 fluorophore at the 5′ end and a BHQ2 quencher at the 3′ end. We systematically explored and optimized the catalytic efficiency of the partzyme system by designing a series of bare P_0_ and P_1_ variants with an extended complementary sequence at the DNAzyme catalytic core (0–3 bp, details in Table S1 and Figure S2). Fluorescence assays showed that the combination of P_0_ and P_1_ without any extension sequence (0 bp) exhibited the highest catalytic activity (Figure 1A), which might be attributed to the fact that the addition of the extension sequence to the binding domain potentially disrupts the ideal folding of the catalytic core. Consequently, these optimized partzymes were selected for further studies. The mRNA‐specific assembly of the DNAzyme was then investigated by fluorescence experiments (Figure 1B). No fluorescence enhancement occurred in the absence of Bcl‐xL or Bcl‐xS mRNA (curves c and d, respectively), confirming the high robustness of our system as indicated by the non‐activated DNAzyme. However, upon addition of the corresponding target mRNA, the fluorescence intensity increased significantly (∼19.8‐fold for Bcl‐xL and ∼15.7‐fold for Bcl‐xS), indicating the successful target‐induced DNAzyme reassembly and activation (curves a and b, respectively). Moreover, the absence of Mn^2+^ cofactors resulted in no fluorescence increase, indicating their essential role in DNAzyme biocatalysis (curves e and f, respectively). Gel electrophoresis further corroborated these findings, where the substrate band disappeared in the presence of Bcl‐xL or Bcl‐xS mRNA, confirming the efficient substrate cleavage by the activated DNAzyme (Figure S3). We next investigated the effect of varying DNAzyme‐to‐substrate ratios on the catalytic efficiency of DNAzyme (Figure 1C). A rapid fluorescence enhancement was observed upon mRNA‐mediated DNAzyme reassembly, reaching a plateau at approximately 30 min. The maximum activity was achieved at a 1:3 DNAzyme‐to‐substrate ratio, and this ratio was selected for subsequent experiments. The sequence specificity of the SUPER system was first examined using denaturing PAGE (dPAGE, Figure 1D, top). Specificity tests were conducted using three mutant mRNAs, each containing three mismatched bases at different positions (Table S1). The perfectly matched Bcl‐xL and Bcl‐xS targets produced clear substrate‐cleavage bands (lanes a and e), indicating successful reconstitution of the split DNAzyme. In contrast, all three mismatched variants (lanes b–d for Bcl‐xL and lanes f–h for Bcl‐xS) showed no detectable cleavage fragments, demonstrating that even small sequence deviations prevent catalytic activation. Fluorescence measurements yielded consistent results (Figure 1D, bottom). Fully complementary targets generated strong fluorescence signals, whereas the three mismatched variants exhibited substantially reduced signal intensities. Together, these results confirm that the SUPER system possesses high sequence specificity and can effectively discriminate closely related splice variants. Finally, the limit of detection (LOD) was calculated by using the 3σ method and was determined to be 10.3 pm for Bcl‐xL mRNA and 13.3 pm for Bcl‐xS mRNA (Figure S4). These results attest to the high sensitivity of the detection system, which arises from the cyclic cleavage capability of the DNAzyme.

Considering that the off‐target hybridization or undesired substrate hydrolysis can yield false‐positive signals, and that stochastic cleavage and uneven distribution of fluorescent substrate fragments may compromise cellular imaging, we developed the one substrate‐conjugated P (1S‐P) system. In this design, the split partzymes were respectively integrated with a single DNAzyme substrate, and produced the constructs denoted as 1S_0_‐P_0_, 1S_1_‐P_1_, and 1S_2_‐P_2_. This strategy was implemented to ensure the co‐localized, dual‐color imaging with minimal background interference. To evaluate the dual‐arm cleavage behavior of the 1S‐P system, we performed a dPAGE analysis using FAM‐labeled 1S_0_‐P_0_ and 1S_1_‐P_1_ (Figure S5). In the absence of the Bcl‐xL target, the mixture 1S_0_‐P_0_ and 1S_1_‐P_1_ (lane 4) generated no detectable cleavage products, indicating that spontaneous activation does not occur. Incubation of Bcl‐xL with only one FAM‐labeled arm (lanes 5 and 6) produced no observable cleavage bands, confirming that single‐arm binding is catalytically inactive. In contrast, addition of Bcl‐xL to the complete 1S_0_‐P_0_ and 1S_1_‐P_1_ pair (lanes 7–9) resulted in a clear reduction of full‐length probe and the simultaneous appearance of lower‐molecular‐weight FAM‐labeled fragments, demonstrating successful reconstitution of the catalytic core and efficient substrate hydrolysis on both arms. These results verify that the 1S‐P system exhibits strict target‐dependent activation and is capable of concurrent cleavage of the two tethered substrates only when both partzyme components and the cognate mRNA are present.

Next, we conducted fluorescence experiments using 1S_0_‐P_0_, 1S_1_‐P_1_, and 1S_2_‐P_2_ modified with distinct fluorophore‐quencher pairs: Cy5/BHQ2 for 1S_0_‐P_0_, FAM/BHQ1 for 1S_1_‐P_1_, and Cy3/BHQ2 for 1S_2_‐P_2_. As depicted in Figure S6, upon the addition of Bcl‐xL mRNA, the fluorescence intensity increased by ∼8.6‐fold for Cy5 and ∼5.4‐fold for FAM. Similarly, the addition of Bcl‐xS mRNA yielded an increased fluorescence of ∼7.9‐fold for Cy5 and ∼6.7‐fold for Cy3. In the absence of a target, only baseline fluorescence without appreciable signal enhancement was observed, confirming that the adjacent substrate‐partzyme linkage did not interfere with the DNAzyme‐catalyzed hydrolysis at either substrate. For our SUPER strategy, the partzyme probes were anchored with three substrates to ensure the dual “signal‐on” outputs. To confirm the synthesis of the partzyme‐conjugated streptavidin, we performed an electrophoresis experiment and stained the gel with GelRed (Figure 1E, left) and Coomassie Brilliant Blue R250 (Figure 1E, right), which enabled the separate visualization of DNA and protein components. The co‐migrating upper band confirmed the successful conjugation, while the faster migration of the mono partzyme‐streptavidin complex was attributed to the additional negative charges of DNA. After purification, the mono partzyme‐streptavidin conjugates were incubated with an excess of biotin‐modified substrates to achieve trivalent assembly (Figures S7 and S8). The resulting constructs showed reduced electrophoretic mobility, indicating successful substrate conjugation. The sensing performance of the assembled probes was subsequently validated through fluorescence assays. As shown in Figure 1F, the fluorescence signal increased as expected upon the addition of the corresponding target mRNAs, yielding approximately a 13.0‐fold increase in Cy5 readout and 8.8‐fold increase in FAM readout for Bcl‐xL, and a 10.5‐fold increase in Cy5 readout and 11.4‐fold increase in Cy3 for Bcl‐xS, while no discernible change was observed without the target. These findings confirm that the DNAzyme catalysis remains active after the biotin‐streptavidin conjugation and validate the feasibility of the SUPER sensing system for high‐fidelity, dual‐color imaging. Given that the streptavidin module provides both multivalent fluorophore–quencher organization and a rigid protein scaffold, we anticipated that the 3S‐P architecture would exhibit enhanced nuclease resistance. This was confirmed by serum‐tolerance assays in 10% FBS (Figure S9), whereas free P and 1S‐P probes showed 3.0‐fold and 2.7‐fold fluorescence increases due to nuclease‐mediated cleavage, 3S‐P displayed only a 1.5‐fold increase. The substantially attenuated signal rise demonstrates that multivalent streptavidin organization markedly improves probe stability, enabling more reliable intracellular operation within the SUPER platform. Beyond the intrinsic stability of the multivalent design, nuclease resistance could be further enhanced by integrating established chemical modifications, including phosphorothioate linkages [37, 38], 2′‐O‐methyl (2′‐OMe) [39] or 2′‐fluoro (2′‐F) [40] substitutions, locked nucleic acid (LNA) bases [41], peptide nucleic acid (PNA) [42], or phosphorodiamidate morpholino oligomer (PMO) [43] chemistries, which are widely used to improve oligonucleotide durability in biological environments.

### 2.3 Characterization of MnO_2_ Nanosheets for SUPER Delivery and DNAzyme Catalysis

**FIGURE 2:**
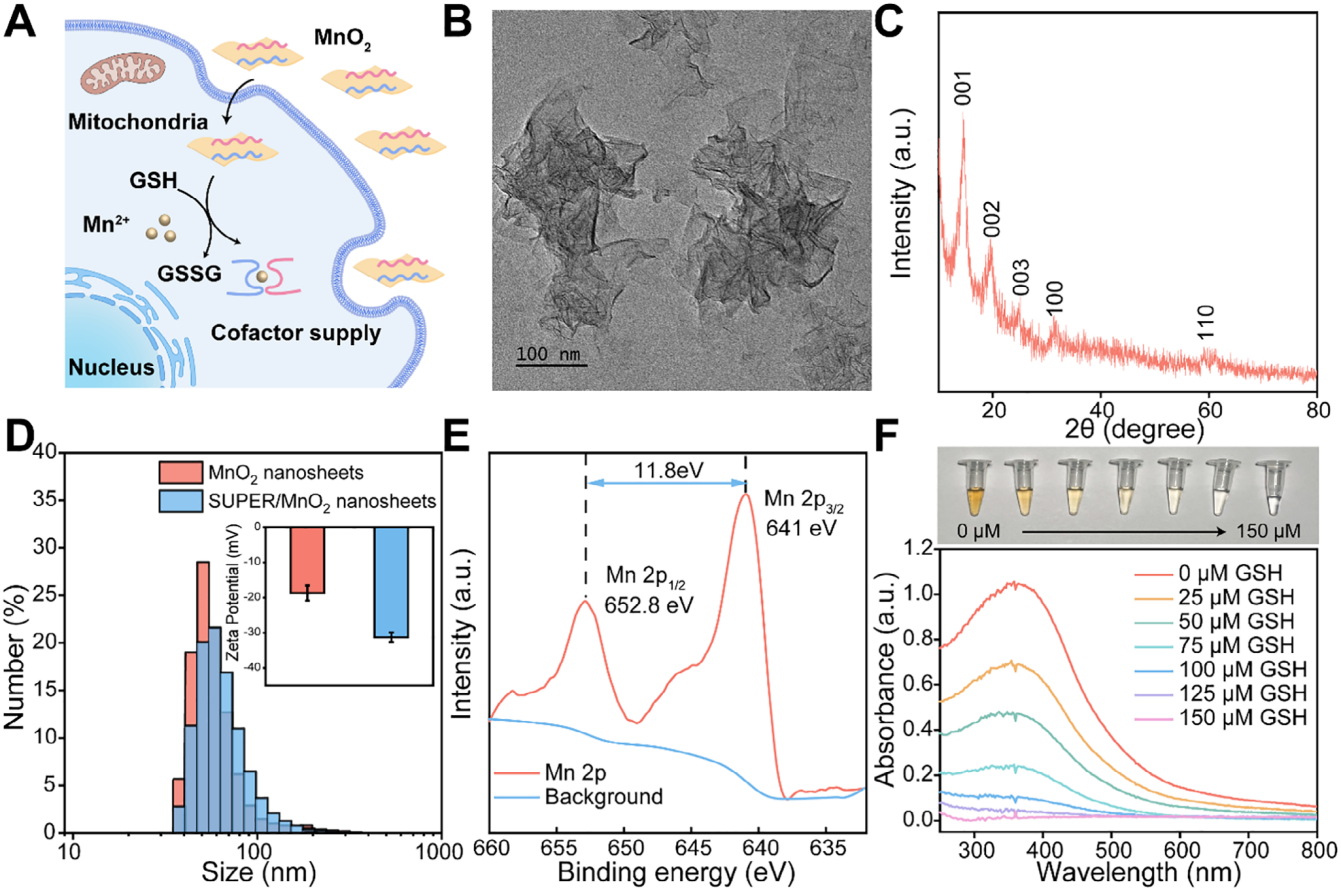
Characterization and GSH‐responsive degradation of MnO_2_ nanosheets. (A) Schematic illustration of the intracellular, GSH‐mediated decomposition of MnO_2_ nanosheets and subsequent release of SUPER probes (B) TEM image of the synthesized MnO_2_ nanosheets. Scale bar: 100 nm. (C) XRD pattern of the as‐prepared MnO_2_ nanosheets. (D) DLS measurements showing hydrodynamic diameter and zeta potential of MnO_2_ nanosheets before and after conjugation with SUPER probes. Data are presented as mean ± SD (*n* = 3 independent experiments). (E) XPS spectra of MnO_2_ nanosheets. (F) UV–vis absorption spectra and corresponding optical photographs of MnO_2_ nanosheets upon incubation with increasing concentrations of GSH (0–150 µm), demonstrating GSH‐induced degradation. Error bars represent the standard deviation (SD) from three independent experiments.

The presence of sufficient intracellular metal ion cofactors is a fundamental prerequisite for DNAzyme‐mediated biocatalysis [44]. To transfer oligonucleotide probes into cells as well as generate sufficient Mn^2+^ ions for DNAzyme cleavage reaction, the MnO_2_ nanosheets were first prepared (Figure 2A) according to previous reports [45, 46, 47]. The transmission electron microscopy (TEM) image (Figure 2B) and dynamic light scattering (DLS) size analysis (Figure 2D) revealed that the synthesized MnO_2_ nanosheets have an approximate diameter range of 100–300 nm. The phase and crystallographic structure of the MnO_2_ nanosheets were examined by X‐ray diffraction (XRD). As shown in Figure 2C, the characteristic XRD peaks at 2θ = 10.09°, 19.24°, 26.67°, 33.60°, and 61.94° of MnO_2_ corresponded to (001), (002), (003), (100), and (110) planes, respectively [48]. The X‐ray photoelectron spectroscopy (XPS) of MnO_2_ nanosheets (Figure 2E; Figure S10) presented the characteristic peaks associated with Mn 2p_1/2_, Mn 2p_3/2_, and O 1s. The peaks of Mn 2p_3/2_ and 2p_1/2,_ which are centered at 641 and 652.8 eV, with a spin‐energy separation of 11.8 eV, are in good agreement with reported data of Mn 2p_3/2_ and Mn 2p_1/2_ in MnO_2_ [49]. Additionally, the intense UV/Vis absorption with a band centered at λ = 360 nm further demonstrated the successful synthesis (Figure S11) [50]. As shown in Figure 2D, the decreased zeta potential of MnO_2_/SUPER (−31.20 mV) in comparison with bare MnO_2_ nanosheets (−18.74 mV) and the slight increase in the average diameter of nanoparticle size were observed after the adsorption of oligonucleotide probes onto the MnO_2_ nanosheets. To quantify the loading of SUPER probes onto MnO_2_ nanosheets, we adopted a standard UV–vis method commonly used in MnO_2_‐nucleic acid systems. Briefly, DNA was incubated with MnO_2_ nanosheets (100 µg mL^−1^, identical to the concentration used for cellular delivery), the suspension was centrifuged, and the remaining DNA in the supernatant was determined from A260. We observed >90% adsorption when the initial DNA concentration was 0.1–1.0 µm, followed by a gradual decline at higher concentrations, corresponding to a loading capacity of ∼10 pmol DNA probes per µg MnO_2_ under our experimental conditions (Figure S12).

Then the glutathione (GSH)‐responsive decomposition of MnO_2_ nanosheets was investigated. As shown in Figure 2F, the typical brown color of MnO_2_ nanosheets under daylight faded away with gradual GSH addition, and the characteristic absorption band of MnO_2_ nearly disappeared after reduction with 150 µm GSH, indicating the thorough destruction of MnO_2_ nanosheets. Considering the substantial GSH concentration (0.5–10 mm) inside cancer cells [51], we anticipate that the intracellular environment would effectively facilitate the complete disintegration of MnO_2_ nanosheets and the subsequent release of oligonucleotide probes. On the other hand, at least 5 mm Mg^2+^ ions are required for 8–17 DNAzyme to efficiently cleave the substrates; however, the low concentration of Mg^2+^ ions in human cells ranges from 0.2 to 2 mm [52], which is inadequate for substrate hydrolysis. By contrast, 8–17 DNAzyme achieved a much higher catalytic activity in the presence of Mn^2+^ ions, and the effective concentration of Mn^2+^ for DNAzyme is as low as 1 mm (Figure S13). The less stringent requirement of Mn^2+^ is preferable for intracellular catalysis. The surging Mn^2+^ ions upon MnO_2_ decomposition could serve as the metal ion cofactor for DNAzyme‐based catalysis. Besides, CCK‐8 biocompatibility assessment (Figure S14) demonstrated negligible cytotoxicity against MCF‐7 cells even at elevated nanosheet concentrations, fulfilling the essential biosafety criterion for live‐cell imaging applications. Taken together, MnO_2_ nanosheets were successfully prepared and proved to be an efficient nanocarrier for oligonucleotide probe delivery and cofactor supply.

### 2.4 Enhanced Robustness and Specificity in Intracellular mRNA Imaging Using the SUPER System

After verifying our strategy in vitro, we aimed to investigate the potential of the SUPER system for imaging Bcl‐x mRNA splicing variants in living cells. We selected MCF‐7 cells as the model system due to their elevated Bcl‐xL/Bcl‐xS mRNA ratio [53] and introduced the SUPER system into cells by using MnO_2_ nanosheets as carriers. The bare non‐self‐confined P system with free substrates was first investigated for intracellular mRNA imaging (Figure S15). As a negative control, the substrate probes labeled with Cy5/BHQ2 elicited minimal fluorescent signals in the cells. Conversely, intense fluorescent signals were observed in the cells treated with conventional bare P_0_ and P_1_ probes, while the cells treated with bare P_0_ and P_2_ probes generated a much weaker signal. These results preliminarily indicated a differential level of Bcl‐xL and Bcl‐xS mRNA in MCF‐7 cells. To further confirm that the fluorescence signal was derived from the DNAzyme‐induced cleavage reaction, rather than the nonspecific hydrolysis of substrates, the control probes with nucleotides mutations at the catalytic core were conducted. As expected, a substantially lower fluorescence was obtained with the mutant partzymes even with extensive incubation (Figure S16). This is reasonable since the mutant DNAzymes encoding an inactive catalytic core were unable to hydrolyze the corresponding substrates, indicating the indispensable role of the target‐reactivated DNAzyme biocatalysis for live cell imaging.

**FIGURE 3:**
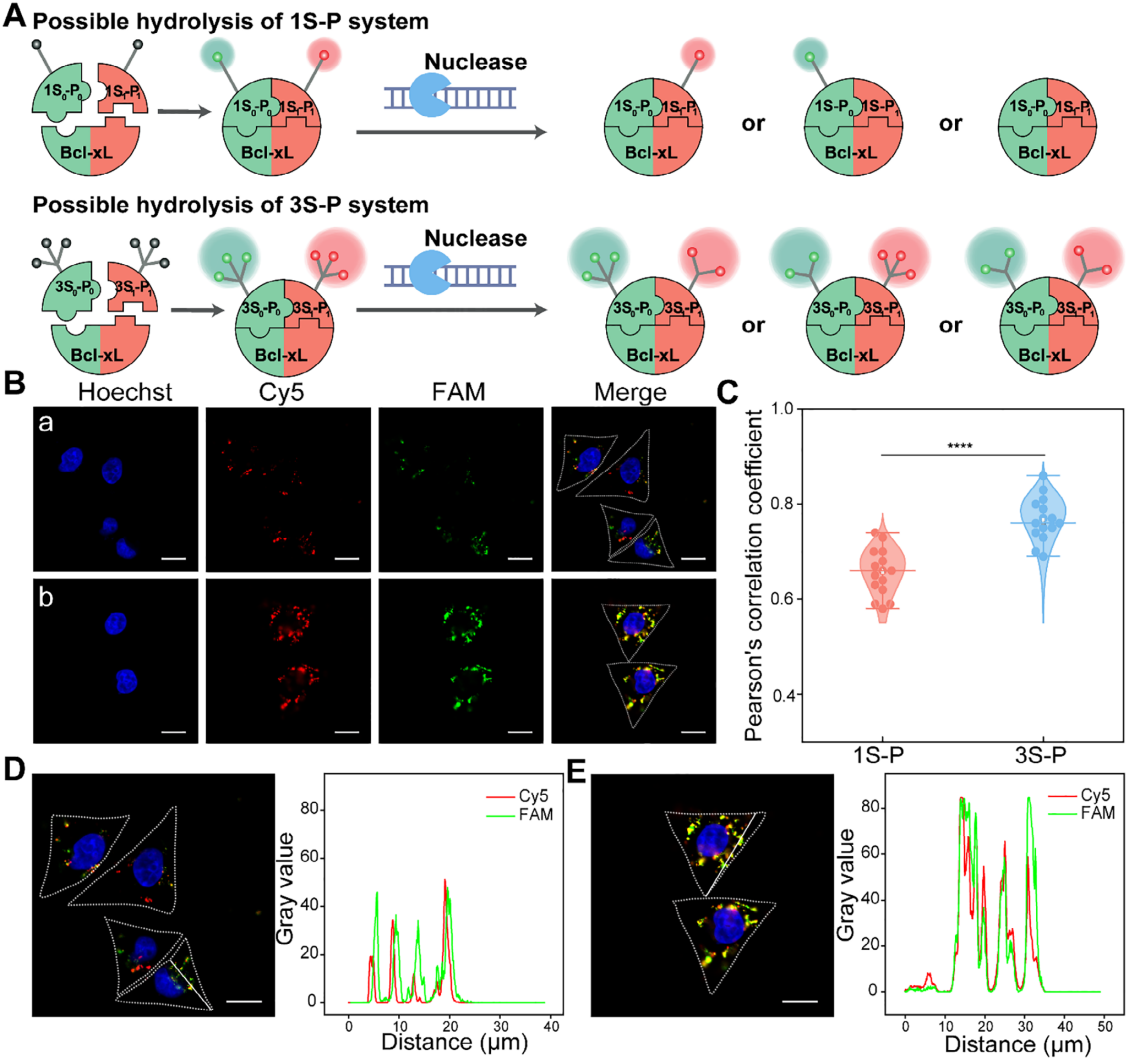
Comparison between the 1S‐P system and the 3S‐P system. (A) Schematic representation highlighting the difference in resistance to intracellular enzymatic hydrolysis between the 1S‐P and 3S‐P systems. (B) Confocal laser scanning microscopy (CLSM) images of MCF‐7 cells incubated with the 1S‐P system (a) and the 3S‐P system (b). (C) Violin plots showing the Pearson's correlation coefficients between the Cy5 and FAM channels in representative MCF‐7 cells treated with the 1S‐P and 3S‐P systems (*n* = 15 cells per group). The data points were derived from Figure S17. Data are presented as mean ± SD. p‐values were determined by a two‐tailed unpaired t‐test. ns: no significance, ^*^ *p* < 0.05, ^**^ *p* < 0.01, ^***^ *p* < 0.001, ^****^ *p* < 0.0001. The ends of the boxes represent the lower and upper quartiles, with the middle line indicating the median. Whiskers extend to 1.5× interquartile range (IQR). (D) Fluorescence intensity profiles of Cy5 (red) and FAM (green) along the white arrow in MCF‐7 cells treated with the 1S‐P system, and (E) the 3S‐P system. Scale bars: 20 µm.

Although the bare non‐self‐confined P detection system is capable of profiling RNA splicing variants in living cells, the random distribution of fluorescent dots leads to the loss of spatiotemporal information of the target mRNA. Additionally, the false‐positive signals ignited by enzymatic degradation cannot be discriminated against or eliminated. Our SUPER system with dual in situ activated fluorescence aims to visualize the spatial distribution of mRNA variants while effectively distinguishing and excluding nonspecific signals. We initially employed the 1S‐P and 3S‐P systems separately for single‐cell analysis of Bcl‐xL mRNA to illustrate the necessity of incorporating the biotin‐streptavidin system (Figure 3A). As depicted in Figure 3B, cells incubated with the 3S‐P system triggered a significantly stronger fluorescence signal compared to those treated with the 1S‐P system, which only showed slight fluorescence. This result clearly illustrated that the inclusion of the biotin‐streptavidin system significantly amplified the fluorescence intensity, thereby enhancing the sensitivity of mRNA detection. To assess the potential improvement in co‐localization between the 3S‐P and 1S‐P systems, a representative group of cells from each condition was analyzed in detail (Figure S17). As illustrated in Figure 3C, the 3S‐P system demonstrated a significantly higher co‐localization coefficient (∼0.75) than the 1S‐P system (∼0.66), suggesting a more accurate and consistent recognition of Bcl‐xL mRNA targets. The line‐scan analyses of fluorescence intensities along the white line in Figure 3D (1S‐P system) and Figure 3E (3S‐P system) further confirmed this distinction, with the 3S‐P system exhibiting well‐overlapped Cy5 and FAM signals, in contrast to the more scattered and less intense signals observed in the 1S‐P system. This difference could be attributed to the lack of sensitivity in the one‐target‐one‐signal 1S‐P system due to the absence of signal amplification, the low sensitivity, and further on‐site degradation of the already cleaved substrates could contribute to the decreased co‐localization. To clarify the importance of MnO_2_ nanosheets, we conducted control experiments in which the SUPER probes were delivered without MnO_2_. Under these conditions, cells exhibited negligible Cy5 and FAM fluorescence, in contrast to the robust dual‐color activation observed when MnO_2_ was present (Figure S18). Without this combined carrier and cofactor function of MnO_2_ nanosheets, the probes cannot efficiently enter cells nor achieve catalytic activation, resulting in minimal intracellular signal. To explore whether the co‐localization signal is caused by probe co‐aggregation within lysosomes, we assessed the co‐localization of the SUPER probe signals with lysosomes at 4 h post‐transfection. As shown in Figure S19A, the Cy5 fluorescence signals generated by the activated SUPER system exhibit minimal spatial overlap with Lysotracker‐labeled lysosomes, indicating that the majority of SUPER‐derived signals are not confined within lysosomal compartments. In addition, fluorescence line‐scan analysis was conducted across representative intracellular regions (Figure S19B,C). The intensity profiles of Cy5 and Lysotracker signals display clearly separated peak distributions, further confirming their distinct subcellular localization. These results are consistent with those presented in Figures S15 (group a) and S16, wherein minimal fluorescence was observed in the absence of the DNAzyme‐catalyzed hydrolysis. Together, they demonstrate that the co‐localization signal does not originate from the simultaneous degradation of probes within lysosomes. Furthermore, to rigorously verify the sequence specificity of target recognition, a negative control experiment was performed in which complementary antisense sequences (Anti‐P_0_ and Anti‐P_1_, Table S1) were co‐delivered with the 3S‐P system. These antisense strands are pre‐hybridized with the partzyme components, thereby preventing target engagement. As depicted in Figure S20, the inclusion of the complementary sequences resulted in a significant reduction in both Cy5 and FAM channels, thereby confirming that the generation of fluorescent signals indeed originated from the DNAzyme‐mediated biocatalytic reaction. Taken together, the enhanced co‐localization efficiency and the clear demonstration of sequence‐specific recognition confirm the high reliability and robustness of the 3S‐P system in intracellular mRNA imaging.

### 2.5 Quantitative Profiling of Bcl‐xL and Bcl‐xS mRNA Splice Variants Using the SUPER System

**FIGURE 4:**
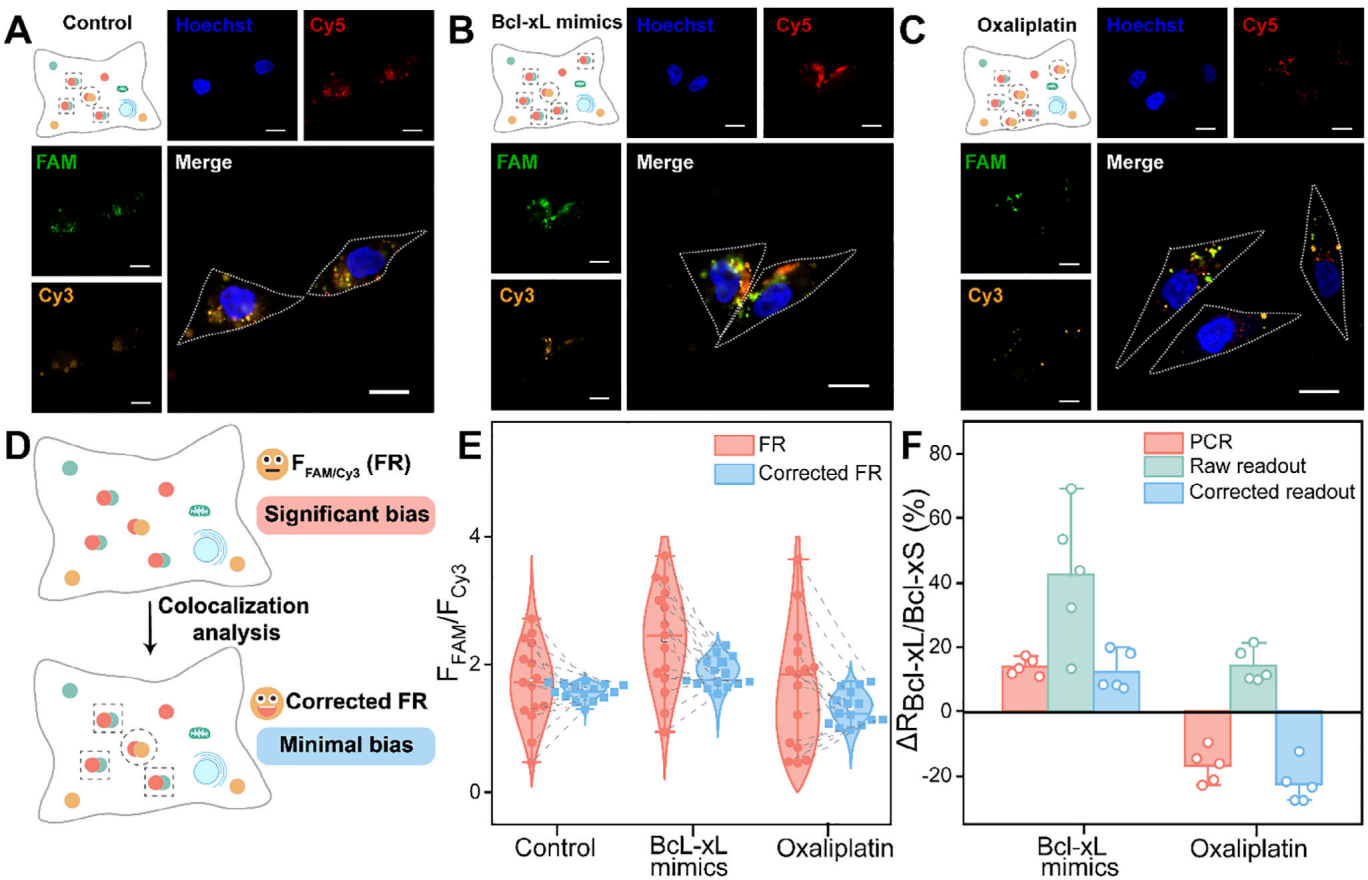
Simultaneous imaging of Bcl‐xL and Bcl‐xS mRNA in living cells using the SUPER system. (A) CLSM images showing the distribution of Bcl‐xL and Bcl‐xS mRNA in MCF‐7 cells incubated with the SUPER system. (B) CLSM images of Bcl‐xL and Bcl‐xS mRNA in MCF‐7 cells pretreated with Bcl‐xL mimics and then incubated with the SUPER system. (C) CLSM images of Bcl‐xL and Bcl‐xS mRNA in MCF‐7 cells pretreated with oxaliplatin and subsequently incubated with the SUPER system. (D) Schematic representation of the data processing pipeline, illustrating the steps taken before and after the removal of non‐co‐localized signals using co‐localization analysis. (E) Violin plots showing the calculated F_FAM_/F_Cy3_ values before (FR) and after background noise subtraction (corrected FR). Data points are derived from Figure S21. The ends of the boxes correspond to the lower and upper quartiles, with the middle line representing the median. Whiskers extend to 1.5× interquartile range (IQR). (F) Comparison of the changes in the Bcl‐xL/Bcl‐xS mRNA ratios (ΔR_Bcl‐xL/Bcl‐xS_) measured by the SUPER system before and after correction and by RT‐qPCR (*n* = 6 independent samples per group). Data are presented as mean ± standard deviation (SD). Scale bars: 20 µm.

Encouraged by the above results, we then investigated the feasibility of the SUPER system for simultaneously imaging both Bcl‐xL and Bcl‐xS mRNA, and evaluated the detection accuracy by imaging the spliced variants expressed at varied levels. The co‐localization of Cy5 and FAM signals indicates true positive signals for Bcl‐xL, analogously, and the co‐localization of Cy5 and Cy3 signals indicates true positive signals for Bcl‐xS, whereas the independent ones are considered as false‐positive signals. Specifically, cells pretreated with Bcl‐xL mimics exhibited an increased Bcl‐xL/Bcl‐xS ratio due to elevated intracellular abundance of Bcl‐xL, while cells pretreated with oxaliplatin showed a decreased Bcl‐xL/Bcl‐xS ratio as oxaliplatin was reported to alter the Bcl‐xL/Bcl‐xS mRNA ratio by increasing the relative abundance of Bcl‐xS transcripts [54]. As expected, in the control group, the intense FAM fluorescence specific to the Bcl‐xL mRNA was observed, and most of them were co‐localized with that of the Cy5. In contrast, the fluorescence of Cy3 was relatively dark, and the co‐localized signals displayed a small proportion in the overall Cy5 fluorescence (Figure 4A, detailed data in Figure S21A). This observation suggests that in the untreated control group, Bcl‐xL mRNA predominates, resulting in a stronger FAM signal for Bcl‐xL compared to the weaker Cy3 signal for Bcl‐xS, which is consistent with the expected basal distribution of these splice variants. Upon treatment with Bcl‐xL mRNA mimics (Figure 4B, detailed data in Figure S21B), the fluorescence intensity in both the Cy5 and FAM channels increased compared to the control group, reflecting the enhanced intracellular levels of Bcl‐xL. And there was no significant change in the Cy3 channel, indicating that Bcl‐xL mimics selectively upregulated Bcl‐xL expression without affecting Bcl‐xS mRNA levels. When treated with oxaliplatin, the cells exhibited a pronounced increase in the relative abundance of Bcl‐xS transcripts, evidenced by an increase in Cy3 signal and a reduction in FAM fluorescence (Figure 4C, detailed data in Figure S21C). This shift in the Bcl‐xL/Bcl‐xS ratio aligns with previous studies [54] and further validates the SUPER system's sensitivity to mRNA splice variant variations. Then, the relative fluorescence before and after correction in selected cells was quantified, and F_FAM_/F_Cy3_ (FR) were calculated (Figure 4D,E).

The detailed image analysis process is shown in Figure S22. As shown in Figure 4E, the raw F_FAM_/F_Cy3_ ratio in control cells was calculated to be 1.71 with high dispersion. After removing the scattering noises, the average ratio decreased to 1.57 with reduced data variability. Similarly, the oxaliplatin‐ and Bcl‐xL mRNA mimics‐treated group showed an aggregated statistical trend after processing. To accurately quantify the changes in Bcl‐xL/Bcl‐xS mRNA levels, a real‐time quantitative reverse transcriptase polymerase chain reaction (RT‐qPCR) assay was performed (Figure S23). Figure 4F compares the changes in ratios obtained by SUPER before (FR) and after background noise subtraction (corrected FR) with those obtained by RT‐qPCR. According to the RT‐qPCR analysis, the Bcl‐xL/Bcl‐xS mRNA levels were increased by 15.1% upon adding extra Bcl‐xL mRNA mimics, and oxaliplatin pretreatment resulted in a 17.8% decrease. These results corroborate our previous results, suggesting that Bcl‐xL mimics upregulate Bcl‐xL expression and that oxaliplatin preferentially increases Bcl‐xS mRNA levels. In contrast, the ratio changes obtained from confocal imaging showed a 40.7% increase upon addition of the Bcl‐xL mRNA mimics and a 14.2% increase following oxaliplatin treatment. The apparent overestimation of these ratio changes is likely due to signal noise and ambiguities inherent in raw data. However, after correcting for background noise, the processed data revealed a more accurate shift: an 11.5% increase upon Bcl‐xL mimic treatment and a 22.5% decrease after oxaliplatin pretreatment. Comparing these outcomes with the standard RT‐qPCR method, our proposed SUPER system achieved more reliable results, affirming its excellent intracellular accuracy for profiling mRNA spliced variants. Importantly, the robustness and quantitative fidelity demonstrated in live‐cell measurements also suggest that SUPER is inherently suited for in vitro analytical formats [55, 56, 57].

### 2.6 Dynamic Monitoring of AS1411‐Induced Bcl‐xL mRNA Degradation using the SUPER System

**FIGURE 5:**
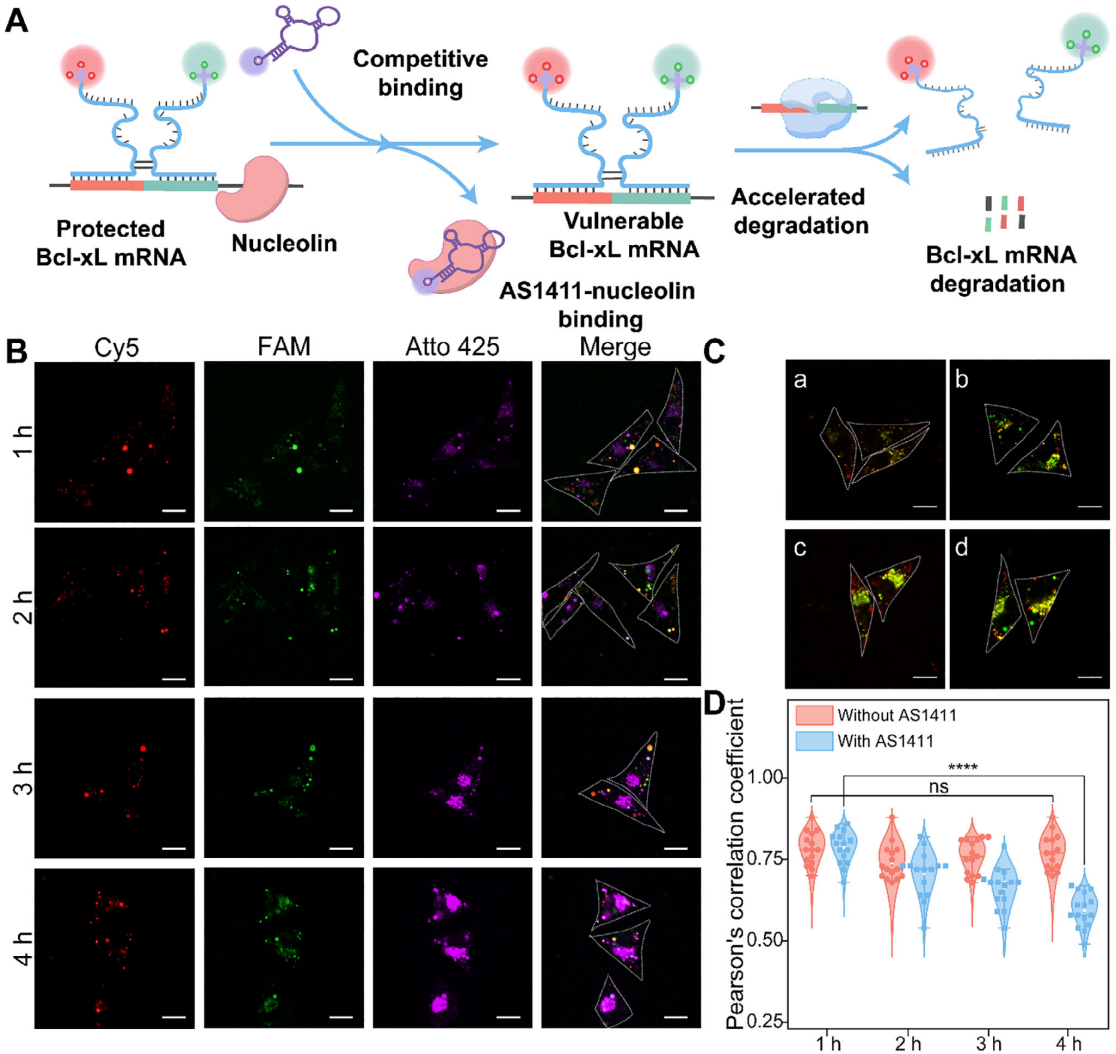
Detection of AS1411‐induced Bcl‐xL mRNA degradation using the SUPER system. (A) Schematic illustration of the mechanism by which aptamer AS1411 disrupts nucleolin binding to Bcl‐xL mRNA, thereby accelerating Bcl‐xL mRNA degradation. (B) CLSM images of MCF‐7 cells pre‐transfected with AS1411 and incubated with the 3S‐P system at various time points. (C) CLSM images of MCF‐7 cells incubated with the 3S‐P system (negative control) at different time intervals: a) 1 h, b) 2 h, c) 3 h, d) 4 h. (D) Violin plots of Pearson's correlation coefficients between the Cy5 and FAM channels (PCC_Cy5‐FAM_) in MCF‐7 cells treated with different conditions (*n* = 15 cells per group). Data points are derived from Figures S24 and S25. Data are presented as mean ± SD. p‐values were determined by a two‐tailed unpaired t‐test. ns: no significance, ^*^ *p* < 0.05, ^**^ *p* < 0.01, ^***^ *p* < 0.001, ^****^ *p* < 0.0001. The box boundaries represent the lower and upper quartiles, and the line within the box indicates the median. Whiskers extend to 1.5× interquartile range (IQR). Scale bars: 20 µm.

Building on the reliable live cell imaging, we further investigated the decay kinetics of the splicing variants through the SUPER strategy. AS1411 is a 26‐nucleotide guanine‐rich aptamer that forms a G‐quadruplex structure via dimerization and exhibits high binding affinity for nucleolin [58]. Numerous studies have shown that nucleolin selectively recognizes the AU‐rich elements (AREs) located at the 3’‐UTR of Bcl‐xL mRNA and safeguards Bcl‐xL mRNA from nuclease degradation [59, 60, 61]. Therefore, AS1411 could function as a molecular decoy, competing with Bcl‐xL mRNA for cytoplasmic nucleolin binding and decreasing the half‐life of Bcl‐xL mRNA (Figure 5A).

Based on these reports, we studied the AS1411‐induced accelerated Bcl‐xL mRNA degradation. The AS1411 with an Atto‐425 fluorophore attached to its 5′ end was pre‐transfected into MCF‐7 cells. Our approach is reliant on the recognition‐based reassembly of the partzymes probes. As a result, the swift degradation of Bcl‐xL mRNA will generate a separation between 3S_0_‐P_0_ and 3S_1_‐P_1_ probes, ultimately leading to a reduction in correlation coefficients. We first pre‐transfected MCF‐7 cells with Atto‐425‐labeled AS1411 and observed its intracellular distribution, and then introduced our SUPER system into MCF‐7 cells to monitor the co‐localization of Cy5/BHQ2‐labeled 3S‐P_0_ and FAM/BHQ1‐labeled 3S‐P_1_ over time. In the control group (Figure 5C, detailed data in Figure S24), which was not pre‐transfected with Atto‐425‐labeled AS1411, the Pearson's correlation coefficient (PCC_Cy5‐FAM_) reached a plateau at t = 1 h (∼0.78) and became saturated (t = 4 h, PCC_Cy5‐FAM_ = 0.77). The relatively stable co‐localization indicates the intact Bcl‐xL mRNA transcripts without treatment of AS1411, and the probes themselves would not destabilize the target transcripts. The cell imaging of MCF‐7 cells pre‐transfected with Atto‐425‐labeled AS1411 was shown in Figure 5B (detailed data in Figure S25). The PCC_Cy5‐FAM_ was initially high (∼0.79), indicating successful co‐localization, but gradually declined over time (∼0.70 at t = 2 h, ∼0.66 at t = 3 h, ∼0.59 at t = 4 h), suggesting accelerated Bcl‐xL mRNA degradation by AS1411. We speculate that the minimal perturbation to the specimen and the fast molecule diffusion of tiny probes were propitious for imaging live‐cell dynamics. This study demonstrates the capacity of our proposed SUPER system to detect small deviations in intracellular spliced mRNA isoforms, highlighting its potential as a robust tool for in situ dynamic tracing of spliced mRNA. More importantly, our SUPER system offers a reliable platform for elucidating the molecular mechanisms involved in mRNA splicing isoforms.

### 2.7 Spatial‐Confined SUPER System Activates AS1411 for Selective Bcl‐xL mRNA Degradation

**FIGURE 6:**
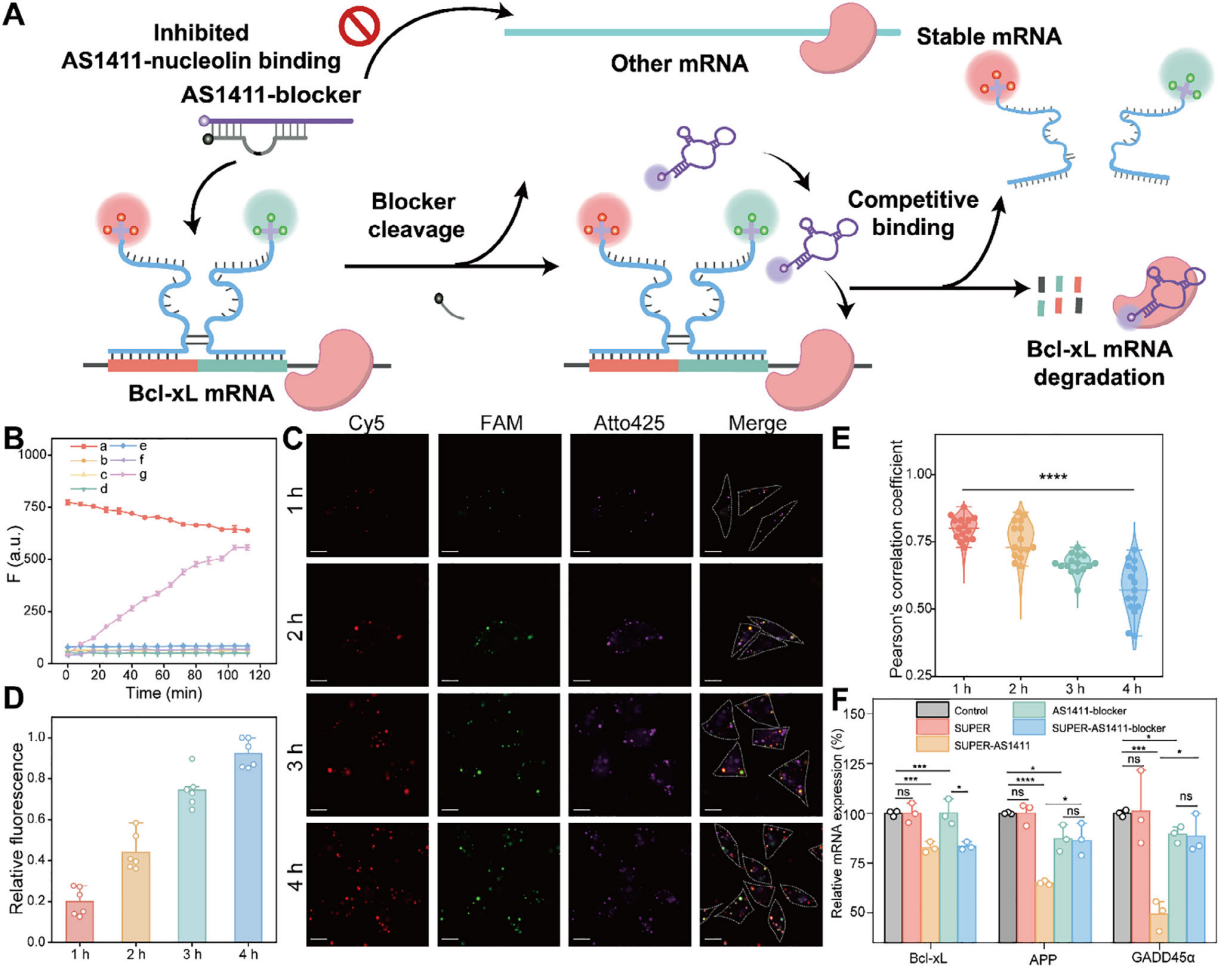
Enhanced precision targeting and degradation of Bcl‐xL mRNA by the SUPER‐AS1411‐blocker system. (A) Mechanism of Bcl‐xL mRNA degradation triggered by the SUPER‐AS1411‐blocker system. (B) Real‐time fluorescence of AS1411‐blocker cleavage in response to the SUPER system. a: AS1411, b: AS1411‐blocker, c: P_0_+AS1411‐blocker+Bcl‐xL, d: P_1_+AS1411‐blocker+Bcl‐xL, e: nucleolin+AS1411‐blocker+Bcl‐xL, f: P_0_‐P_1_+AS1411‐blocker, g: P_0_‐P_1_+AS1411‐blocker+Bcl‐xL. Data are presented as mean ± SD (*n* = 3 independent experiments). (C) Representative CLSM images of MCF‐7 cells treated with the SUPER‐AS1411‐blocker system at different time points. (D) Quantification of intracellular AS1411 fluorescence intensity at different time points (*n* = 6 cells). Data are presented as mean ± SD. (E) Violin plots showing Pearson's correlation coefficients between the Cy5 and FAM channels (PCC_Cy5‐FAM_) in MCF‐7 cells treated with the SUPER‐AS1411‐blocker system (*n* = 15 cells). Data are presented as mean ± SD. Data points were derived from Figure S27. The box boundaries represent the lower and upper quartiles, and the line within the box indicates the median. Whiskers extend to 1.5× interquartile range (IQR). (F) Relative mRNA expression levels of Bcl‐xL, APP, and GADD45α in MCF‐7 cells treated with different systems. Data are presented as mean ± SD (*n* = 3 independent experiments). p‐values were determined by a two‐tailed unpaired t‐test. ns: no significance, ^*^ *p* < 0.05, ^**^ *p* < 0.01, ^***^ *p* < 0.001, ^****^ *p* < 0.0001.

Accurate mRNA regulation without interfering with other mRNA transcripts is always preferred for clinical applications, but remains challenging due to the unintended interactions with other RNAs of similar sequences or RNA‐binding proteins. This preclinical safety liability has thwarted the clinical utility of these nucleic acid‐based drugs. In our strategy, the SUPER system was constructed at the analyte mRNA with high precision, and site‐specific activation of DNAzyme enabled local substrate cleavage. We wondered if the SUPER system with spatial‐confined catalysis could be modulated as a sensitizing antenna to activate the prodrugs in situ and curtail the non‐specific interactions. Since nucleolin was reported to bind to the AU‐rich element (ARE) in several mRNAs besides Bcl‐xL, protecting them from degradation [62, 63, 64, 65, 66, 67, 68], the site‐specific competing binding of nucleolin was expected to selectively knock down the Bcl‐xL mRNA. As shown in Figure 6A, a locking strand containing a substrate fragment (nominated as blocker, Table S1) was designed to form a double‐strand structure with AS1411. Its strong affinity would impede the native folding of AS1411 and its specific binding with nucleolin at the site of Bcl‐xL mRNA. The reconstructed DNAzyme in the SUPER system serves as an antenna to unlock the local AS1411 repetitively through the cleavage of the blocker. This AS1411‐activating antenna near the Bcl‐xL mRNA facilitated the competitive binding with the neighboring nucleolin for subsequent degradation. Meanwhile, other mRNAs were surrounded by the blocker‐caged AS1411, preventing the interaction between AS1411‐blocker and nucleolin; the spatial‐confined catalysis of the SUPER system was expected to destroy the Bcl‐xL mRNA selectively.

To test this hypothesis, a native PAGE experiment was conducted to validate the feasibility of the SUPER‐catalyzed blocker cleavage. As illustrated in Figure S26A, the AS1411 and blocker maintained a stable double‐stranded structure (lane 7). With the assistance of target Bcl‐xL, P_0_ and P_1_ could assemble into the active DNAzyme structure, catalyzing cleavage of the blocker and resulting in a cleaved fragment band with a reduced molecular weight (lane 8). The mixture of split‐DNAzyme components (P_0_, P_1,_ and target Bcl‐xL) and AS1411‐blocker duplex led to the rupture of the blocker (lane 9). The liberated AS1411 band appears faint, which is consistent with its parallel G‐quadruplex structure that stains poorly with GelRed compared with linear duplex DNA (Figure S26B). These results confirmed that the introduction of a blocker could serve as a molecular facilitator for locking up AS1411, and the reconstructed DNAzyme could hydrolyze the blocker and liberate AS1411. To monitor the above step‐by‐step reactions, we conjugated an Atto‐425 fluorophore at the 5′ end of AS1411 and a BHQ1 quencher at the 3′ end of the blocker. The assembly of AS1411‐blocker brings Atto‐425 and BHQ‐1 into close proximity, thereby producing minimal fluorescence signal. As illustrated in Figure 6B, Atto‐425‐labeled AS1411 was annealed with BHQ‐1‐labeled blocker, resulting in a subdued fluorescence signal (line b). Neither P_0_ nor P_1_ alone, even in the presence of both the AS1411‐blocker duplex and Bcl‐xL mRNA, generated any detectable fluorescence increase (lines c and d). These results confirm that the catalytic core of the split DNAzyme cannot form when either component is absent, and the blocker therefore remains intact. Likewise, nucleolin incubated with the AS1411‐blocker duplex and Bcl‐xL mRNA did not induce fluorescence recovery (line e), demonstrating that nucleolin binding alone is insufficient to release or cleave the blocker without DNAzyme activity. In sharp contrast, the complete SUPER system containing both P_0_ and P_1_ produced a robust, time‐dependent fluorescence increase when Bcl‐xL mRNA was present (line g), indicating efficient DNAzyme‐mediated hydrolysis of the blocker and release of AS1411. Notably, P_0_ and P_1_ together, but without the target mRNA, failed to activate fluorescence (line f). Collectively, these findings demonstrate that blocker cleavage occurs only when both components of the split DNAzyme are present and the correct mRNA target is provided, highlighting the stringent activation requirement and high specificity of the SUPER‐mediated mechanism. To trace the decay kinetics of Bcl‐xL mRNA induced by AS1411‐activating antenna, the changes in correlation coefficients between FAM and Cy5 channels (PCC_Cy5‐FAM_) were evaluated (Figure 6E, detailed data in Figure S27). Initially, there was significant overlap between the Cy5 and FAM fluorescence signals, with a co‐localization coefficient reaching 0.80 (t = 1 h); however, as incubation time increased, PCC_Cy5‐FAM_ gradually decreased (∼0.76 at t = 2 h, ∼0.67 at t = 3 h, ∼0.57 at t = 4 h). The decline in co‐localization coefficients clearly demonstrated the degradation behavior of Bcl‐xL mRNA, which implied that the spatial‐confined activation of AS1411 was sufficient for Bcl‐xL mRNA destabilization. Consistent with this trend, the fluorescence recovery of AS1411 showed a marked time‐dependent increase (Figure 6D). The Atto‐425 signal rose progressively from 1 h to 4 h, indicating continuous release of AS1411 from the quenched AS1411‐blocker duplex as the SUPER system cleaved the blocker. This steady increase in AS1411 fluorescence provides direct evidence of ongoing aptamer activation within cells and further corroborates that AS1411 activation and Bcl‐xL mRNA degradation occur concurrently through the SUPER‐mediated mechanism.

To assess the degradative tendency of our AS1411‐activating antenna, we employed the RT‐qPCR method to determine the expression levels of the reported nucleolin‐binding mRNAs, including growth arrest and DNA‐damage‐inducible gene45α (GADD45α) [62], interleukin 2 (IL‐2) [63], β‐globin [64], amyloid precursor protein (APP) [65], gastrin [66], CD40 ligand (CD154) [67], renin (REN) [68] and Bcl‐xL [59, 60, 61]. As illustrated in Figure 6F, no significant differences were observed in the expression levels of three mRNAs (Bcl‐xL, APP, and GADD45α) after treatment with the SUPER system alone compared to the control group, suggesting that the introduction of the SUPER system does not exert interference on the stability of these three mRNAs. And due to the low expression levels in MCF‐7 cells, other mRNAs, including IL‐2, β‐globin, gastrin, CD154, and REN, were not detected (data not shown). Upon transfection with AS1411, notable reductions were observed for all three detected mRNAs (a 17.2% decrease in Bcl‐xL mRNA, a 35.0% decrease in APP mRNA, and a 50.8% decrease in GADD45α mRNA, respectively), confirming that the competitive binding by AS1411 leads to destabilization of these target mRNAs. Conversely, incubation with AS1411‐blocker alone resulted in minimal changes in the expression level of Bcl‐xL mRNA, which was in line with expectations, while reductions of 12.7% and 10.6% were observed for APP and GADD45α mRNA levels, respectively. We estimate that the decrease in APP and GADD45α expression can be attributed to the differential influence of nucleolin on target mRNAs. Compared to the group incubated with AS1411‐blocker alone, the additional SUPER‐based antenna system resulted in a significant 16.7% decrease in Bcl‐xL expression. In contrast, no prominent reduction in APP and GADD45α mRNA levels was observed. These results collectively demonstrate that the integration of the AS1411‐blocker and SUPER system induces degradation of Bcl‐xL mRNA exclusively. Meanwhile, from the RT‐qPCR results (Figure 6F) and co‐localization analysis (Figure 6E, detailed data in Figure S27), we also observed a comparable decay rate for Bcl‐xL mRNA in the group incubated with SUPER‐AS1411 and SUPER‐AS1411‐blocker. To further validate whether AS1411‐induced Bcl‐xL mRNA degradation is generalizable beyond MCF‐7 cells, we extended the analysis to HeLa cells using the same SUPER‐based antenna systems. As shown in Figure S28, consistent with our initial findings, SUPER‐mediated release of AS1411 led to a selective reduction of Bcl‐xL mRNA in HeLa cells, whereas free AS1411 caused a broader downregulation of nucleolin‐associated transcripts, and the AS1411‐blocker alone produced minimal effects. The undisturbed knockdown observed in the SUPER‐AS1411‐blocker system implied that the SUPER system could be swiftly recruited onto the target sites, and the reconstituted sensitizing antenna could activate and supply AS1411 efficiently. As the pronounced selectivity toward specific mRNA transcripts is the crucial demand for the clinical translation of nucleic acid therapeutics, the SUPER‐based in situ activating antenna system provides a promising toolbox for precise gene therapy. More importantly, the SUPER‐based antenna could discriminate and fine‐tune the specific mRNA splicing variants. Precise cytoplasmic modulation of alternative splicing mRNA isoforms provides an attractive method of therapeutic mRNA splicing modulation, as the conventional small molecule modulators toward constitutive RNA processing machinery usually lead to ambiguous disruption and unwanted cytotoxicity, and the splicing‐switching oligonucleotides are confronted with the restricted nuclear entry and the nonspecific perturbations due to the prevalence of off‐target interactions. Further, coupling this precise in situ activation mechanism with the exosome‐based delivery technologies will be key to translating its high specificity from cellular models into effective in vivo therapies [69, 70].

## 3 Conclusion

In summary, inspired by the multicomponent biological assembly, a simple yet versatile SUPER platform was developed for interference‐free, reliable, and highly robust mRNA splicing imaging. The single mRNA variant was regarded as an individual component for concerted reconfiguration of DNAzyme and subsequent cooperative catalysis; the stringent global folding of DNAzyme ensured accurate recognition of alternative splicing. Besides, the reconstitution of the catalytic nanosystem enables in situ mutual hydrolysis, the following dual activated fluorescence is propitious for error‐correcting imaging. In our strategy, the dynamics of inherent mRNA decay could be further monitored through co‐localization analysis, which has been rarely achieved. Our strategy is applicable for mechanism studies of apoptosis‐regulatory mRNA splicing, and paves a new way for reducing the false‐positive signals in the conventional nucleic acid‐based imaging. Under the guidance of stringent recognition, the analyte‐aided reconstituted SUPER system could be easily adapted as the sensitizing antenna for spatial‐confined activation of oligonucleotide‐based prodrugs. Given the fact that the conventional splicing‐switching oligonucleotides were confronted with the restricted nuclear entry and the inherent off‐target interactions, our in situ exclusive intervention of specific splicing isoforms in the cytoplasm holds great potential for intelligent theragnostics with efficacy and precision.

## 4 Experimental Section

### 4.1 Synthesis and DNA Loading of MnO_2_ Nanosheets

MnO_2_ nanosheets were synthesized according to reported methods by oxidative precipitation of MnCl_2_ followed by ultrasonication‐assisted exfoliation. DNA loading onto MnO_2_ nanosheets was quantified using a UV–vis absorbance method. Briefly, MnO_2_ nanosheets (100 µg mL^−1^) were incubated with DNA probes in HEPES buffer at 37°C, and the concentration of unbound DNA was determined from the absorbance at 260 nm after centrifugation. Under the conditions used for cellular delivery, MnO_2_ nanosheets exhibited >90% DNA adsorption efficiency, corresponding to a loading capacity of approximately 10 pmol DNA per µg MnO_2_ nanosheets.

### 4.2 Fluorescence Assays

Fluorescence measurements were performed in HEPES buffer (10 mm, pH 7.0) containing NaCl and MnCl_2_ at 37°C unless otherwise noted. The activity of the bare P system was evaluated by incubating split partzymes (200 nm) with fluorophore/quencher‐labeled substrates (200 nm) in the presence or absence of target mRNAs (10 nm). Target‐induced reassembly of the split DNAzyme resulted in efficient substrate cleavage and fluorescence activation. Sensitivity and specificity were assessed by recording fluorescence responses to varying concentrations of target mRNAs (0–100 nm) as well as to mismatched or noncognate RNA sequences. Multivalent probes (1S‐P and 3S‐P systems) were further evaluated by dual‐color fluorescence readouts using comparable probe concentrations. Metal‐ion‐dependent DNAzyme catalysis was examined by comparing reactions supported by Mg^2+^ or Mn^2+^ (0.25–5 mm), and SUPER‐mediated blocker cleavage was verified using fluorophore‐labeled AS1411/blocker duplexes.

### 4.3 Gel Electrophoresis

Denaturing and native polyacrylamide gel electrophoresis were employed to characterize probe assembly and DNAzyme activity. Denaturing PAGE was used to verify target‐induced reassembly and catalytic cleavage of split DNAzymes, as well as to compare metal‐ion‐dependent catalytic efficiency. Native PAGE was used to confirm the formation and purification of partzyme‐streptavidin conjugates, the successful multivalent substrate coupling, and the SUPER‐mediated cleavage of blocker duplexes. Electrophoresis was typically performed at a constant voltage of 100 V in 1× TBE buffer. Gel images were visualized by fluorescence detection or GelRed staining. Detailed gel compositions, sample concentrations, and running times are provided in the Supporting Information.

### 4.4 Cell Culture

MCF‐7 cells were cultured in Dulbecco's modified Eagle's medium (DMEM) supplied with 10% fetal bovine serum, 1% penicillin–streptomycin, and incubated at 37°C in a humidified incubator with 5% CO_2_.

### 4.5 Confocal Fluorescence Imaging

MCF‐7 cells were seeded on glass‐bottom confocal dishes and cultured to 60%–70% confluence prior to imaging experiments. Cells were incubated with MnO_2_‐loaded SUPER probes in Opti‐MEM, containing MnO_2_ nanosheets (100 µg mL^−1^) and SUPER probes (200 nm), to enable intracellular delivery and DNAzyme activation. After incubation, cells were washed to remove extracellular probes, stained with Hoechst 33342, and subjected to confocal laser scanning microscopy (CLSM). Mutant split DNAzymes were used as negative controls to confirm that fluorescence signals originated from DNAzyme‐mediated cleavage. The performance of different probes (bare P, 1S‐P, and 3S‐P systems) was compared under identical imaging conditions. In addition, detection accuracy and functional responsiveness of the SUPER system were evaluated using oxaliplatin treatments or mRNA mimics. AS1411‐mediated regulation and decay kinetics of Bcl‐xL mRNA were further investigated by imaging cells pre‐transfected with AS1411 or AS1411/blocker duplexes (500 nm). Detailed imaging conditions and treatment protocols are provided in the Supporting Information.

### 4.6 RT‐qPCR Analysis

Total RNA was extracted from MCF‐7 and HeLa cells using an RNA extraction kit according to the manufacturer's instructions. Reverse transcription was performed to generate cDNA, followed by quantitative PCR using SYBR Green‐based detection. RT‐qPCR was employed to quantify the expression levels of Bcl‐xL, Bcl‐xS, and additional control transcripts, with GAPDH used as an internal reference gene for normalization. Relative mRNA expression levels were calculated using standard comparative threshold cycle methods. Primer sequences and detailed reaction conditions are provided in the Supporting Information.

### 4.7 Statistical Analysis

All quantitative data are presented as mean ± standard deviation (SD). The sample size (n) for each experiment is specified in the corresponding figure legends and refers to the number of independent experiments or biological replicates, as indicated. Statistical significance was assessed using unpaired two‐tailed Student's t‐tests. *P* < 0.05 (^*^), *p* < 0.01 (^**^), *p* < 0.001 (^***^), and *p* < 0.0001 (^****^) were considered statistically significant. Raw fluorescence and imaging data were processed using ImageJ, including background subtraction and region‐of‐interest (ROI) selection as described in the Supporting Information. Statistical analyses and data visualization were performed using GraphPad Prism software. The meaning of statistical significance symbols is provided in the figure legends.

## Conflicts of Interest

The authors declare no conflicts of interest.

## Supporting information




**Supporting file**: advs73977‐sup‐0001‐SuppMat.docx.

## Data Availability

The data that support the findings of this study are available in the supplementary material of this article.
