# Supplementary material for: A Modular DNAzyme for Precise Visualization and Intervention of Alternative Splicing Isoforms in Live Cells
Source: Adv Sci (Weinh). 2026 Jan 21;13(18):e17895. doi: 10.1002/advs.202517895 (PMC13042592; doi:10.1002/advs.202517895)
Supplement: Supplementary file 1 — Supporting file: advs73977‐sup‐0001‐SuppMat.docx. [file ADVS-13-e17895-s001.docx]

Supporting Information

**A Modular DNAzyme System for Precise Visualization and Intervention of Alternative Splicing Isoforms in Live Cells**

*Mengru Lin, Jiale Sun, Yuqing Mao,* *Yuhao Tang, Fuan Wang,* Zhihong Liu,* and Jing Wang**

M. Lin, J. Sun, Y. Mao, Y. Tang, J. Wang, Prof. Z. Liu

College of Health Science and Engineering, Hubei Province Key Laboratory of Biotechnology of Chinese Traditional Medicine, Hubei University

Wuhan, Hubei 430062 (P. R. China)

E-mail: janewang@hubu.edu.cn, [zhhliu@whu.edu.cn](mailto:zhhliu@whu.edu.cn)

M. Lin, Prof. Z. Liu, Prof. F. Wang

Department of Gastroenterology, Zhongnan Hospital of Wuhan University, College of Chemistry and Molecular Sciences, Wuhan University

Wuhan, Hubei 430072 (P. R. China)

E-mail: fuanwang@whu.edu.cn, zhhliu@whu.edu.cn

* To whom correspondence should be addressed.

**Table of Contents**

**Experimental Section** S-3

**Figure S1**. Bcl-x alternative splicing and design of isoform-specific SUPER probes. S-9

**Figure S2**. Design of partzyme pairs for bare P system biosensing. S-10

**Figure S3**. Gel analysis of bare P system activity S-11

**Figure S4**. Fluorescence responses of bare P system S-12

**Figure S5**. Validation of the cleavage reaction of the 1S-P system S-13

**Figure S6**. Fluorescence response of 1S-P system S-14

**Figure S7**. Gel analysis of the formation of 1S-P system S-15

**Figure S8**. PAGE analysis of biotin-DNA and streptavidin complexes S-16

**Figure S9**. Serum stability of P, 1S-P, and 3S-P probes S-17

**Figure S10**. XPS spectra of MnO_2_ nanosheets S-18

**Figure S11**. UV-vis absorption spectrum of MnO_2_ nanosheets S-19

**Figure S12**. DNA loading on MnO₂ nanosheets S-20

**Figure S13**. M^2+^-dependent DNAzyme biocatalysis S-21

**Figure S14**. Cell cytotoxicity of MnO_2_ nanosheets S-22

**Figure S15**. CLSM images of cells with bare P system S-23

**Figure S16**. CLSM images of cells with mutant partzymes S-24

**Figure S17**. CLSM images of cells with 1S-P and 3S-P systems. S-25

**Figure S18**. Cell imaging with and without MnO₂ delivery. S-27

**Figure S19**. CLSM images of SUPER probes and Lysotracker at 4 h post-transfection S-28

**Figure S20**. CLSM images of Anti-P0- and Anti-P1-pretreated cells with 3S-P system S-29

**Figure S21**. CLSM imaging of cells with 3S-P system under various pre-treatments S-30

**Figure S22**. Scheme of the protocol for image analysis S-33

**Figure S23**. RT-qPCR analysis of Bcl-xL/Bcl-xS mRNA expression in cells S-34

**Figure S24**. Time-dependent CLSM imaging of cells treated with 3S-P system S-35

**Figure S25**. CLSM images of AS1411-pretreated cells with 3S-P system over time S-40

**Figure S26**. PAGE analysis of the blocker cleavage reaction S-44

**Figure S27**. CLSM of AS1411-blocker-pretreated cells with 3S-P system over time S-45

**Figure S28**. mRNA expression changes in HeLa cells S-49

**Figure S29**. All the original gel images S-50

**Table S1**. Sequences of DNA oligonucleotides S-51

**Experimental Section**

1. **Materials**

All DNA oligonucleotides were purchased from Sangon Biotech Co. Ltd. (Shanghai, China). 4-(2-hydroxyethyl) piperazine-1-ethanesulfonic acid sodium salt (HEPES), sodium chloride (NaCl), manganese chloride (MnCl_2_), hydrogen peroxide (H_2_O_2_), tetramethylammonium hydroxide (TMAOH) and glutathione (GSH) were purchased from Aladdin Reagent (Shanghai, China). Streptavidin (SA) and Coomassie Brilliant Blue R250 were purchased from Sigma-Aldrich (St Louis, MO, USA). 6 × loading buffer was purchased from TaKaRa Biotechnology Co., Ltd (Dalian, China). GelRed^TM^ nucleic acid stain was purchased from Biotium (USA). Lipofectamine-3000 was purchased from Thermo Fisher Scientific. Cell Counting Kit-8 (CCK-8), Hoechst 33342, and Lysotracker green were obtained from Beyotime Institute of Biotechnology (Shanghai, China). Human breast cancer cells (MCF-7 cells) were obtained from Procell Life Science &Technology Co., Ltd. (Hubei, China). Phosphate buffered saline (PBS), Dulbecco’s Modified Eagle’s Medium (DMEM), trypsin-EDTA, and penicillin-streptomycin were obtained from Gibco (NY, USA). Fetal bovine serum (FBS) was purchased from PAN-Biotech (Germany).

1. **Synthesis of MnO_2_ nanosheets**

MnO_2_ nanosheets were synthesized according to previous reports. Briefly, a mixture of 20 mL aqueous solution containing 3 wt% H_2_O_2_ and 0.6 M tetramethylammonium hydroxide (TMAOH) was rapidly added to a stirring solution of MnCl_2_ (10 mL, 0.3 M) within 15 s. The dark brown solution was stirred vigorously overnight in the open air at room temperature. The bulk MnO_2_ was subsequently obtained via centrifugation, washed with water and methanol at least three times. The as-prepared bulk MnO_2_ was then dried at 60 °C. To obtain the MnO_2_ nanosheets, 10 mg bulk MnO_2_ was dispersed in 20 mL water under ultrasonication (>10 h).

1. **Quantification of SUPER probe adsorption onto MnO_2_ Nanosheets**

The loading capacity of MnO_2_ nanosheets for nucleic acid probes was quantified following established UV-vis absorbance protocols used in MnO_2_-DNA systems. Briefly, MnO_2_ nanosheets were dispersed in HEPES buffer (10 mM, pH 7.0, 1 M NaCl, 20 mM MnCl_2_) at a final concentration of 100 μg mL⁻¹, identical to that used for further cellular delivery experiments. A series of DNA probes (0.1-3.5 μM) were mixed with the MnO_2_ suspension and incubated for 30 min at 37 ℃ to ensure adsorption equilibrium. Following incubation, the samples were centrifuged at 12,000 rpm for 10 min, and the supernatants were collected for UV-vis analysis. The concentration of unbound DNA was determined from the absorbance at 260 nm using a calibration curve generated from standard DNA solutions. The adsorption efficiency (%) was calculated as:

Adsorption Efficiency =$\frac{\text{C}\text{0}-\text{C}\text{supernatant}}{\text{C}\text{0}}$×100%

where C_0_ is the initial DNA concentration and C_supernatant_ is the concentration remaining after adsorption. Under these conditions, MnO_2_ nanosheets showed >90% adsorption efficiency for DNA concentrations between 0.1 and 1.0 μM, followed by a gradual decline at higher concentrations as surface binding sites approached saturation. Based on these measurements, the loading capacity was determined to be approximately 10 pmol DNA per μg MnO_2_ nanosheets.

1. **Characterization**

The morphology and size of MnO_2_ nanosheets were characterized by a JEM-2100 transmission electron microscope (TEM, JEOL, Japan) with an acceleration voltage of 200 kV. The valence state of the elements was determined by X-ray photoelectron spectrometer (ESCALAB250Xi, Thermo Fisher Scientific, USA). Powder X-ray diffraction (XRD) patterns were acquired using an XPert Pro X-ray diffractometer (PANalytical B.V., Holland) with a 2θ range of 10°-80° with Cu Kα irradiation (λ = 1.5406 Å). The fluorescence monitoring experiments were recorded on a RF-6000 fluorophotometer (Shimadzu, Japan). The absorption spectra were measured by the UV 2550 UV-Vis spectrophotometer (Shimadzu, Japan). The Zetasizer Nano ZS90 (Malvern, UK) was used for dynamic light scattering (DLS) experiments. The CCK-8 assay was conducted on a Multiskan Mk3 microplate reader (Thermo Fisher Scientific, USA). Confocal fluorescence cell imaging was performed by the LSM 880 Microscope (Zeiss, Germany).

1. **Denaturing Polyacrylamide Gel Electrophoresis (dPAGE) Assay**

For feasibility validation of the bare P system, corresponding partzymes (200 nM) and substrate (200 nM) were incubated with or without target mRNA (200 nM) in HEPES buffer (10 mM, pH 7.0, 1 M NaCl, 20 mM MnCl_2_) at 37 °C for 3 h. 10 μL of the above-mentioned samples was mixed with 2 μL of 6 × loading buffer and run on a 15% denaturing polyacrylamide gel in 1×TBE buffer at 100 V for 1.5 h. The gel was stained with GelRed and imaged by FluorChem FC3 (Protein Simple, USA). For the feasibility validation of the bare P system, FAM-labeled 1S-P_0_ (200 nM) and FAM-labeled 1S-P_1_ (200 nM) were incubated with Bcl-xL mRNA (200 nM) in HEPES buffer (10 mM, pH 7.0, 1 M NaCl, 20 mM MnCl_2_) at 37 ℃ for 3h, and the dPAGE experiment was conducted using the same protocol as described above. To analyze the catalytic efficiency of 8-17 DNAzyme with M^2+^ (Mn^2+^ or Mg^2+^), 8-17 DNAzyme (200 nM) and substrate (200 nM) were incubated with different concentrations (0.25, 0.5, 1, 2, or 5 mM) of Mg^2+^ HEPES buffer or Mn^2+^ HEPES buffer at 37 ℃ for 3 h, and the dPAGE experiment was performed using the same protocol as described above.

1. **Native** **Polyacrylamide Gel Electrophoresis (PAGE) Assay**

To prove the successful separation between partzyme-streptavidin and excess streptavidin, native gel electrophoresis (12%) was implemented. The mixture was prepared in reaction buffer containing the biotin-modified P_0_ or P_1_ (200 nM) and excess streptavidin. The prepared samples were analyzed by electrophoresis in 1 × TBE buffer at 100 V for 2.5 h. The gel was stained with Gel Red and Coomassie Brilliant Blue R250, respectively. Then FluorChem FC3 (ProteinSimple, USA) was utilized to analyze the stained gel. To confirm the successful trivalent coupling of partzyme-Streptavidin and biotin-modified substrate, the purified partzyme-Streptavidin and biotin-modified substrate were incubated in PBS for 3 h. After ultracentrifugation, PAGE analysis was conducted using the same protocol as described above. For validation of the feasibility of the SUPER-catalyzed blocker cleavage, AS1411 (200 nM) was mixed with blocker (200 nM) and annealed to form the AS1411-blocker duplex. Then P_0_ (200 nM), P_1_ (200 nM), Bcl-xL (200 nM) were incubated with the above AS1411-blocker duplex in HEPES buffer (10 mM, pH 7.0, 1 M NaCl, 20 mM MnCl_2_) at 37 ℃ for 3 h, and PAGE analysis was conducted using the same protocol as described above.

1. **Fluorescence Assays**

Unless specifically indicated, the fluorescence experiments were performed in HEPES buffer (10 mM, pH 7.0, 1 M NaCl, 20 mM MnCl_2_) at 37 ℃. For partzymes sequence optimization, different combinations of partzymes (200 nM) were incubated with Cy5/BHQ2-labeled sub (200 nM) and Bcl-xL mRNA (10 nM) for 1 h. For feasibility validation of bare P system, corresponding partzymes (200 nM) and Cy5/BHQ2-labeled sub (10 nM) were incubated with or without target mRNA (10 nM) for 1 h. To achieve optimal split-DNAzyme catalytic activity, P_0_ and P_1_ at different concentrations (200, 100, 66.7, 50, 40, 10 nM) were incubated with Cy5/BHQ2-labeled substrate (200 nM) and target Bcl-xL mRNA for 1 h. For the sensitivity detection, Cy5/BHQ2-labeled substrate (200 nM) and corresponding SUPER probes (66.7 nM) were incubated with various concentrations (0–100 nM) of target mRNA. For the specificity analysis, Cy5/BHQ2-labeled substrate (200 nM) and specific partzymes (66.7 nM) were incubated with mismatched sequences (10 nM) and control mRNAs (10 nM) for 1 h. For feasibility validation of 1S-P system, Cy5/BHQ2-labeled 1S_0_-P_0_ (200 nM) and FAM/BHQ1-labeled 1S_1_-P_1_ (or Cy3/BHQ2-labeled 1S_2_-P_2_, 200 nM) were incubated with target mRNA (10 nM) for 3 h. For feasibility validation of 3S-P system, Cy5/BHQ2-labeled 3S_0_-P_0_ (200 nM) and FAM/BHQ1-labeled 3S_1_-P_1_ (or Cy3/BHQ2-labeled 3S_2_-P_2_, 200 nM) were incubated with target mRNA (10 nM) for 3 h. For M^2+^-dependent DNAzyme biocatalysis, 8-17 DNAzyme (200 nM) and Cy5/BHQ2-labeled substrate (10 nM) were incubated with different concentrations (0.25, 0.5, 1, 2, or 5 mM) of Mg^2+^ HEPES buffer or Mn^2+^ HEPES buffer for 1 h. To confirm the SUPER-mediated hydrolysis of blocker, 200 nM Atto-425-labeled AS1411 was annealed with BHQ1-labeled blocker for the formation of AS1411-blocker duplex and then the AS1411-blocker was incubated with P_0_ (200 nM), P_1_ (200 nM) and Bcl-xL (200 nM) in HEPES buffer for 2 h.

1. **Cell culture**

MCF-7 cells were cultured in Dulbecco’s modified Eagle’s medium (DMEM) supplied with 10% fetal bovine serum, 1% penicillin-streptomycin and incubated at 37 °C in a humidified incubator with 5% CO_2_.

1. **Confocal fluorescence imaging**

For the bare P imaging system, MCF-7 cells were seeded and cultured in a 35-mm glass-bottom confocal dish (NEST) until they reached 60-70% confluence over a period of 24 hours.

The culture medium was then replaced with 400 μL of Opti-MEM containing MnO₂ nanosheets (100 μg/mL) and Cy5/BHQ2-labeled substrate probes (200 nM), along with corresponding SUPER probes. After a 4-hour incubation, the cells were washed three times with PBS to remove non-internalized probes, stained with Hoechst 33342, and subjected to confocal fluorescence imaging. To confirm that the fluorescence signal was derived from the DNAzyme-induced cleavage reaction, MCF-7 cells were incubated with mutant split DNAzyme in 400 μL of Opti-MEM for 4 hours and then subjected to confocal laser scanning microscopy (CLSM) imaging using the same protocol. To compare the 1S-P and 3S-P systems, MCF-7 cells were incubated with either the 1S-P or 3S-P system in 400 μL of Opti-MEM for 4 hours, followed by CLSM imaging using the same protocol. To evaluate the detection accuracy of the SUPER system, MCF-7 cells were pretreated with oxaliplatin or Bcl-xL mimics, then incubated with the 3S-P system in 400 μL of Opti-MEM for 4 hours, followed by CLSM imaging using the same protocol. For AS1411-induced accelerated Bcl-xL mRNA degradation imaging, MCF-7 cells were pre-transfected with Atto-425-labeled AS1411 (500 nM) using Lipofectamine 3000, then incubated with the SUPER system in 400 μL of Opti-MEM for 4 hours, followed by CLSM imaging using the same protocol. For decay kinetics analysis of Bcl-xL mRNA induced by AS1411-activating antennae, Atto-425-labeled AS1411 was annealed with BHQ1-labeled blocker to generate the AS1411-blocker duplex. MCF-7 cells were pre-transfected with the AS1411-blocker duplex (500 nM) using Lipofectamine 3000, then incubated with the SUPER system in 400 μL of Opti-MEM for 4 hours, followed by CLSM imaging using the same protocol. To clarify the intracellular localization of the MnO_2_ nanosheet-mediated delivery at 4 h post-transfection, colocalization between the SUPER probes and Lysotracker Green was evaluated. Cells were then incubated with Opti-MEM containing MnO_2_ nanosheets (100 μg mL⁻¹) preloaded with the Cy5-labeled SUPER probes (3S_0_-P_0_ and 3S_1_-P_1_) under the same conditions as used for imaging experiments. After 4 h incubation at 37 °C, cells were washed three times with PBS to remove extracellular probes. For live-cell lysosomal staining, cells were incubated with Lysotracker Green (75 nM in culture medium) for 30 min at 37 °C, washed with PBS, and immediately imaged in fresh medium.

1. **Image analysis for dual-color co-localization and fluorescence quantification**

Fluorescence image analysis was performed using ImageJ. All raw confocal images acquired from the FAM, Cy5 and Cy3 channels were first converted into 8-bit format, and a cellular region of interest (ROI) was manually selected to exclude extracellular background. Co-localized fluorescence signals were identified through logical operations implemented in ImageJ’s Image Calculator. For Bcl-xL imaging, dual-positive pixels were defined as those simultaneously present in both the Cy5 and FAM channels. For Bcl-xS imaging, dual-positive pixels were defined as those simultaneously present in both the Cy5 and Cy3 channels. AND-logic images were subsequently generated using the “AND” operation in Image Calculator to retain only these dual-positive populations. Fluorescence intensities from the resulting dual-positive masks were quantified to compute the raw fluorescence ratio (R0 = F_FAM_ / F_Cy3_ before background subtraction) and the signal-corrected fluorescence ratio (RS = F_FAM_ / F_Cy3_ after background subtraction), following the workflow illustrated in Figure S21. Co-localization between the Cy5 and FAM channels (or Cy5 and Cy3) was quantified using Pearson's correlation coefficient (PCC) computed with ImageJ’s Coloc 2 plugin. PCC was calculated on a per-cell basis using the background-subtracted 8-bit channel images within the cellular ROI.

1. **RT-qPCR analysis**

Total cellular RNAs were extracted from MCF-7 and HeLa cells using the TRIpure Total RNA Extraction Reagent (ELK Biotech Co., Ltd.) according to the manufacturer’s instructions. The cDNA samples were prepared using the reverse transcription (RT) reaction with EntiLink™ 1st Strand cDNA Synthesis Kit (ELK Biotech Co., Ltd.). Quantitative PCR analysis was carried out using the EnTurbo™ SYBR Green PCR SuperMix (ELK Biotech Co., Ltd.). The primers (from 5’ to 3’) used in this experiment were:

Bcl-xL forward primer: 5’- CCAGGGACAGCATATCAGAGC -3’,

Bcl-xL reverse primer: 5’- AAGTATCCCAGCCGCCGTT -3’.

Bcl-xS forward primer: 5’- TCCCCATGGCAGCAGTAAAG -3’,

Bcl-xS reverse primer: 5’- TCCACAAAAGTATCCTGTTCAAAGC -3’.

GADD45α forward primer: 5’- AGAAGACCGAAAGCGACCCC-3’,

GADD45α reverse primer: 5’- GATGTTGATGTCGTTCTCGCAG-3’

APP forward primer: 5’- GAAGTTGAGCCTGTTGATGCC-3’,

APP reverse primer: 5’- ATTGCACCTTTGTTTGAACCC -3’.

IL-2 forward primer: 5’- TGGAGCATTTACTGCTGGATTTAC-3’,

IL-2 reverse primer: 5’- TTCAGTTCTGTGGCCTTCTTGG-3’.

β-globin forward primer: 5’- GGCTCATGGCAAGAAAGTGC-3’,

β-globin reverse primer: 5’- ATTGGACAGCAAGAAAGCGA-3’.

CD154 forward primer: 5’- CCAGATGATTGGGTCAGCACT-3’,

CD154 reverse primer: 5’- ATCCTTCACAAAGCCTTCAAACTG-3’.

gastrin forward primer: 5’- CGACTGTGTGTGTATGTGCTGAT-3’,

gastrin reverse primer: 5’- GGTAGCTCCAGGTCCCTGTT-3’.

REN forward primer: 5’- GACACCCAGTACTATGGCGAGAT-3’,

REN reverse primer: 5’- GAGGGTGAGTTCTGTTCCATTGT -3’.

GAPDH forward primer: 5’- GGAAGCTTGTCATCAATGGAAATC -3’,

GAPDH reverse primer: 5’- TGATGACCCTTTTGGCTCCC -3’.


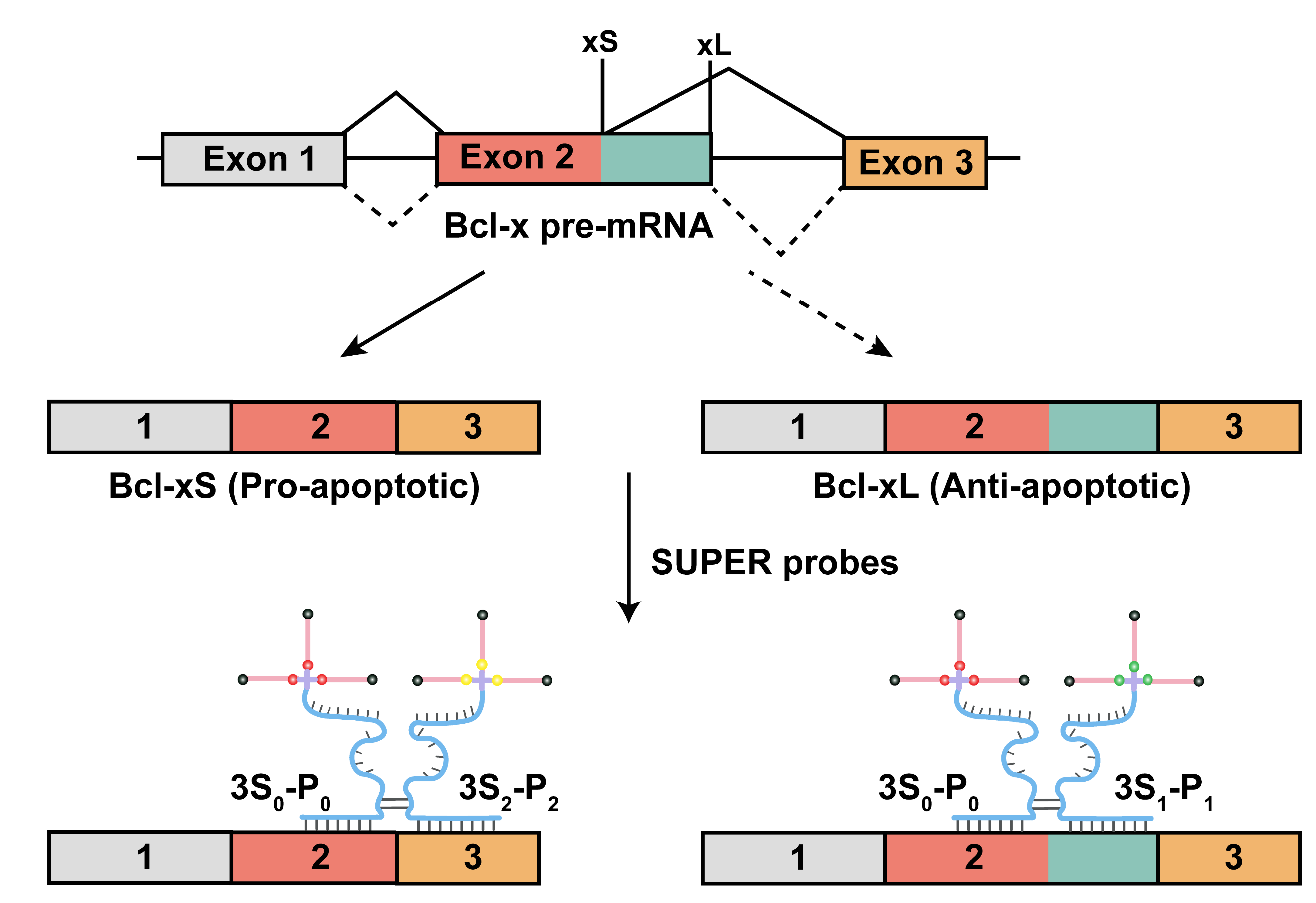


**Figure S1.** Schematic illustration of Bcl-x pre-mRNA alternative splicing and binding sites of the designed SUPER probes.

Bcl-x pre-mRNA undergoes alternative splicing to produce the anti-apoptotic Bcl-xL isoform and the pro-apoptotic Bcl-xS isoform. The SUPER probes were rationally engineered to recognize isoform-specific exon-exon junctions with high precision. A common probe (3S_0_-P_0_) targets the shared exon 2 region (red). To selectively detect Bcl-xL, an additional 3S_1_-P_1_ was designed to hybridize to the partial intronic sequence between exon 2 (red) and exon 3 (green). In contrast, selective visualization of Bcl-xS is achieved using 3S_2_-P_2_, which binds the unique exon 3 region (yellow). This isoform-restricted probe design enables highly specific imaging of the two splice variants.


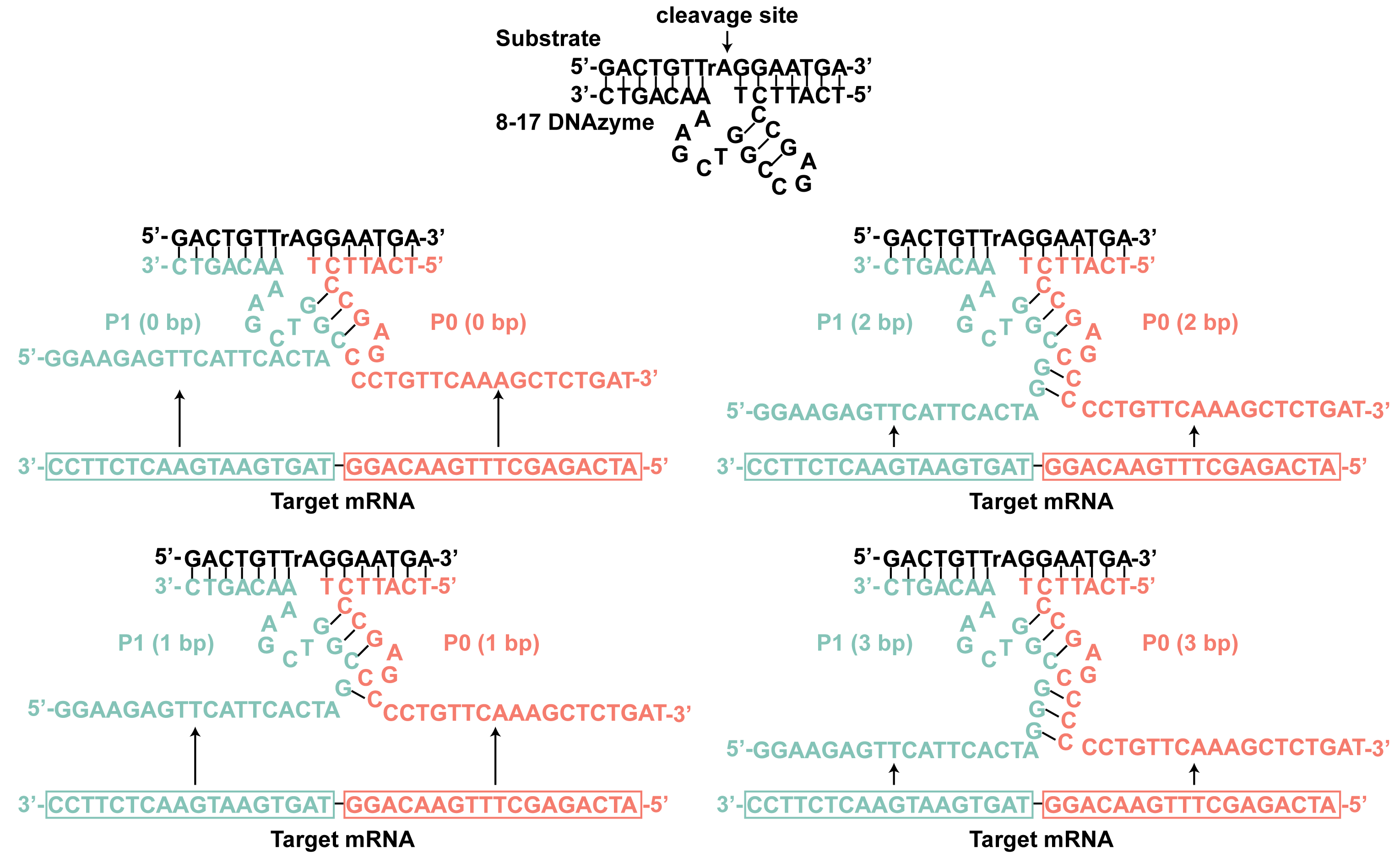


**Figure S2.** Design of partzyme pairs for bare P system biosensing.


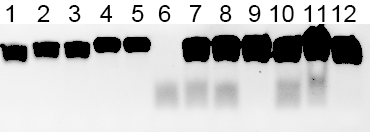


**Figure S3.** Feasibility of the bare P system by denaturing polyacrylamide gel electrophoresis (dPAGE). Lane 1: P_0_, lane 2: P_1_, lane 3: P_2_, lane 4: Bcl-xL, lane 5: Bcl-xS, lane 6: substrate, lane 7: P_0_+P_1_+substrate, lane 8: P_0_+P_1_+Bcl-xL+substrate (without Mn^2+^), lane 9: P_0_+P_1_+Bcl-xL+substrate (with Mn^2+^), lane 10: P_0_+P_2_+substrate, lane 11: P_0_+P_2_+Bcl-xS+substrate (without Mn^2+^), lane 12: P_0_+P_2_+Bcl-xS+substrate (with Mn^2+^). The reaction buffer used for incubation in lane 8 and lane 11 lacked MnCl_2_ addition whereas other groups were incubated in a reaction buffer containing MnCl_2_ (10 mM, pH 7.0, 1 M NaCl, 20 mM MnCl_2_).

As shown in **Figure S3**, the substrate bands in lane 7 and lane 10 exhibited minimal changes following incubation with their respective partzymes, indicating that the cleavage reaction of DNAzyme was inhibited due to the absence of the corresponding spliced mRNAs. Moreover, in the presence of the target mRNAs but without the Mn^2+^ cofactor, no discernible alteration was observed in the substrate bands, highlighting the indispensable role of Mn^2+^ as a cofactor for facilitating DNAzyme cleavage. Upon introduction of specific spliced mRNAs in conjunction with Mn^2+^, complete disappearance of substrate bands (lane 9 and lane 12) confirmed initiation of the cleavage reaction.


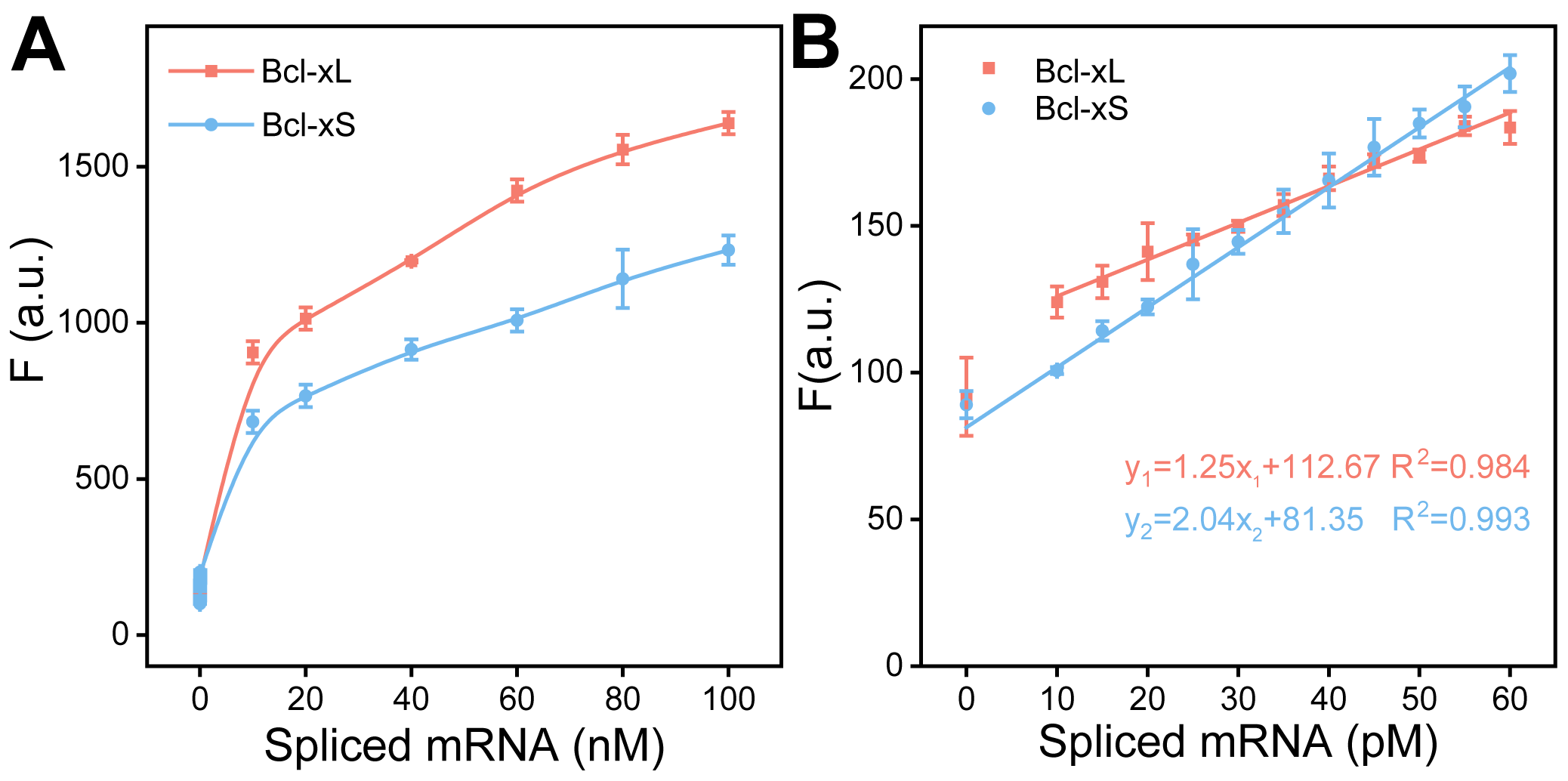


**Figure S4.** Fluorescence responses of the bare P detection system in HEPES buffer (10 mM, pH 7.0, 1 M NaCl, 20 mM MnCl_2_) with various concentrations of specific spliced mRNA. (A) Real-time fluorescence monitoring of the bare P detection system over different concentrations of Bcl-xL and Bcl-xS mRNAs, ranging from 0.01 to 100 nM. (B) The corresponding calibration plots of the Bcl-xL and Bcl-xS mRNAs in the range from 0 to 60 pM. The calibration equation for Bcl-xL mRNA is y_1_ = 1.25x_1_ + 112.67, where y_1_ is the fluorescence and x_1_ is the concentration of Bcl-xL. R^2^ = 0.984. The calibration equation for Bcl-xS mRNA is y_2_ = 2.04x_2_ + 81.35, where y_2_ is the fluorescence and x_2_ is the concentration of Bcl-xS mRNA. R^2^ = 0.993. Error bars represent the standard deviation (SD) from three independent experiments.


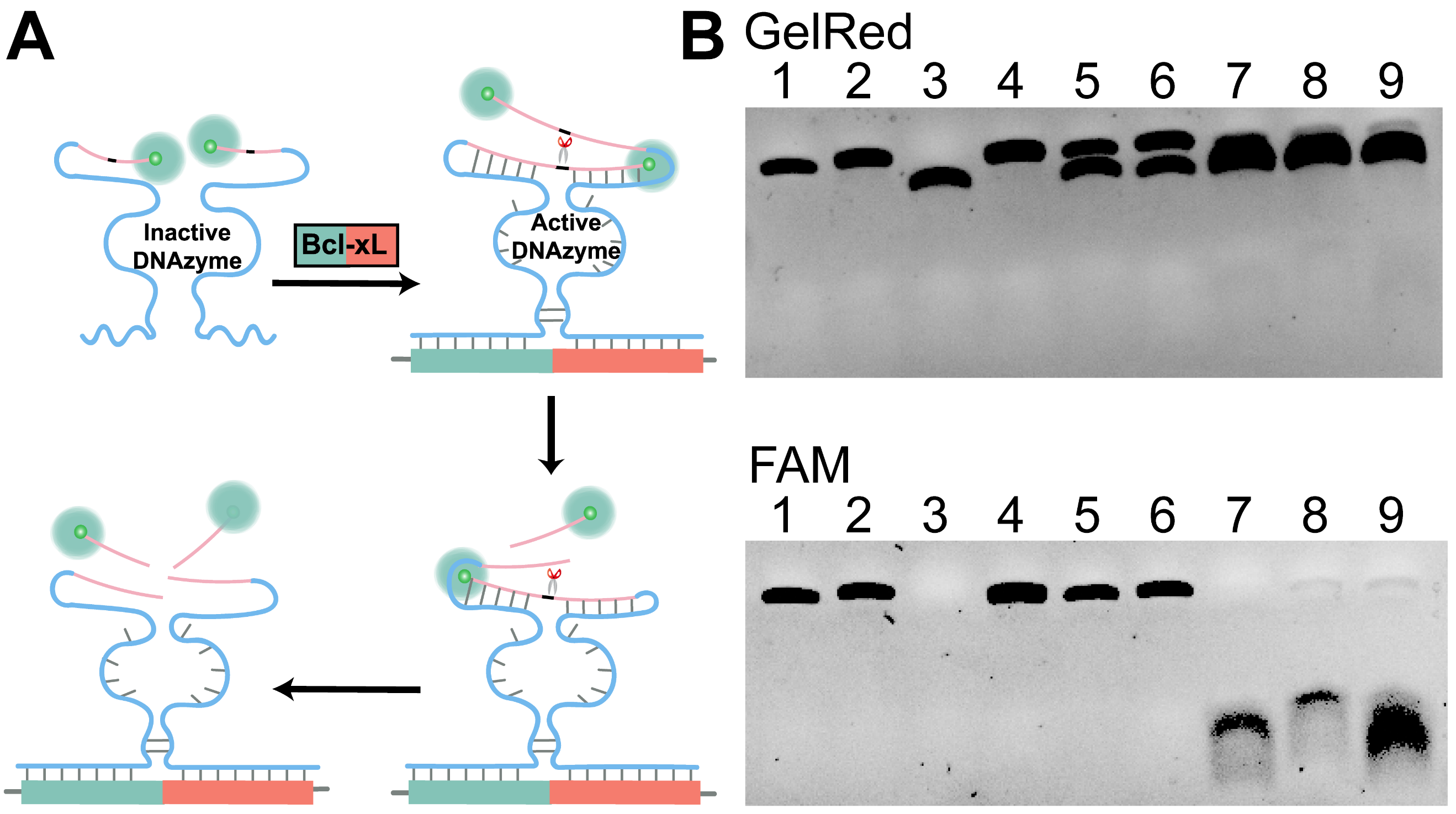


**Figure S5.** Validation of the cleavage reaction of the 1S-P system. (A) Schematic illustration of the activation mechanism of the FAM-labeled 1S-P system. (B) dPAGE analysis of the cleavage reaction. Lane 1: FAM-1S_0_-P_0_, lane 2: FAM-1S_1_-P_1_; lane 3: Bcl-xL, lane 4: FAM-1S_0_-P_0_+FAM-1S_1_-P_1_ (no target), lane 5: Bcl-xL+FAM-1S_0_-P_0_, lane 6: Bcl-xL+FAM-1S_1_-P_1_, lane 7: Bcl-xL+FAM-1S_0_-P_0_+1S_1_-P_1_, lane 8: Bcl-xL+1S_0_-P_0_+FAM-1S_1_-P_1_, lane 9: Bcl-xL+FAM-1S_0_-P_0_+FAM-1S_1_-P_1_.

As shown in **Figure S5A**, in the presence of Bcl-xL mRNA, hybridization of the target-binding domains induces reconstitution of the DNAzyme catalytic core, enabling cleavage at the ribonucleotide adenosine (rA) sites on both 1S_0_-P_0_ and 1S_1_-P_1_. As shown in **Figure S5B**, cleavage fragments appear only when both partzymes and the target mRNA are present, confirming strict target-dependent activation of the 1S-P system. Notably, cleavage products derived from both 1S_0_-P_0_ and 1S_1_-P_1_ are observed, demonstrating that the reconstituted DNAzyme catalyzes efficient hydrolysis on both substrate-anchored arms.


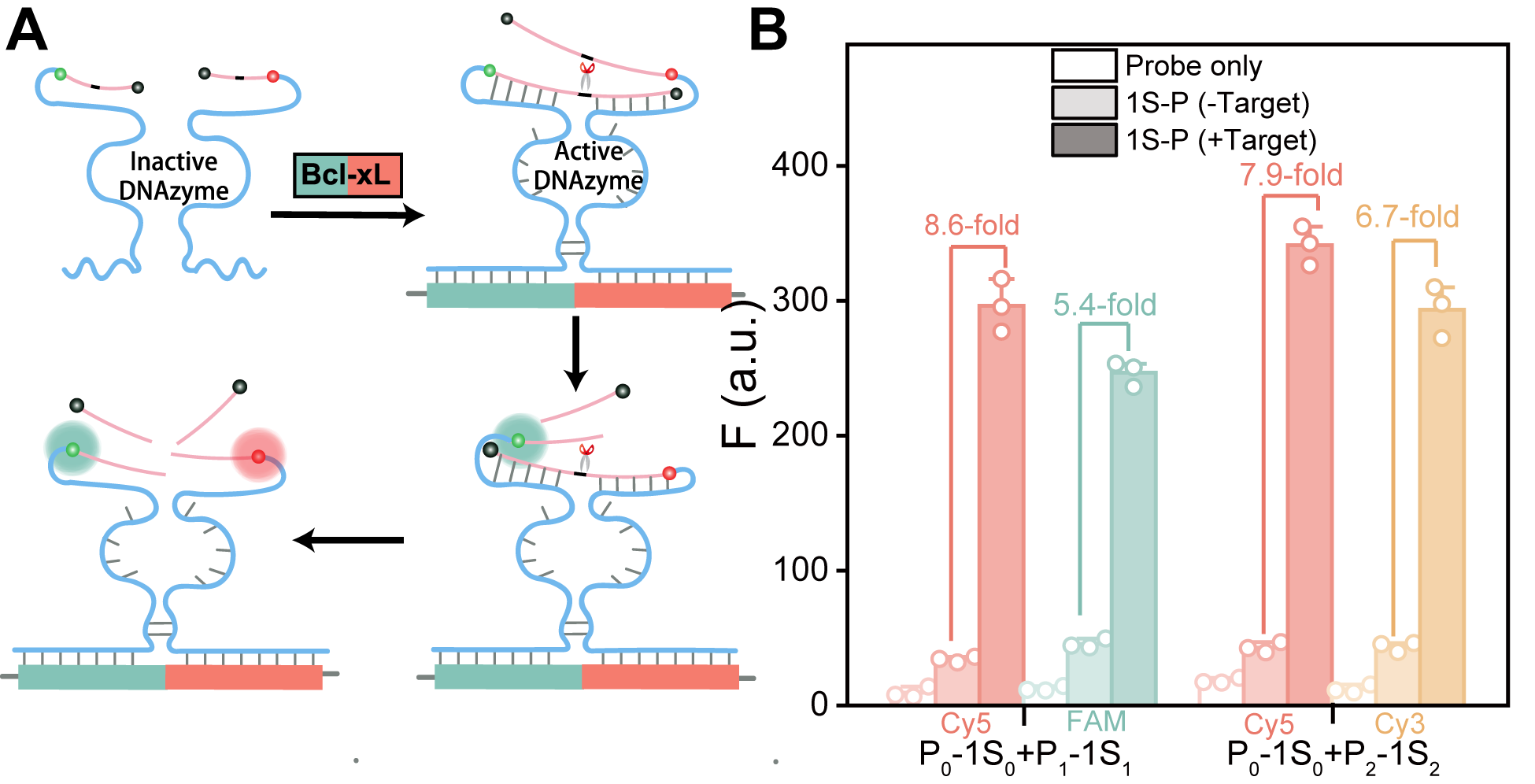


**Figure S6.** Fluorescence response of the 1S-P system. (A) Schematic illustration of the target-triggered activation of the 1S-P system, taking Bcl-xL mRNA as an example. (B) Fluorescence intensities of the 1S-P probes in the presence and absence of target mRNA.

As shown in **Figure S6A**, in the absence of Bcl-xL mRNA, the split DNAzyme remains inactive. When Bcl-xL hybridizes with the target-recognition domains of 1S_0_-P_0_ and 1S_1_-P_1_, the catalytic core is reassembled, enabling cleavage of the Cy5-labeled 1S_0_-P_0_ and FAM-labeled 1S_1_-P_1_ substrates and releasing fluorescent fragments. As shown in **Figure S6B**, robust fluorescence enhancement is observed only with Bcl-xL mRNA, producing ~8.6-fold (Cy5) and ~5.4-fold (FAM) increases for the 1S_0_-P_0_/1S_1_-P_1_ pair, and ~7.9-fold (Cy5) and ~6.7-fold (Cy3) for the 1S_0_-P_0_/1S_2_-P_2_ pair. In the absence of target, all probes exhibit baseline fluorescence. Error bars represent the standard deviation (SD) from three independent experiments.


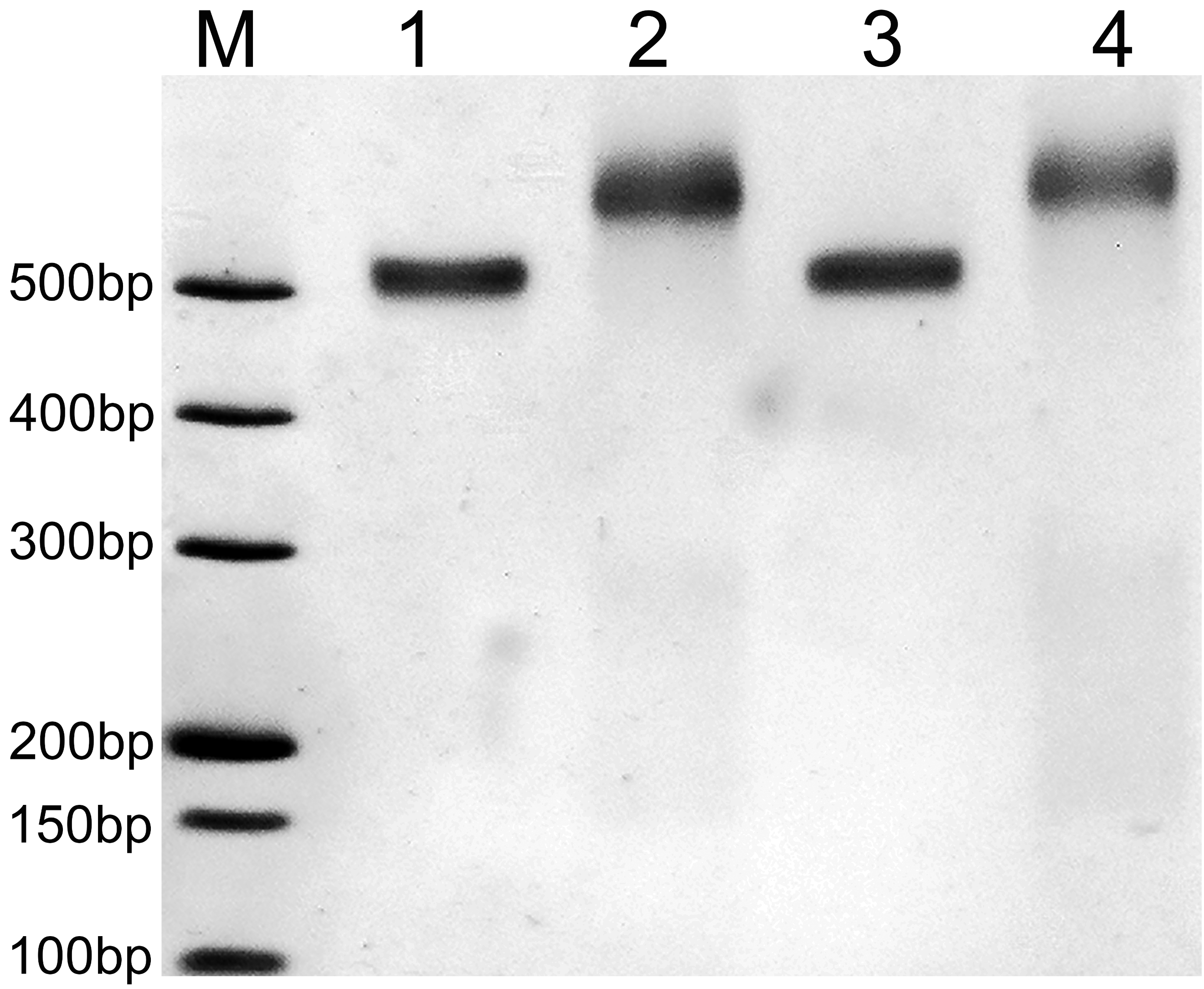


**Figure S7.** PAGE analysis of the formation of 3S-P probes in HEPES buffer (10 mM, pH 7.0, 1 M NaCl, 20 mM MnCl_2_), lane 1: P_0_-streptavidin, lane 2: P_0_-streptavidin+biotin-modified substrate, lane 3: P_1_-streptavidin, lane 4: P_1_-streptavidin+biotin-modified substrate.


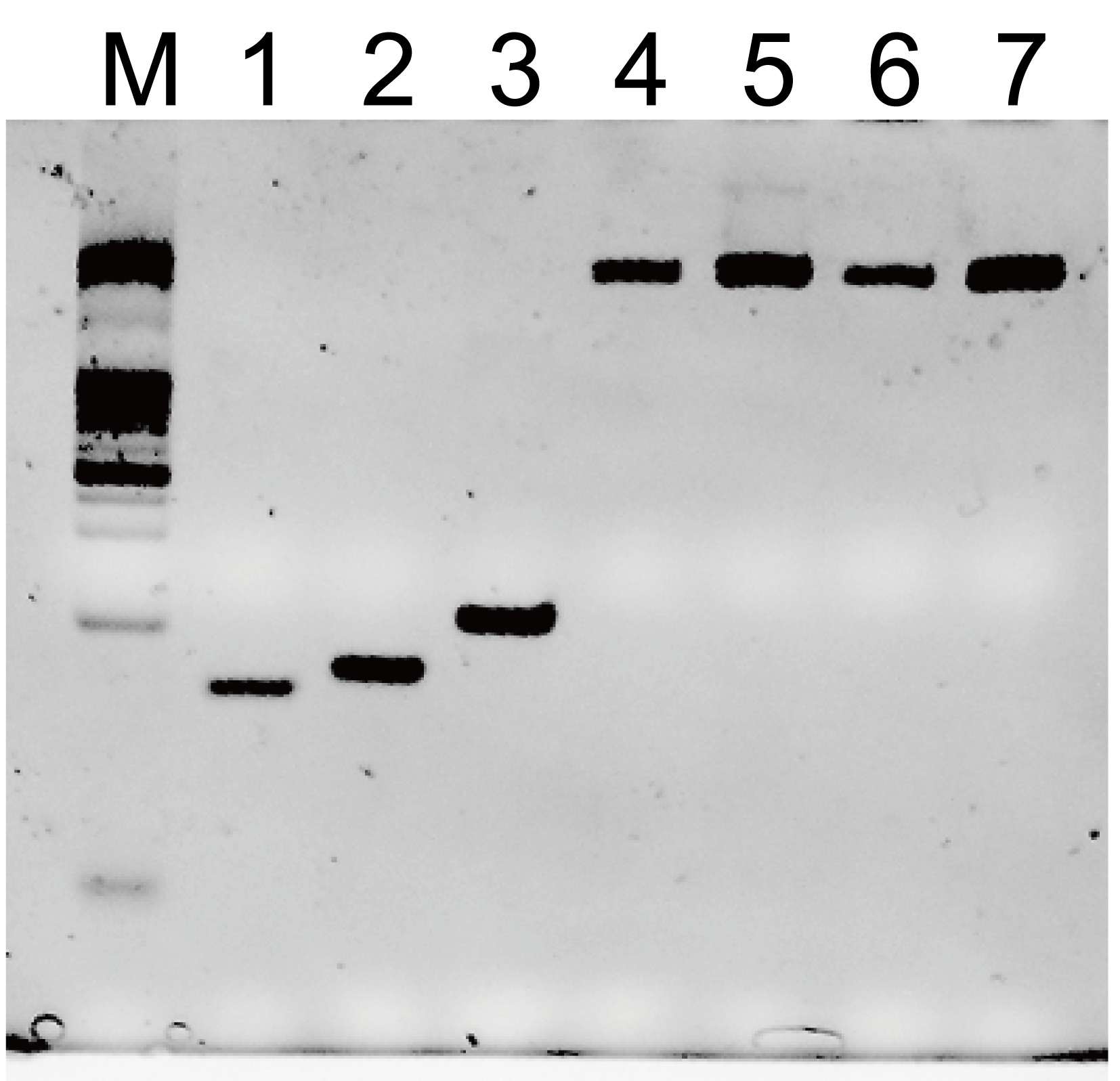


**Figure S8**. PAGE analysis of the electrophoretic behavior of biotin-modified oligos and streptavidin conjugates. Lane 1: biotin-P_0_, lane 2: biotin-P_1_, lane 3: biotin-sub, lane 4: P_0_-streptavidin, lane 5: P_0_-3S_0_, lane 6: P_1_-streptavidin, lane 7: P_1_-3S_1_.

As shown in **Figure S8**, when biotin-P_0_ (lane 1) and biotin-P_1_ (lane 2) have already migrated to the lower region, the much slower P_0_-streptavidin (lane 4) and P_1_-streptavidin (lane 6) remain near the top, while free streptavidin has not yet fully entered the gel (data not shown).


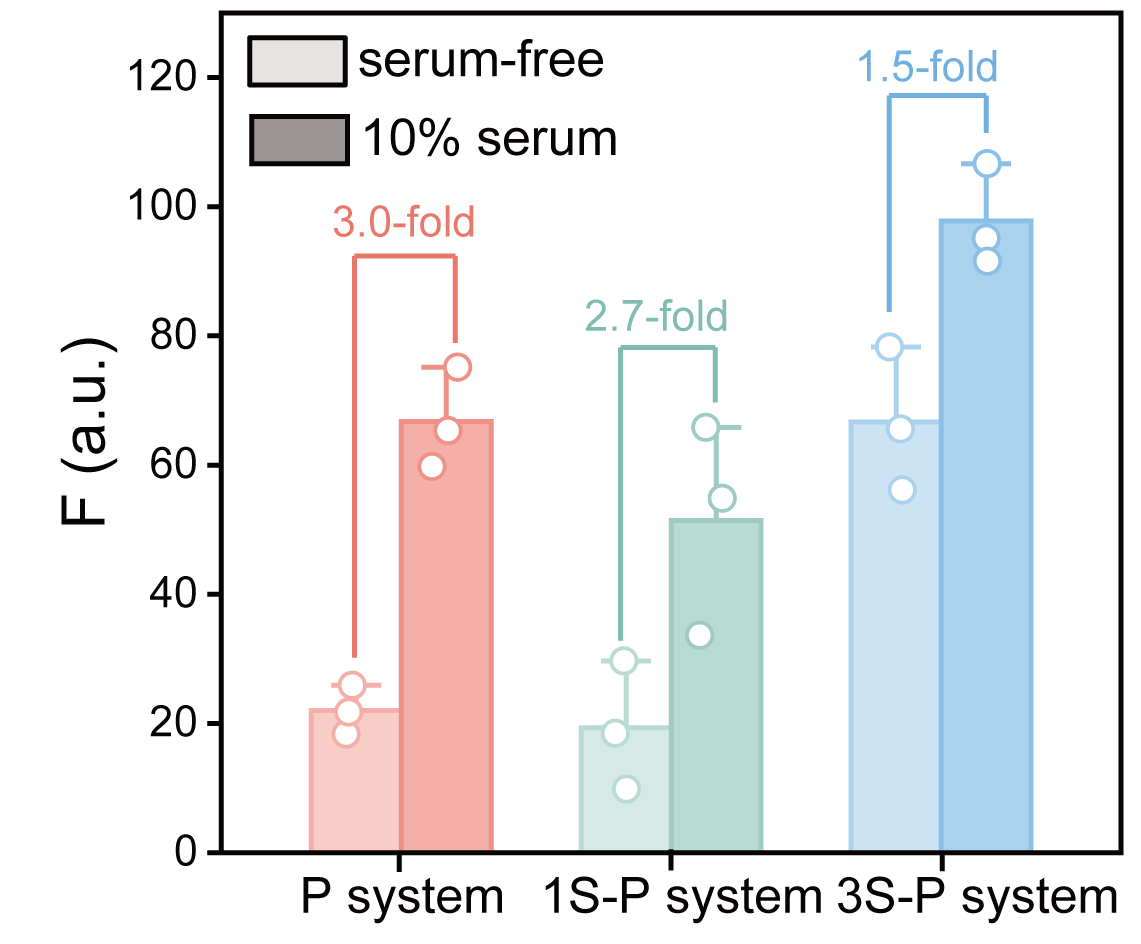


**Figure S9**. Serum stability comparison of P, 1S-P, and 3S-P probe systems. Error bars represent SD from three independent experiments.

As shown in **Figure S9**, in serum-free conditions, 3S-P exhibits a baseline fluorescence approximately threefold higher than P and 1S-P due to carrying three substrate units per probe. Upon exposure to 10% FBS, all probes show increased fluorescence as a result of nuclease-mediated cleavage; however, the magnitude of this increase differs markedly: P (3.0-fold), 1S-P (2.7-fold), and 3S-P (1.5-fold). The attenuated fluorescence rise of 3S-P indicates substantially enhanced resistance to serum nucleases, attributed to steric shielding and multivalent substrate organization within the streptavidin-based scaffold.


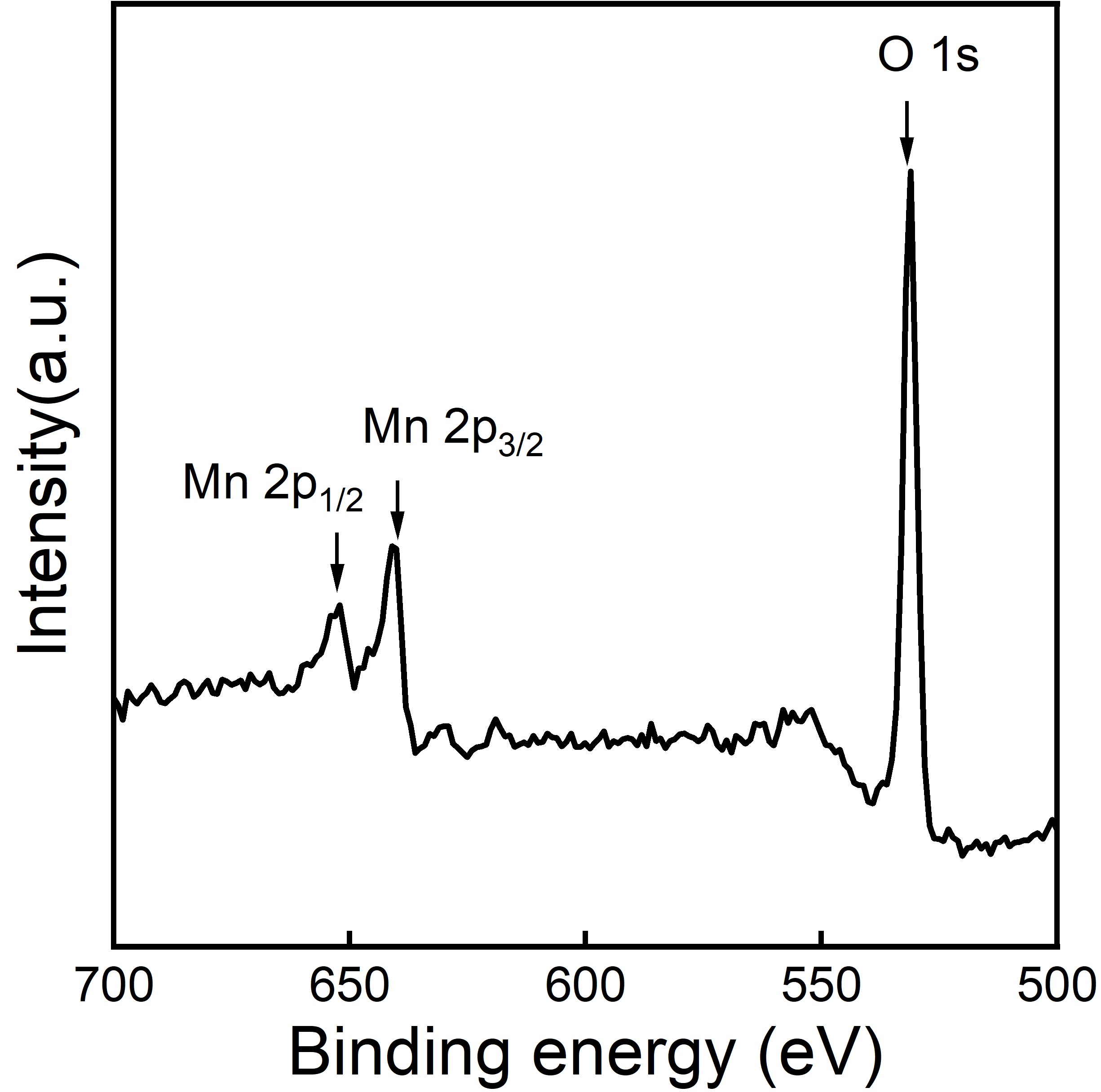


**Figure S10.** XPS spectra of MnO_2_ nanosheets.


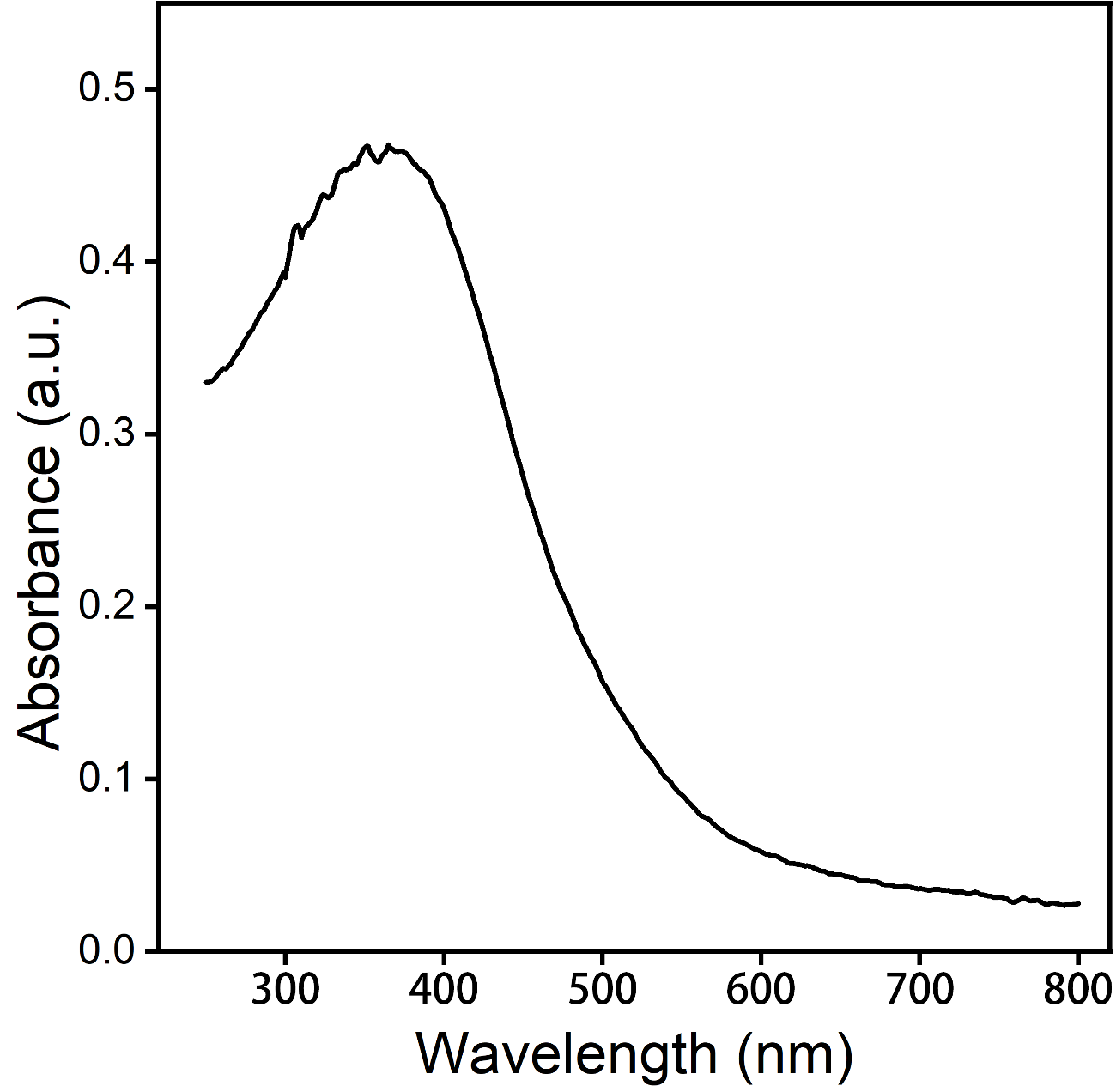


**Figure S11.** UV-vis absorption spectrum of MnO_2_ nanosheets.


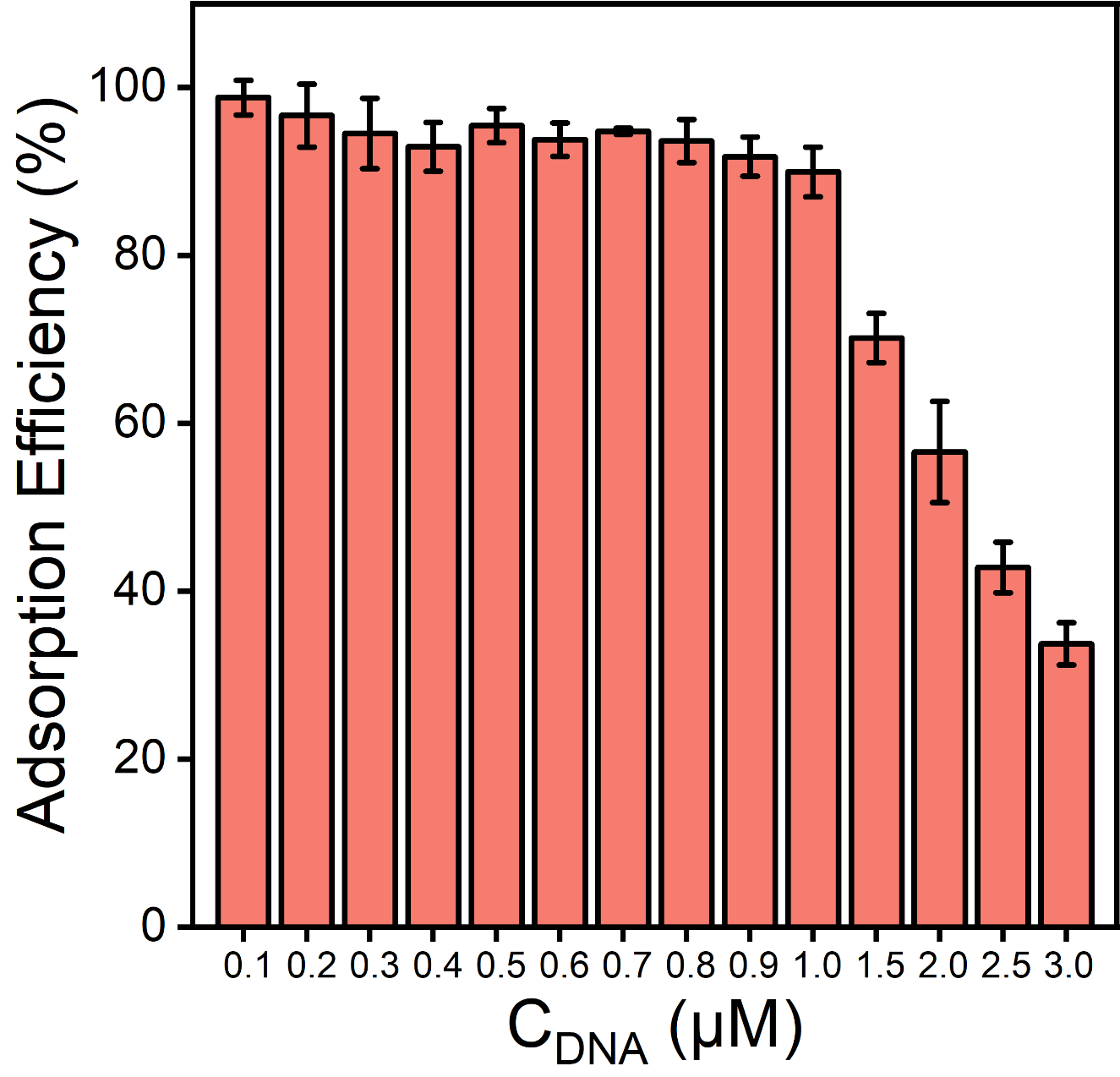


**Figure S12**. Adsorption efficiency of DNA probes onto MnO_2_ nanosheets.

Adsorption efficiency of nucleic acid probes on MnO_2_ nanosheets measured at different initial DNA concentrations (0.1-3.0 μM). DNA probes were incubated with MnO_2_ nanosheets (100 μg mL⁻¹) under the same conditions used for cellular delivery. After centrifugation, the unbound DNA remaining in the supernatant was quantified by UV-Vis absorbance at 260 nm, and adsorption efficiency was calculated by mass balance. As shown in **Figure S12**, more than 90% of the probes were adsorbed when the initial DNA concentration was ≤1.0 μM, whereas the efficiency gradually decreased at higher concentrations due to saturation of available surface sites. These results indicate a loading capacity of approximately 10 pmol DNA per μg MnO_2_, confirming that the probe concentration used in our cellular experiments (200 nM) is well below the saturation threshold, ensuring efficient and stable adsorption.


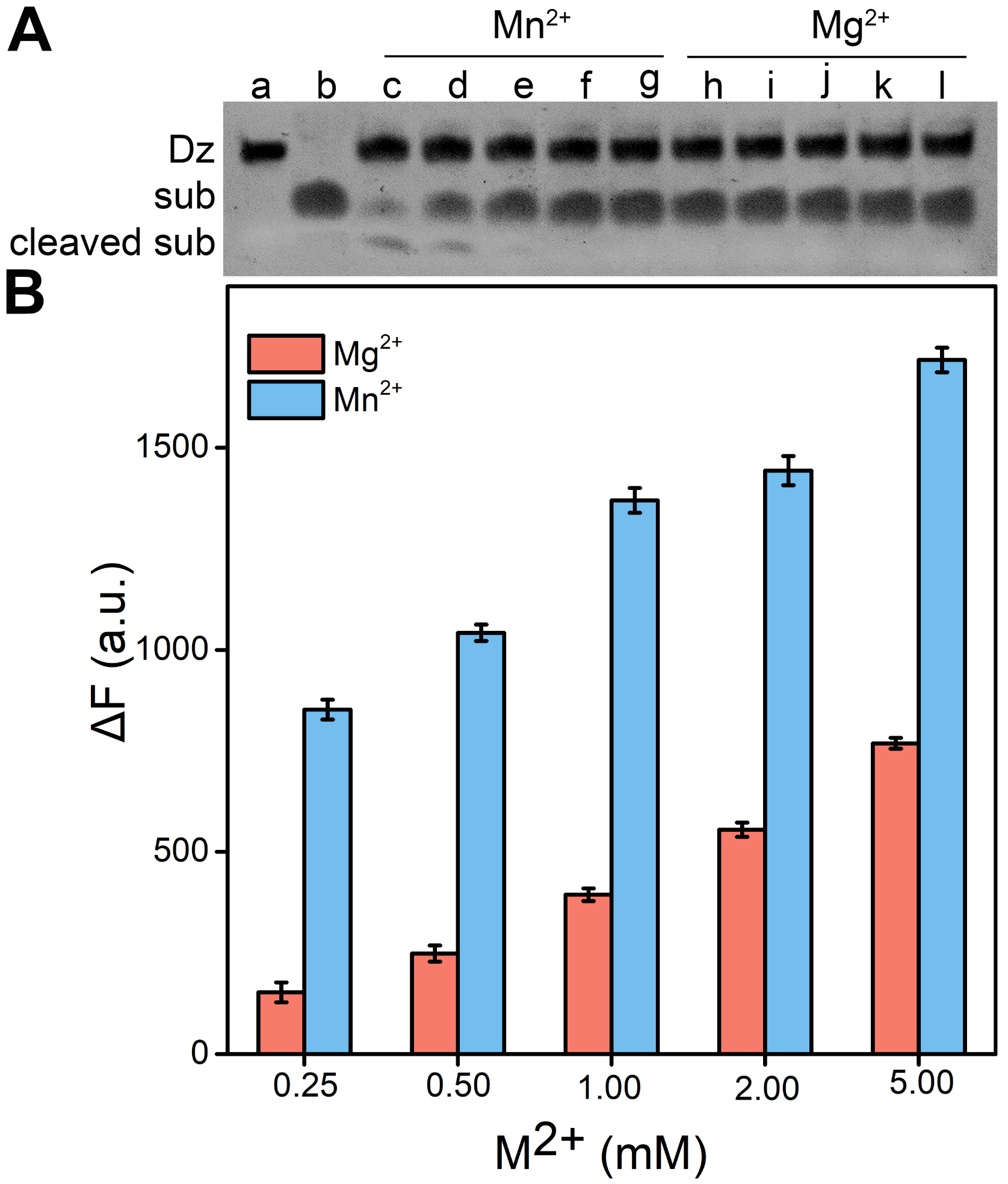


**Figure S13**. M^2+^-dependent DNAzyme biocatalysis study. (A) Gel electrophoresis analysis of DNAzyme catalytic efficiency with Mn^2+^: 5 mM (lane c), 2mM (lane d), 1 mM (lane e), 0.5 mM (lane f), 0.25 mM (lane g) and Mg^2+^:5 mM (lane h), 2 mM (lane i), 1 mM (lane j), 0.5 mM (lane k), 0.25 mM (lane l). (B) Change in fluorescence intensity of the DNAzyme cleavage reaction in the presence of different concentrations of Mn^2+^ or Mg^2+^. Error bars represent the standard deviation (SD) from three independent experiments.


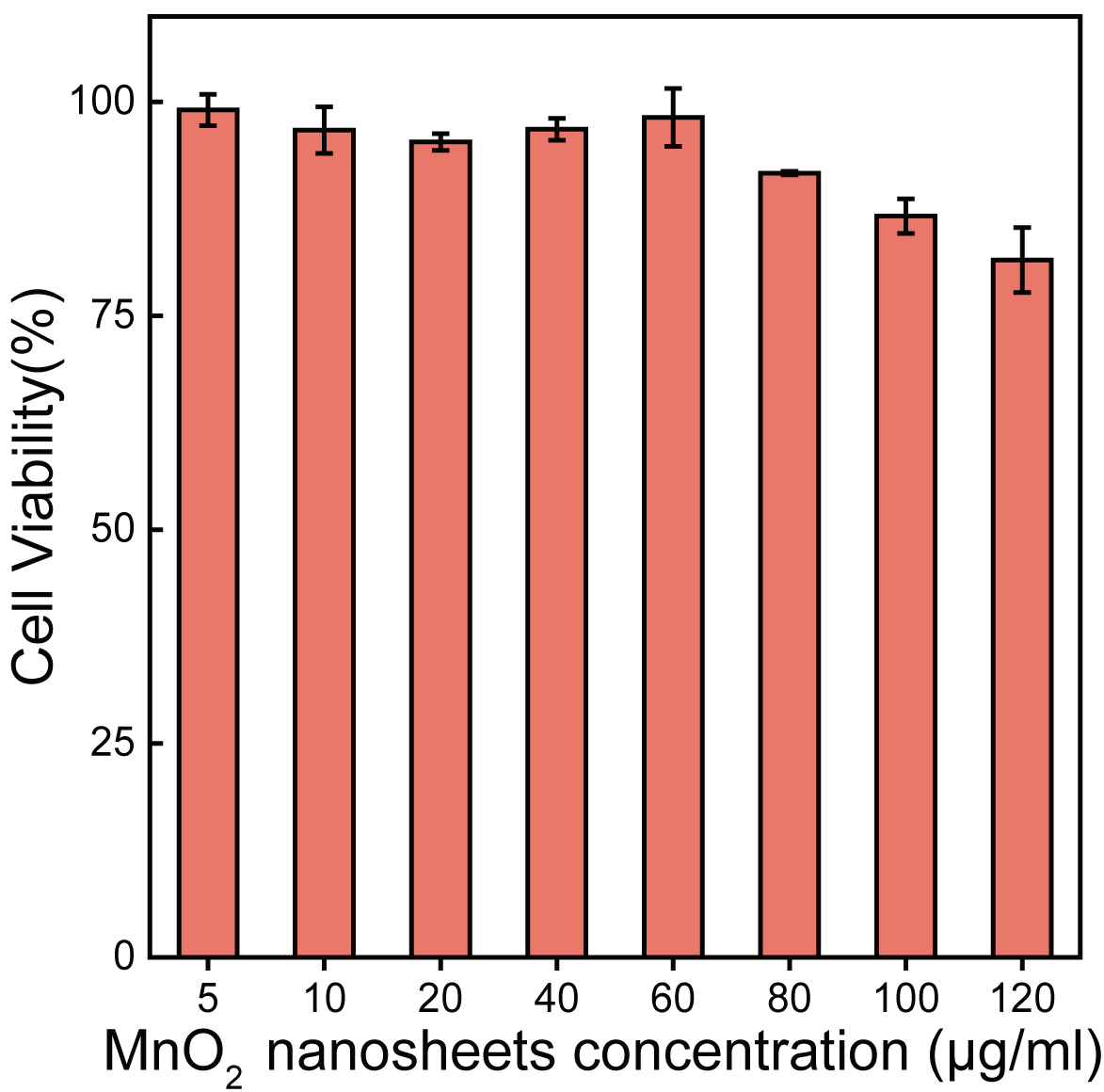


**Figure S14.** Cell proliferation assay to investigate the cytotoxicity of MnO_2_ nanosheets. Cell viability values (%) were determined by incubating MCF-7 cells with MnO_2_ nanosheets of varied concentrations (5**-**120 µg/mL) for 24 h. Error bars represent the standard deviation (SD) from three independent experiments.


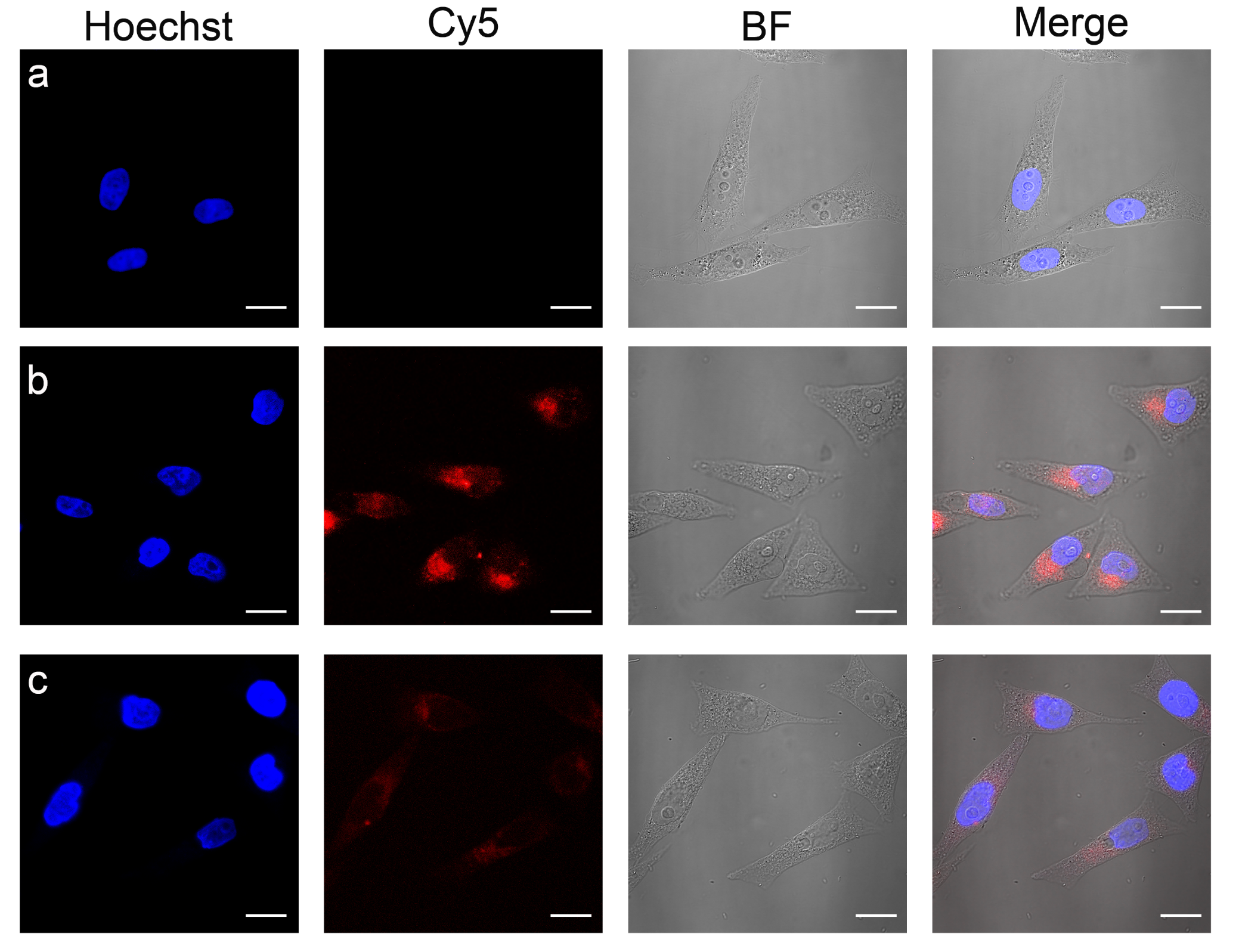


**Figure S15.** Confocal laser scanning microscopy (CLSM) images of MCF-7 cells incubated with the bare P system, a: substrate, b: P_0_+P_1_+substrate, c: P_0_+P_2_+substrate.


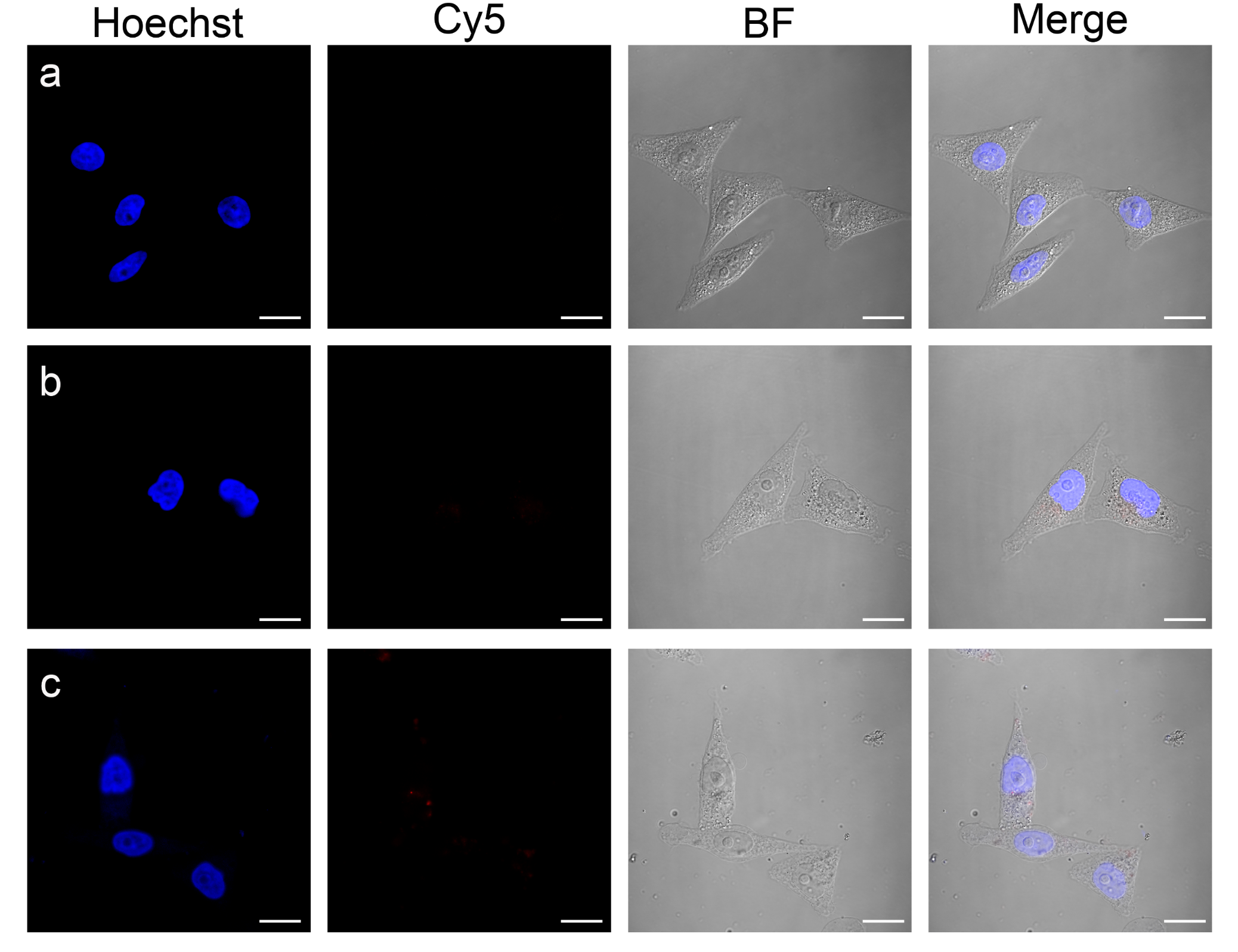


**Figure S16.** CLSM images of MCF-7 cells incubated with the mutant partzymes, a: substrate, b: Mutant-P_0_+Mutant-P_1_+substrate, c: Mutant-P_0_+Mutant-P_2_+substrate. Scale bars: 20 μm.


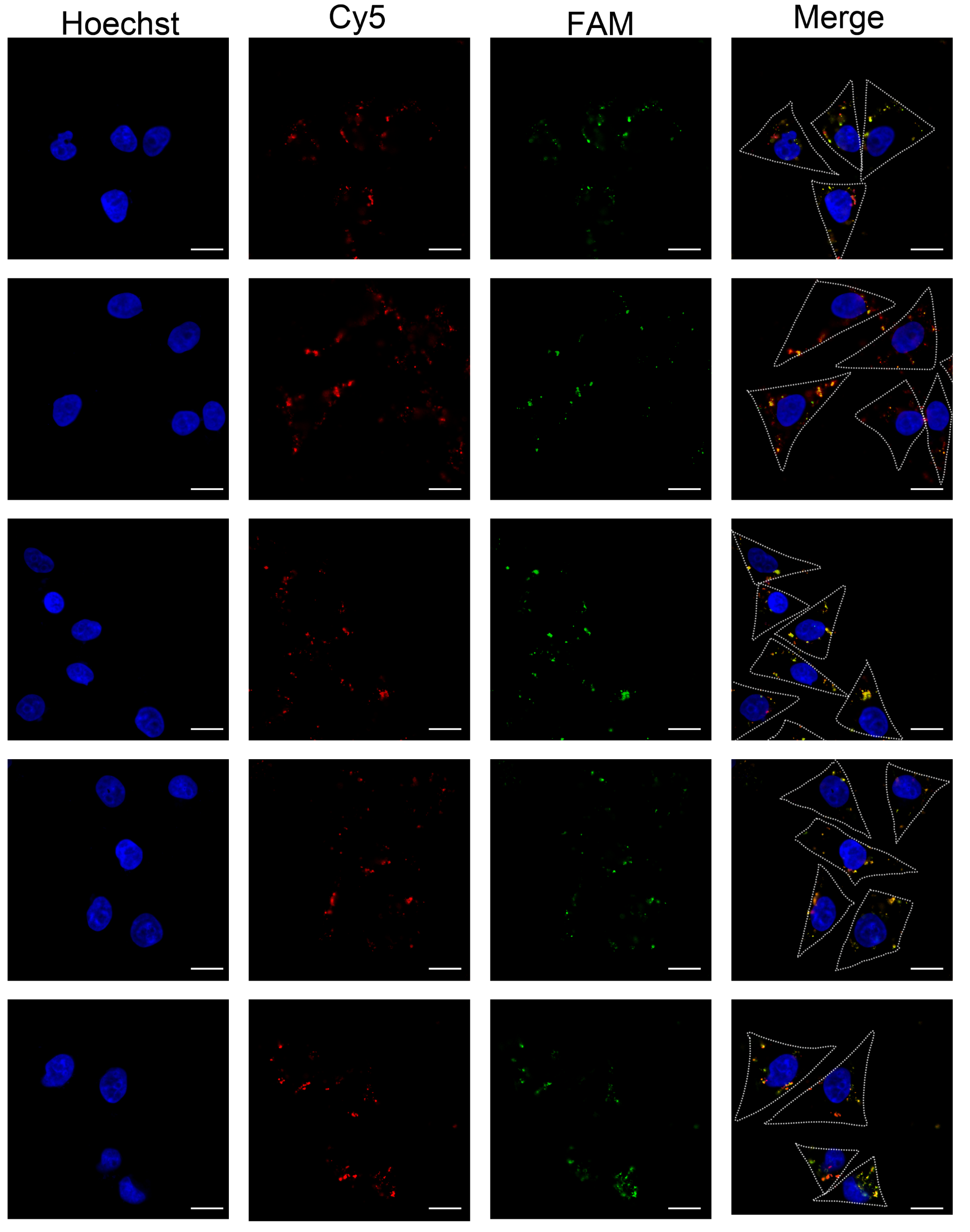


**Figure S17.** CLSM images of MCF-7 cells incubated with 1S-P and 3S-P system. (A) Images of cells incubated with the 1S-P system. Scale bars: 20 μm.


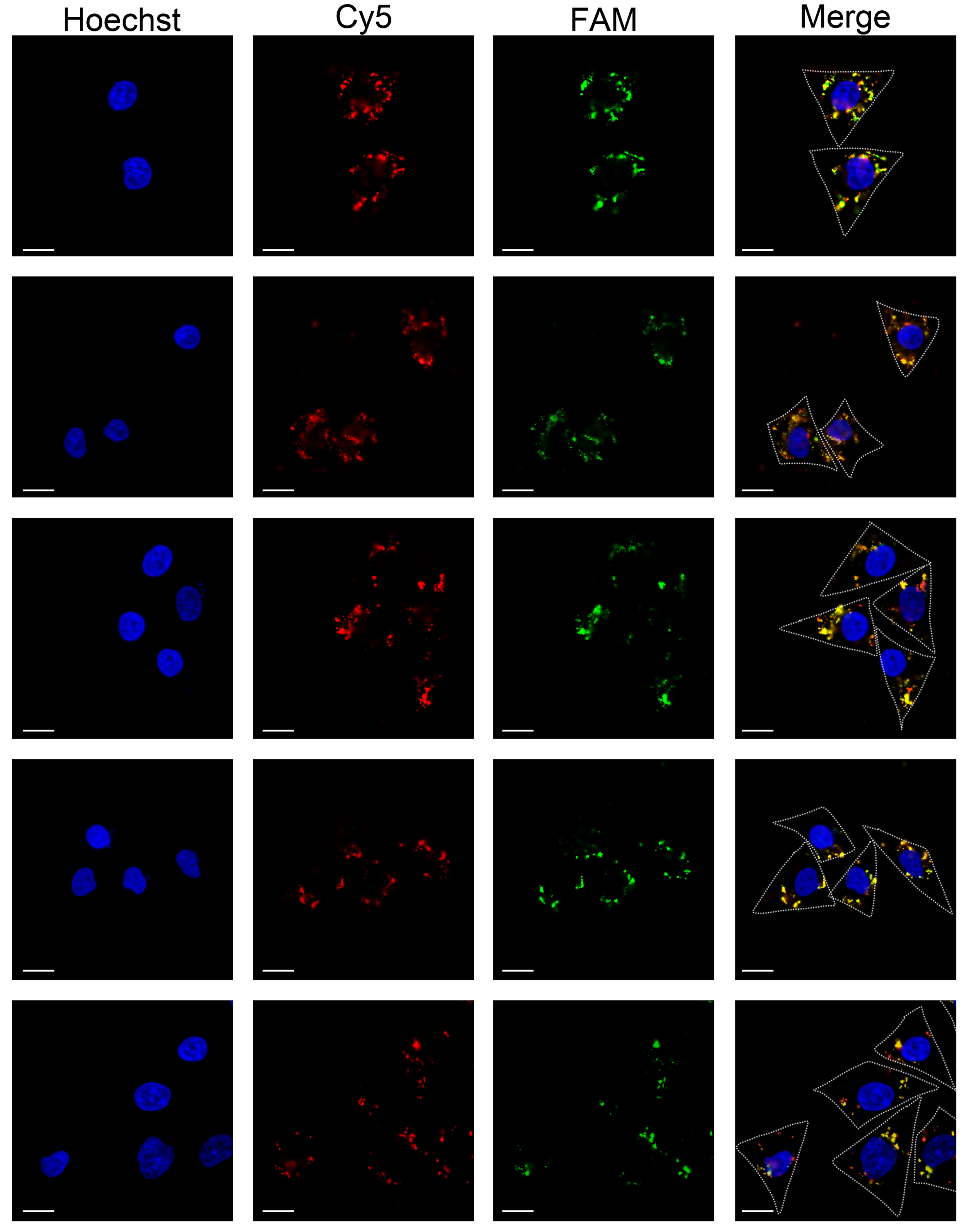


**Figure S17.** (B) Images of cells incubated with the 3S-P system. Scale bars: 20 μm.


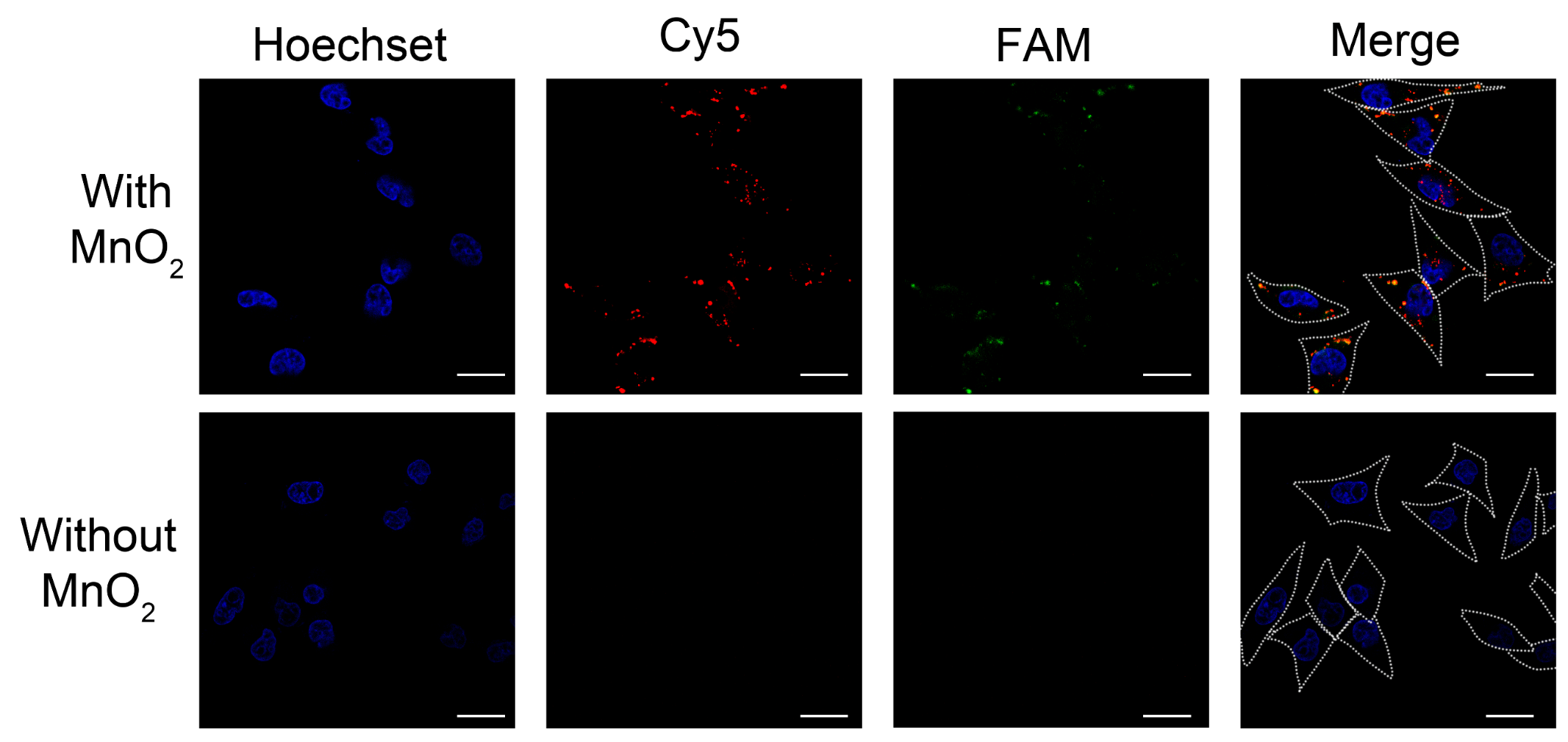


**Figure S18**. CLSM images of cells treated with the SUPER probes in the presence or absence of MnO_2_ nanosheets.


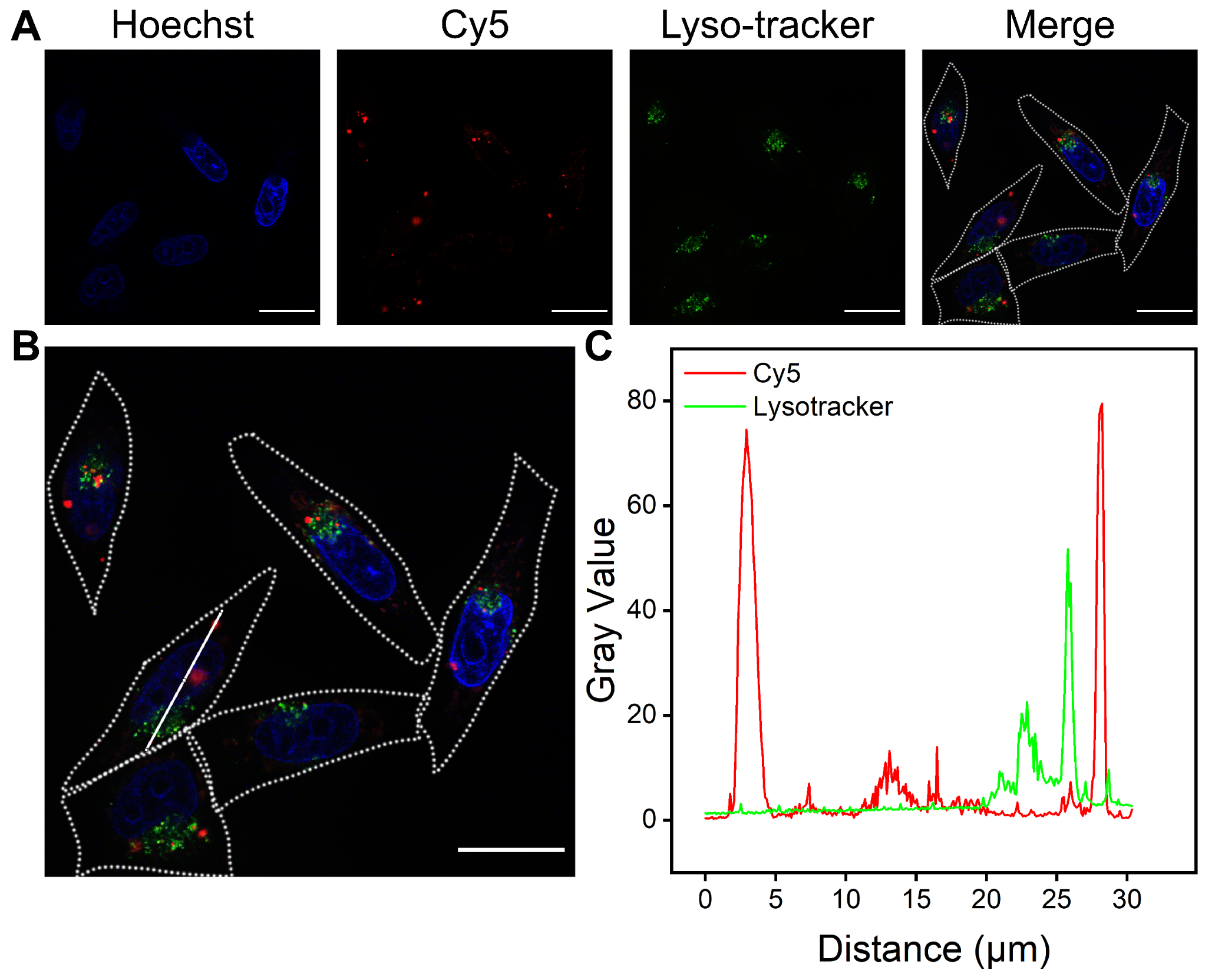


**Figure S19**. CLSM images of SUPER probes and Lysotracker at 4 h post-transfection. (A) CLSM images of MCF-7 cells treated with SUPER probes for 4 h. (B) CLSM image highlighting the spatial distribution of Cy5 and Lysotracker signals within representative cells.

(C) Fluorescence line-scan analysis along the indicated white line in (B), showing the intensity profiles of Cy5 (red) and Lysotracker (green) signals. Scale bars: 20 μm.


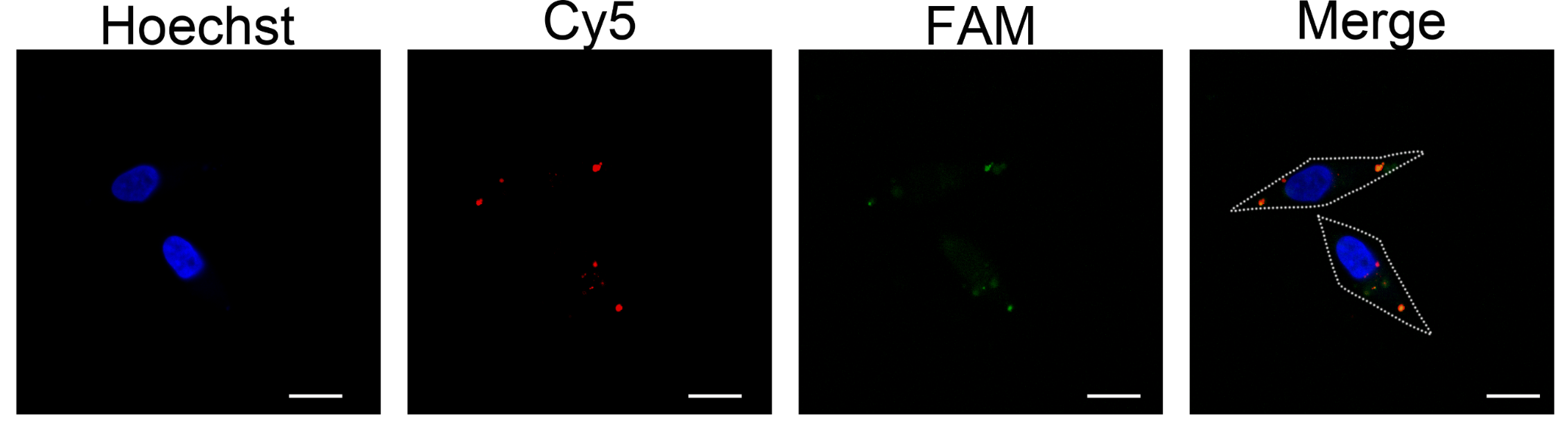


**Figure S20**. CLSM images of MCF-7 cells that were pre-transfected with Anti-P_0_ and Anti-P_1_, and incubated with the 3S-P system. Scale bars: 20 μm.


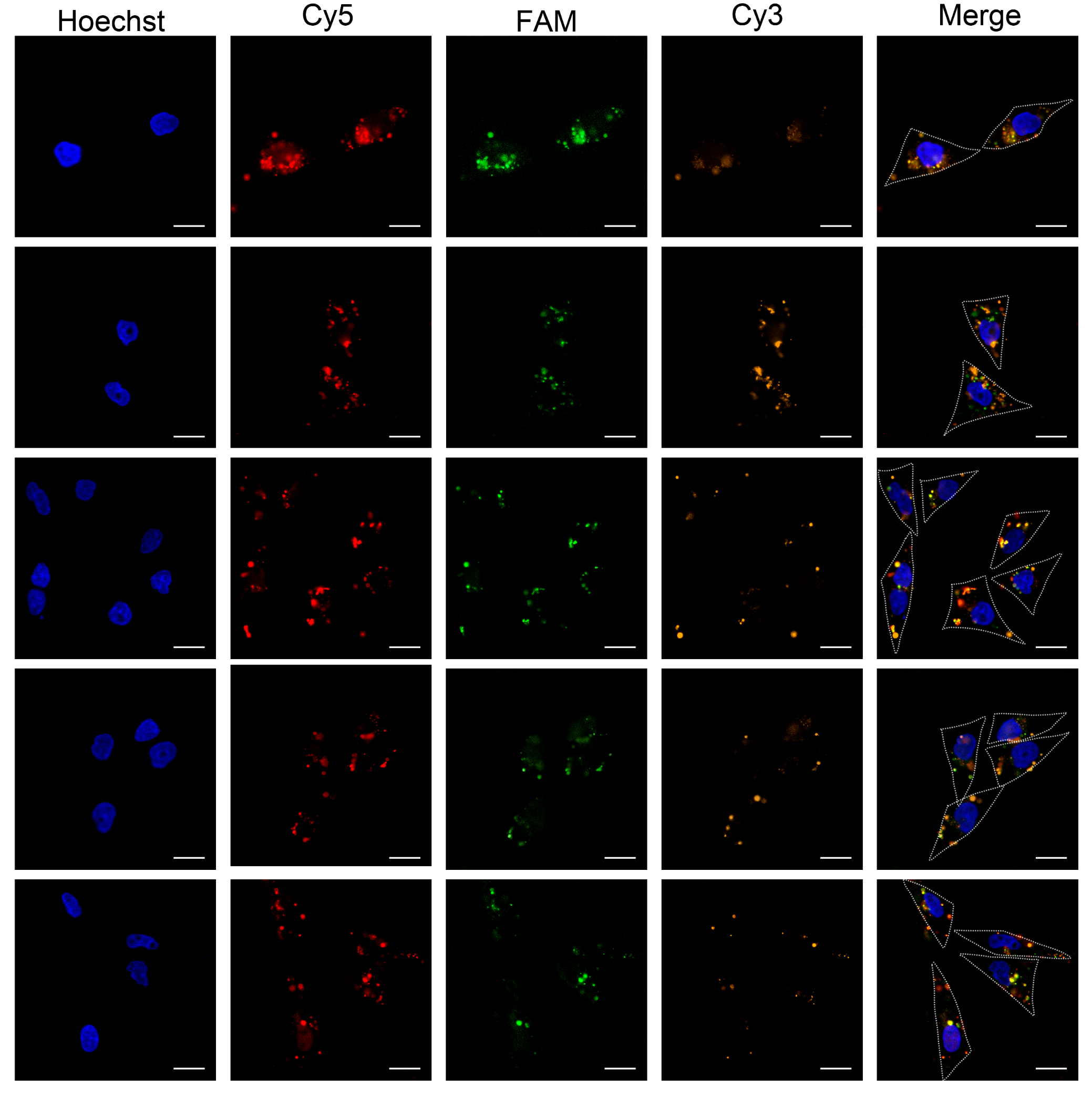


**Figure S21**. CLSM imaging of representative MCF-7 cells incubated with the 3S-P system under various pretreatments (no pretreatment, Bcl-xL mimics, or oxaliplatin) (A) Images of cells with no pretreatment. Scale bars: 20 μm.


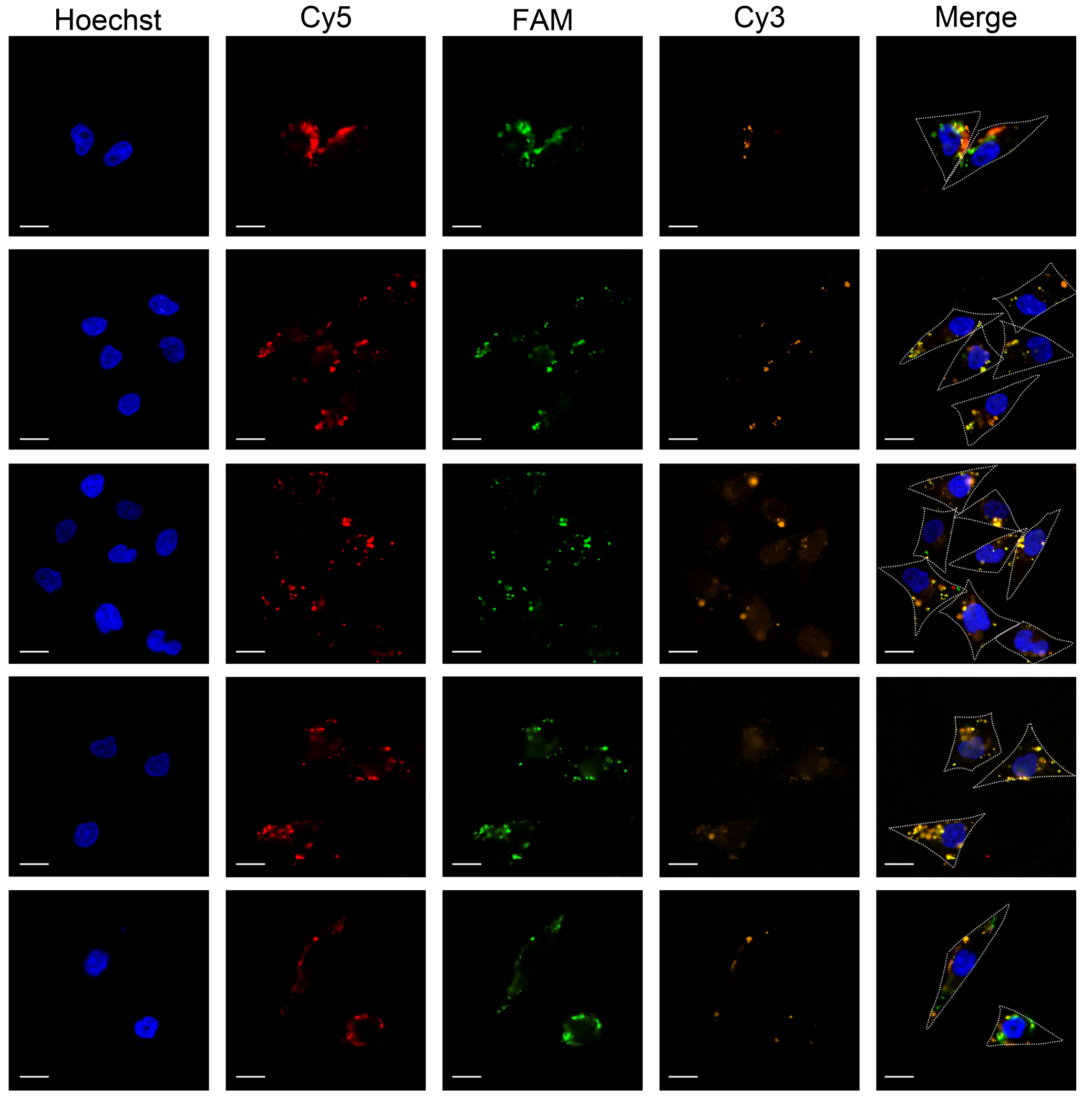


**Figure S21.** (B) Images of Bcl-xL-mimics-pretreated cells. Scale bars: 20 μm.


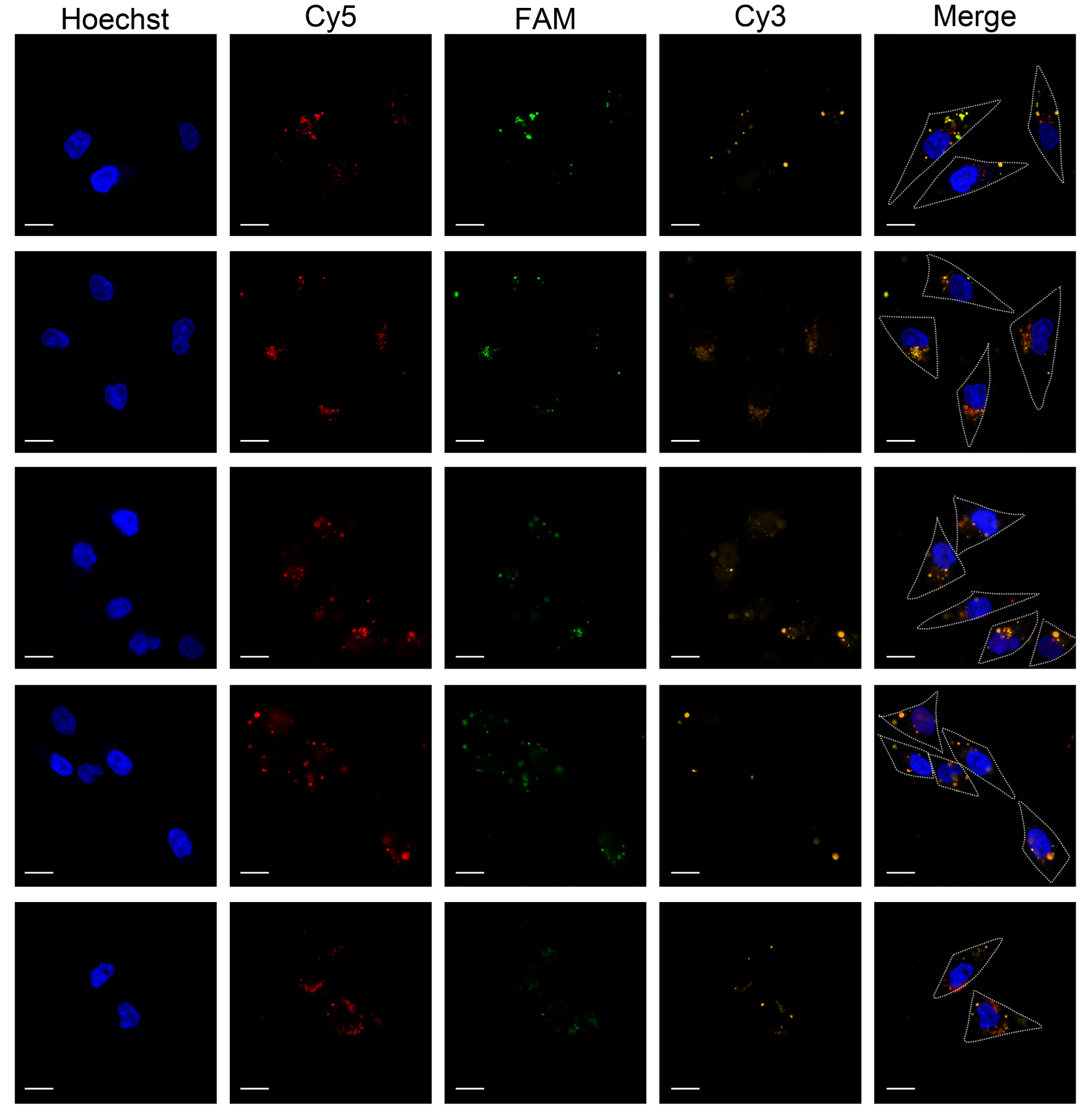


**Figure S21.** (C) Images of oxaliplatin-pretreated cells. Scale bars: 20 μm.


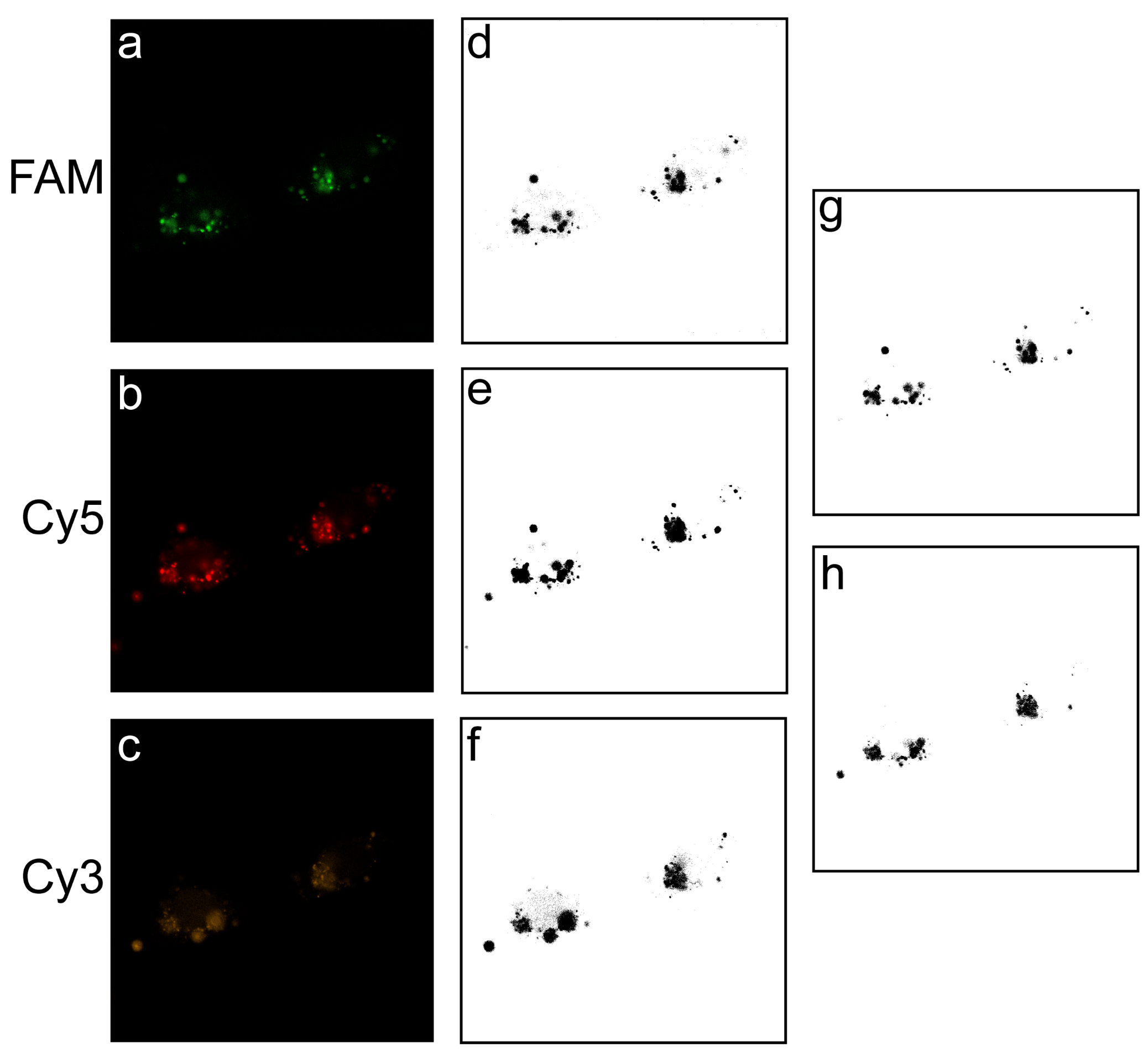


**Figure S22.** Scheme of the protocol for image analysis. ImageJ software was used for quantification of fluorescence images. The original images obtained from the confocal microscope (FAM, Cy5, and Cy3 channels) were denoted as a, b, and c respectively, and their corresponding 8-bit mode conversions were represented by d, e, and f. To exclude nonspecific fluorescence, images containing double-labeled spots (g: Cy5 AND FAM, h: Cy5 AND Cy3) were obtained from the 8-bit mode images using “AND” operation in Image Calculator of ImageJ. Fluorescence intensity of Cy3 and FAM signals was quantified. R0 (F_FAM_ /F_Cy3_ before background noise subtraction) was calculated as F_FAM_ d/F_Cy3_ f while RS (F_FAM_/F_Cy3_ after background noise subtraction) was calculated as F_FAM_ g/F_Cy3_ h.

**
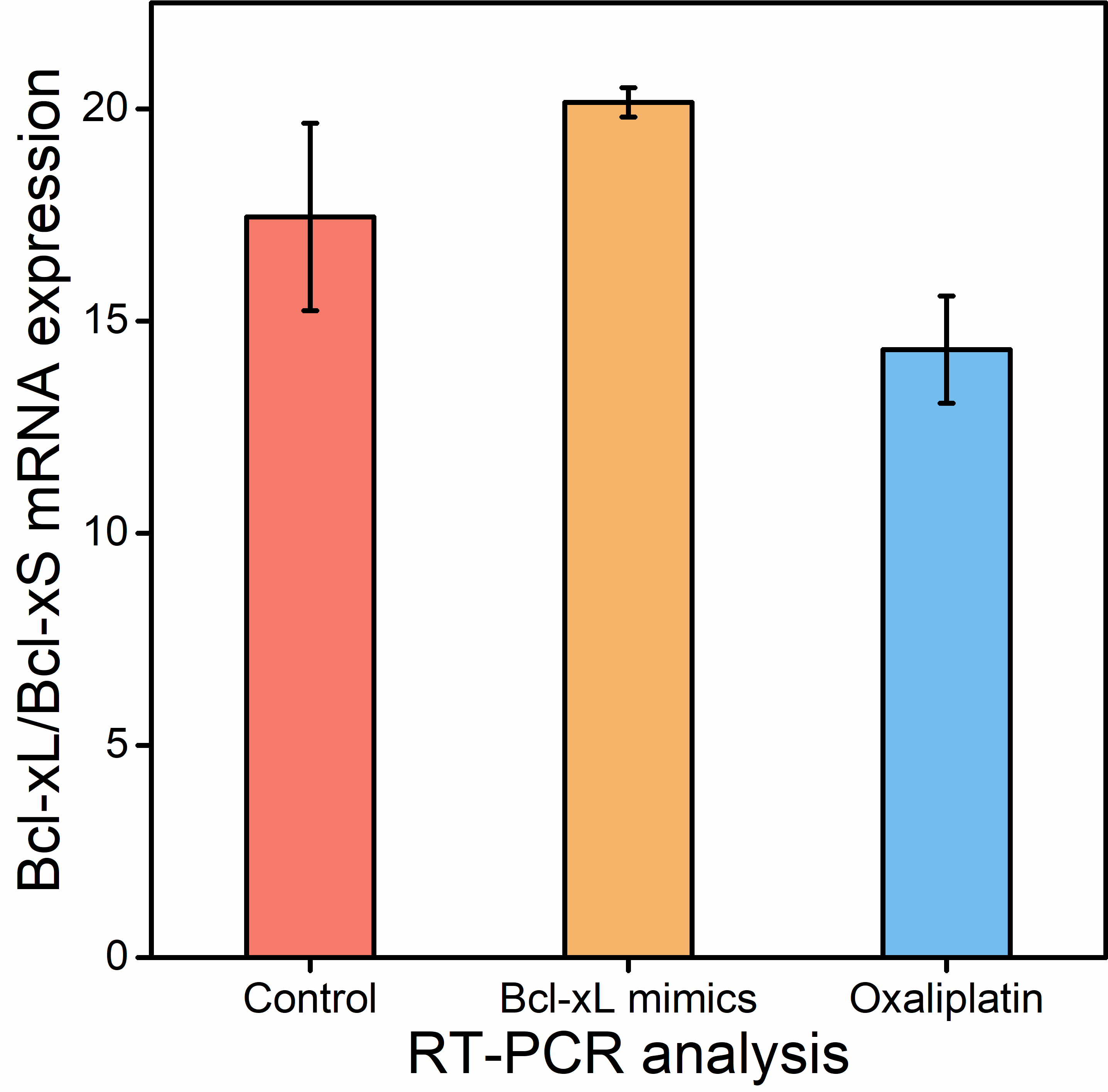
**

**Figure S23.** Real-time quantitative PCR (RT-qPCR) analysis of Bcl-xL/Bcl-xS mRNA expression in MCF-7 cells under different treatment conditions. Error bars represent the standard deviation (SD) from three independent experiments.


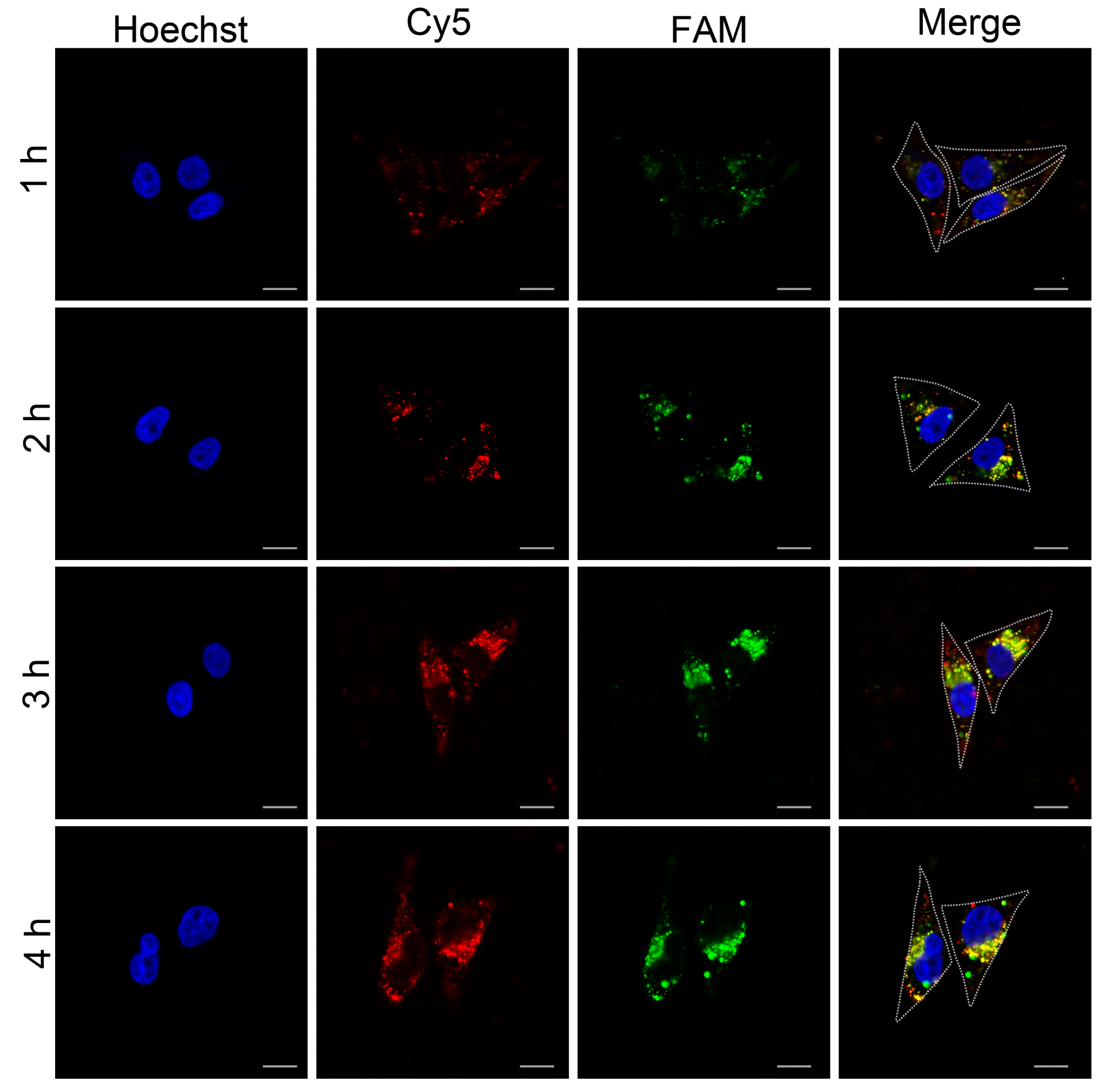


**Figure S24.** Time-dependent CLSM imaging of MCF-7 cells treated with 3S-P system at different time intervals (1-4 h). (A) Representative images showing MCF-7 cells at different time intervals (1 h, 2 h, 3 h and 4 h).


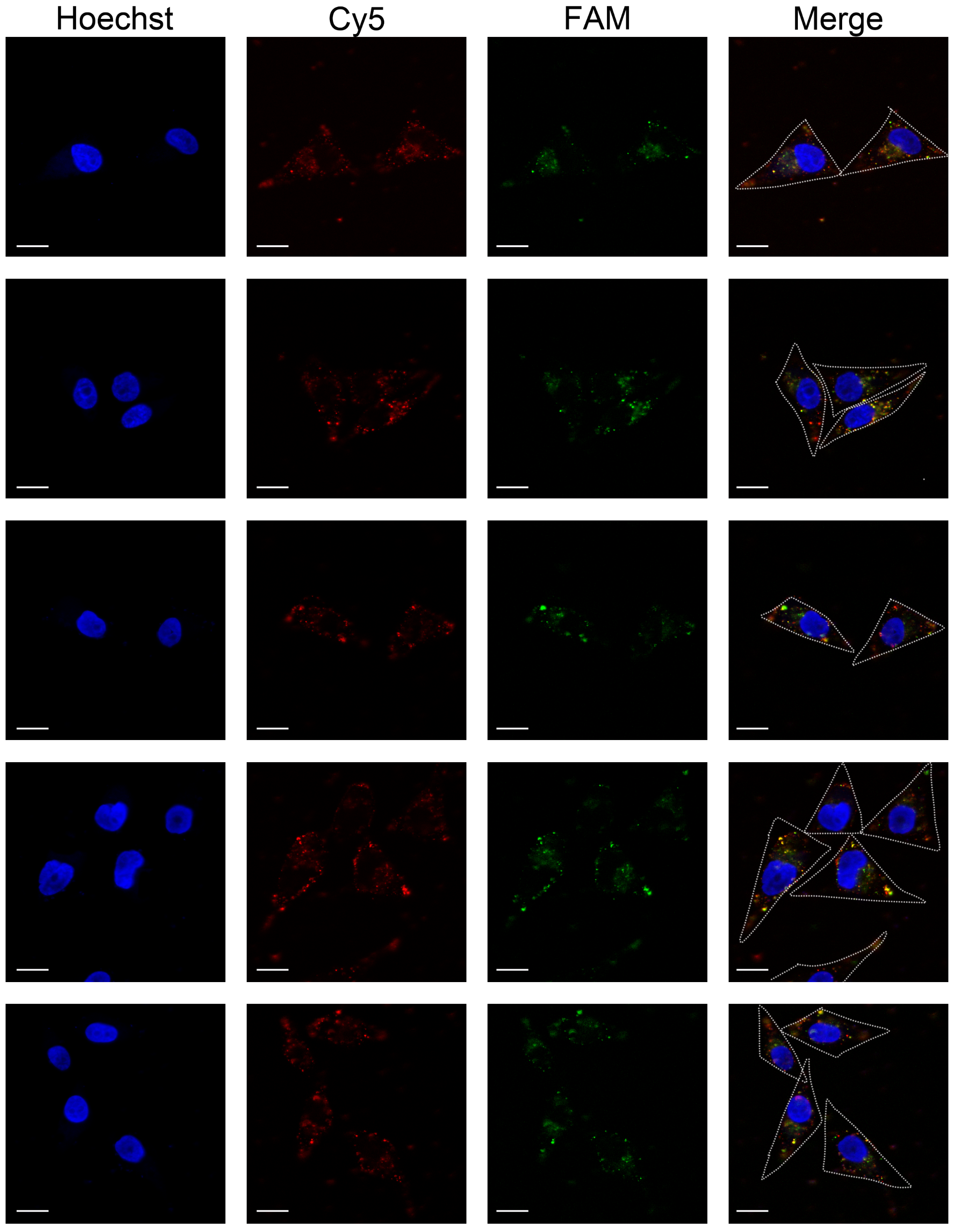


**Figure S24.** (B) Images of cells incubated with 3S-P system for 1 h. Scale bar: 20 μm.


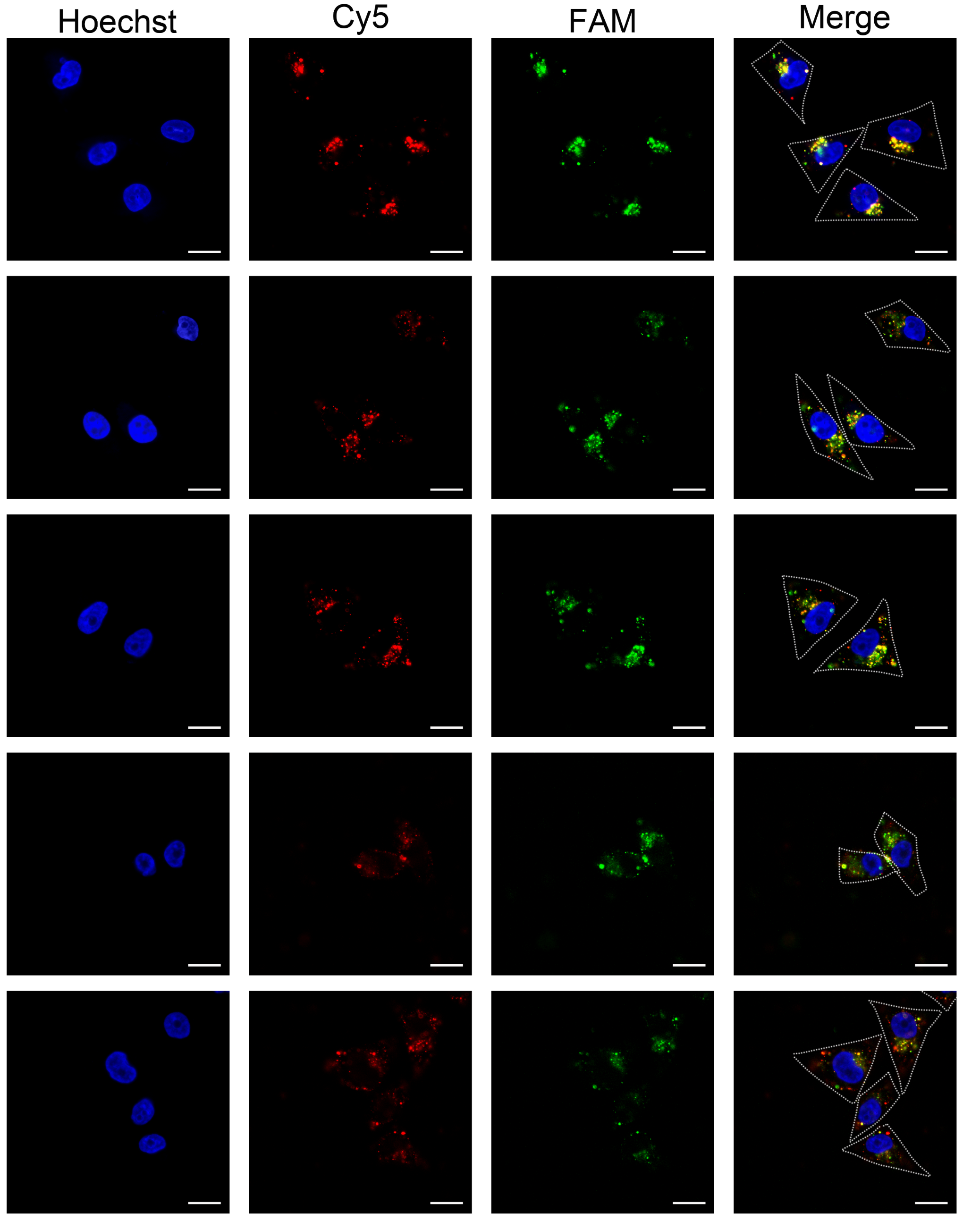


**Figure S24.** (C) Images of cells incubated with 3S-P system for 2 h. Scale bar: 20 μm.


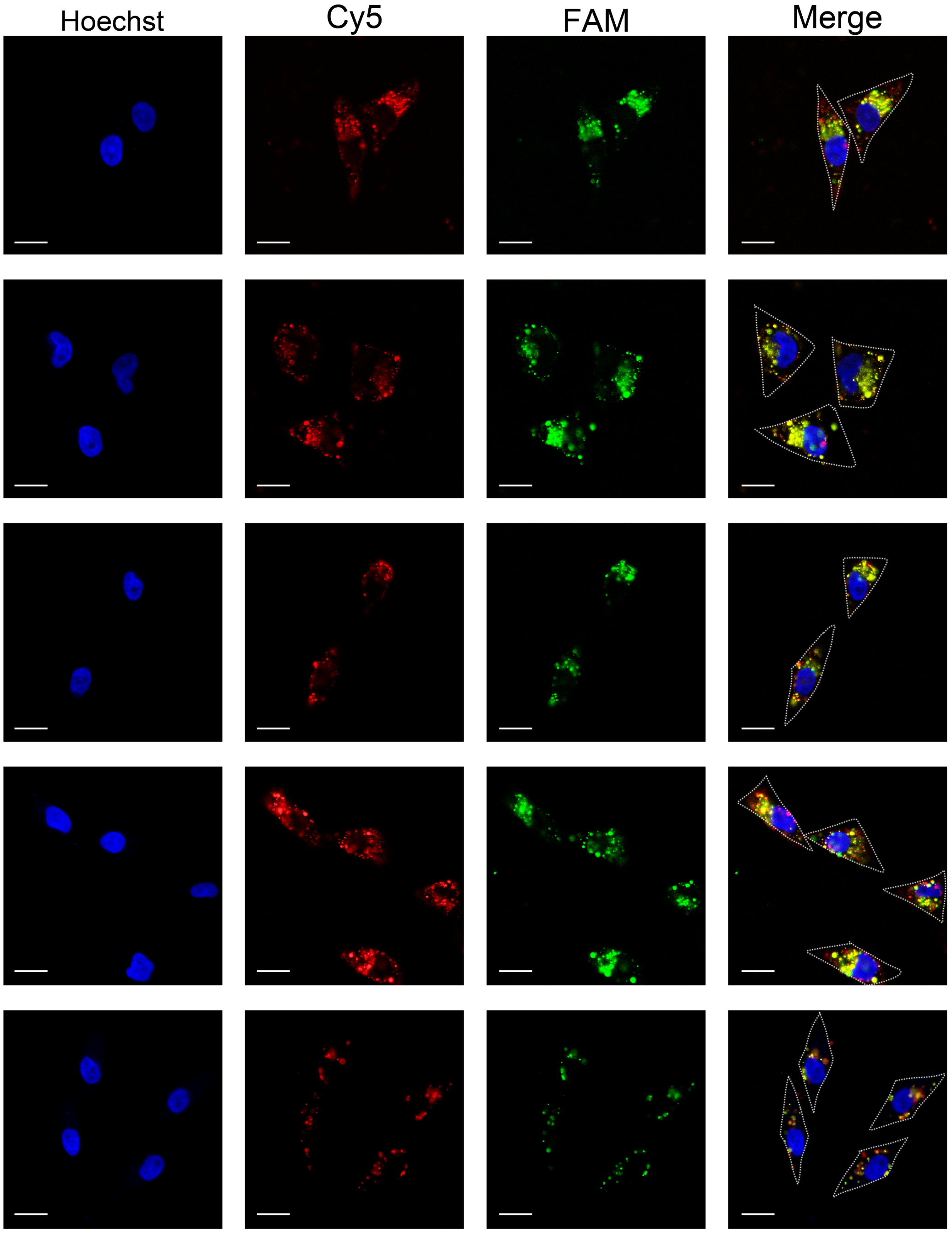


**Figure S24.** (D) Images of cells incubated with 3S-P system for 3 h. Scale bar: 20 μm.


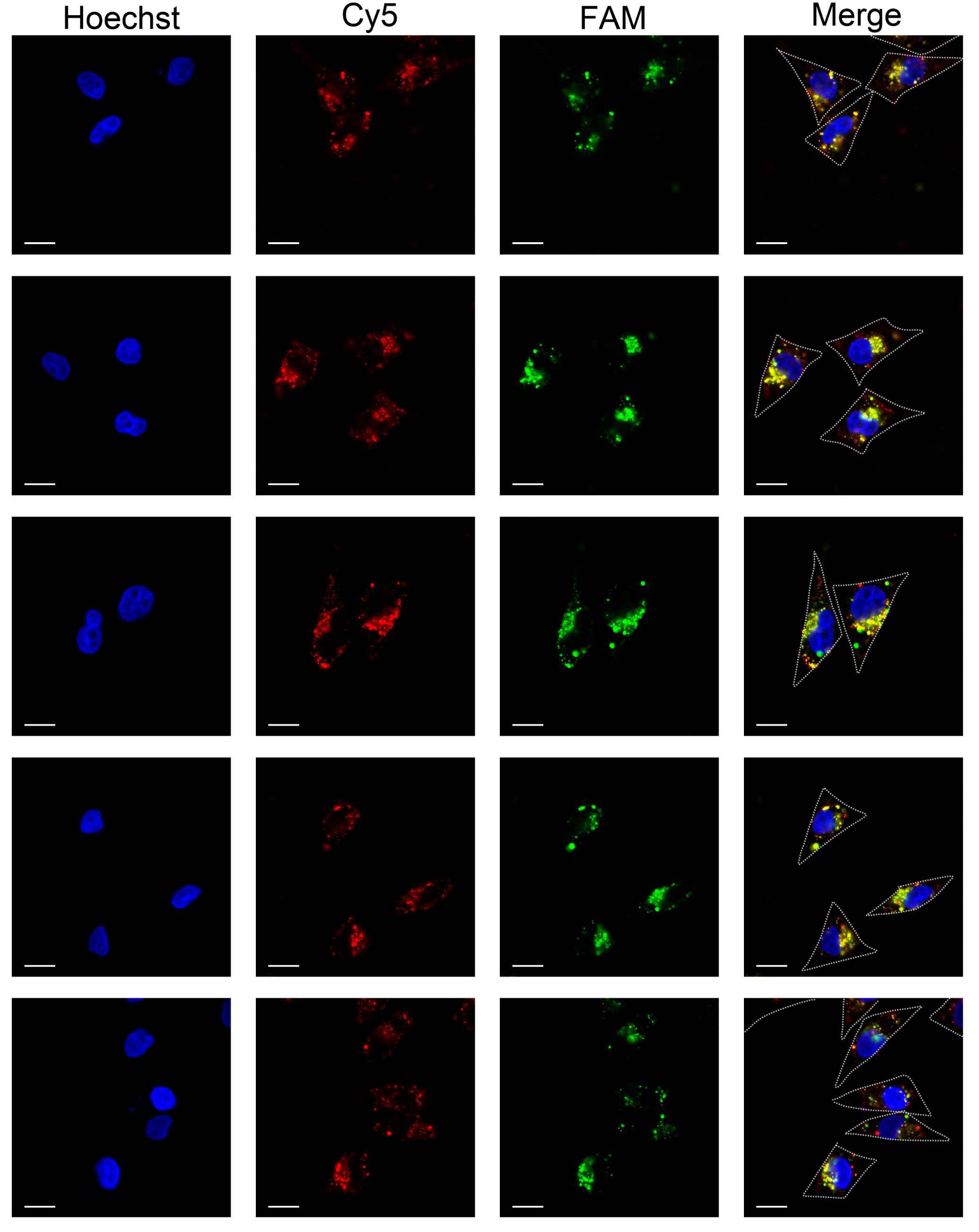


**Figure S24.** (E) Images of cells incubated with 3S-P system for 4 h. Scale bar: 20 μm.


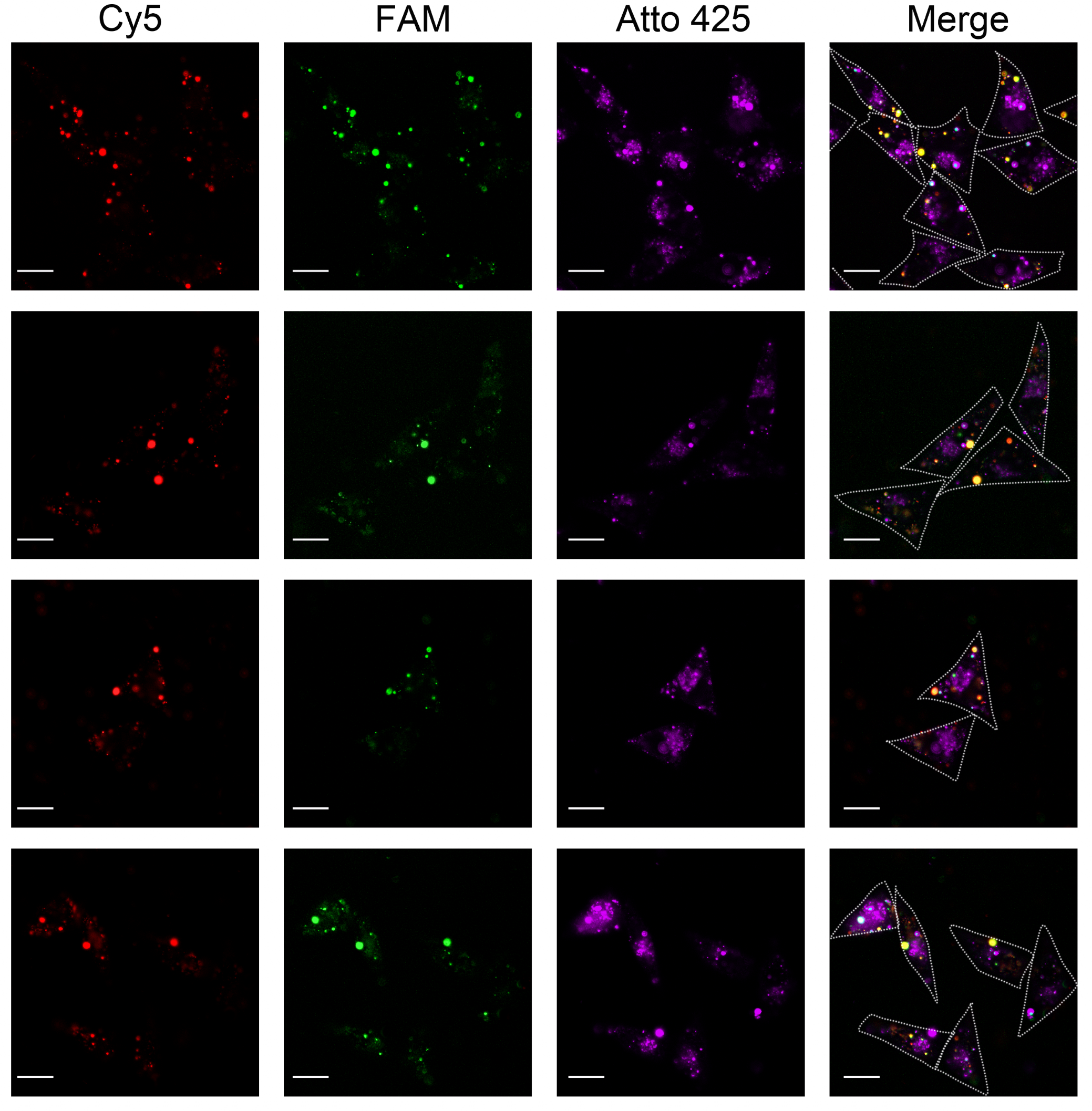


**Figure S25.** Time-dependent CLSM images of MCF-7 cells that were pre-transfected with AS1411 and treated with the 3S-P system at different time intervals (1-4 h). (A) Images at 1 h. Scale bar: 20 μm.


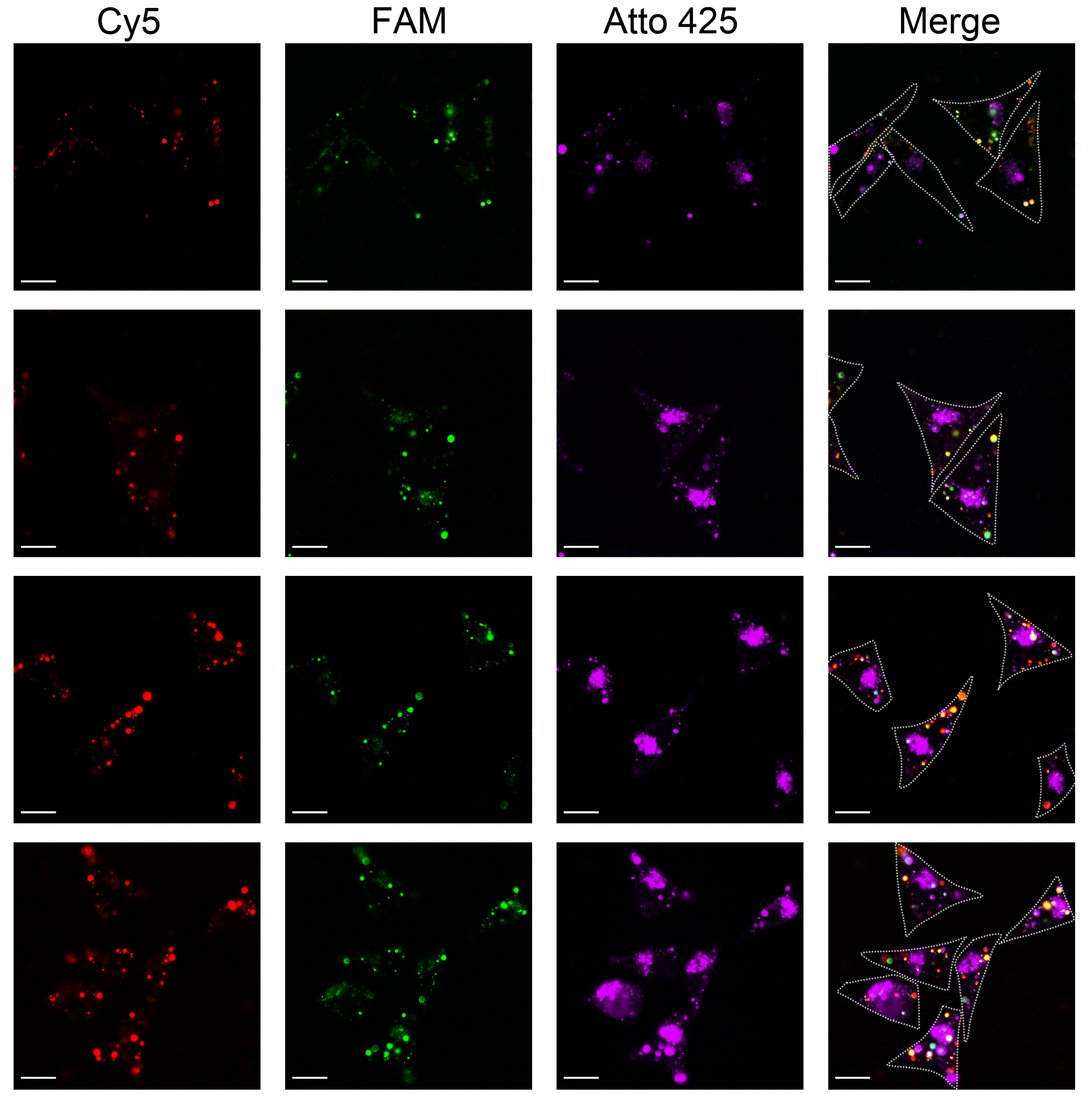


**Figure S25.** (B) Images at 2 h. Scale bar: 20 μm.


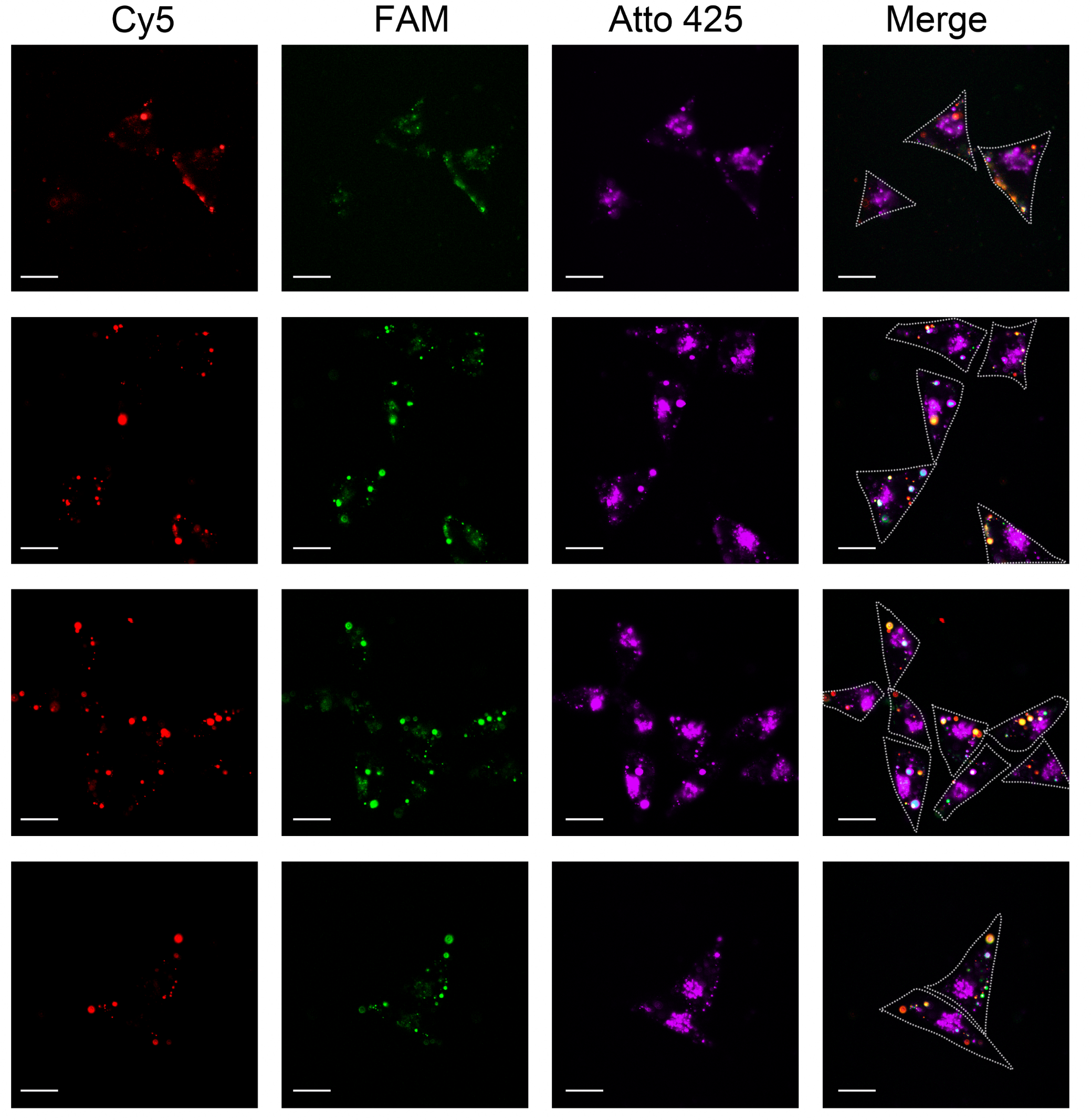


**Figure S25.** (C) Images at 3 h. Scale bar: 20 μm.


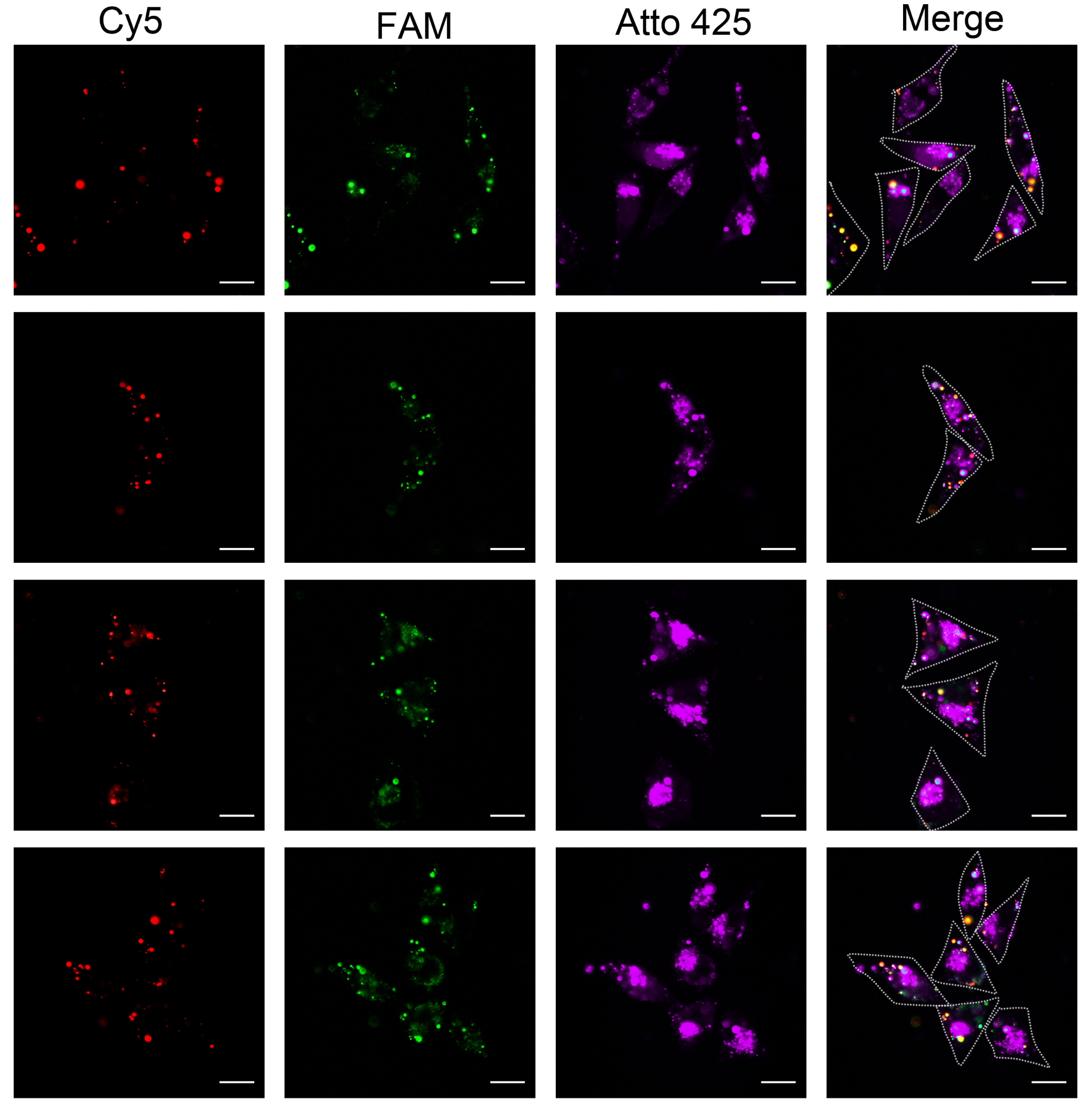


**Figure S25.** (D) Images at 4 h. Scale bar: 20 μm.


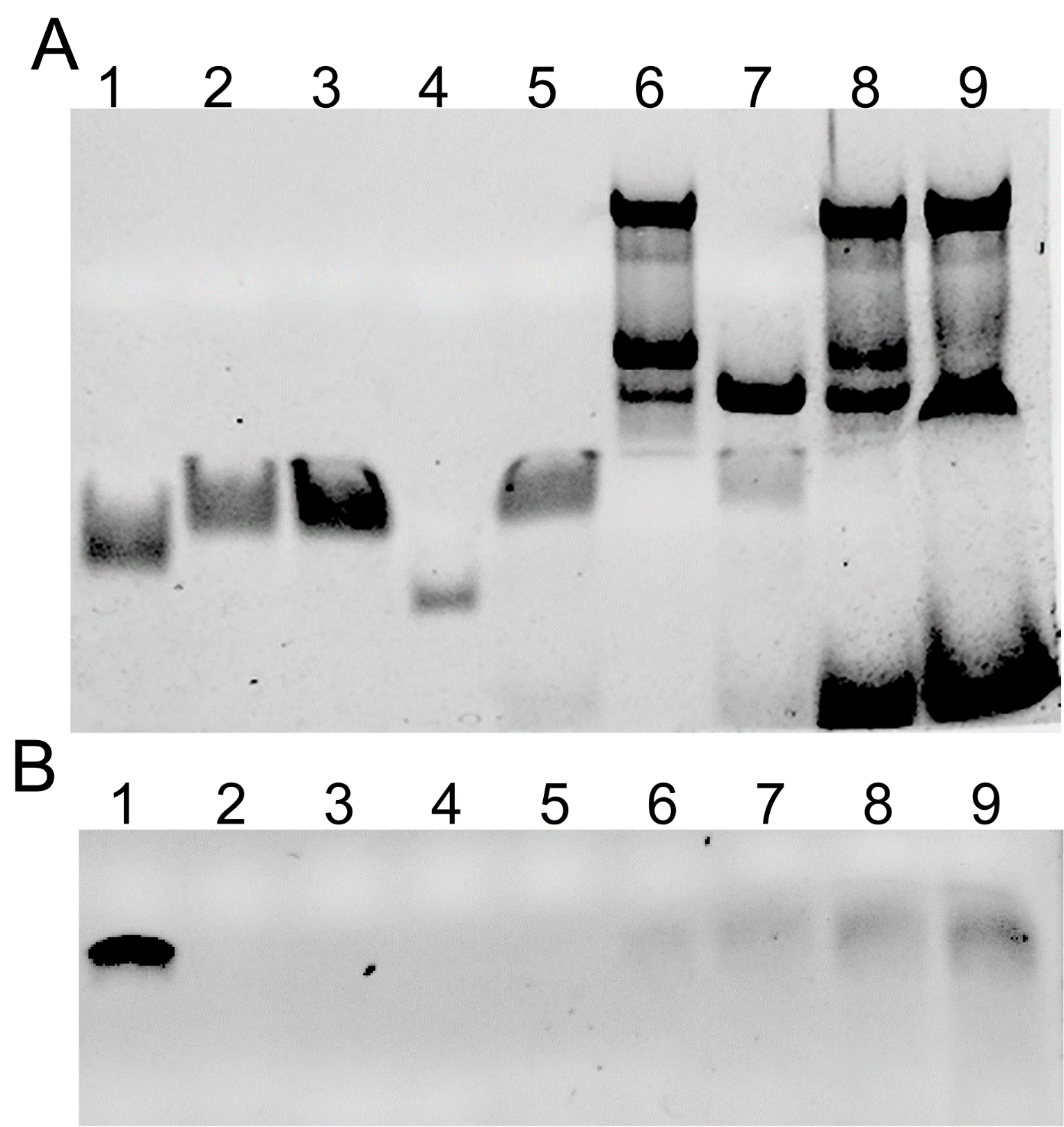


**Figure S26.** PAGE analysis of the blocker cleavage reaction. Lane 1: P_0_, lane 2: P_1_, lane 3: Bcl-xL, lane 4: AS1411, lane 5: blocker, lane 6: P_0_+P_1_+Bcl-xL, lane 7: AS1411+blocker, lane 8: P_0_+P_1_+Bcl-xL+blocker, lane 9: P_0_+P_1_+Bcl-xL+AS1411+blocker. (B) GelRed staining comparison between linear DNA and AS1411 across a concentration gradient. Lane 1: linear 26 bp DNA (100 nM), lane 2: AS1411 (100 nM), lane 3: AS1411 (200 nM), lane 4: AS1411 (400 nM), lane 5: AS1411 (800 nM), lane 6: AS1411 (1 μM), lane 7: AS1411 (2 μM), lane 8 AS1411 (5 μM), lane 9: AS1411 (10 μM). Linear DNA is readily visualized at ~100 nM, whereas AS1411 requires concentrations ≥1 μM to produce detectable bands, confirming the inherently weak GelRed response of G-quadruplex oligonucleotides. This explains the low-intensity AS1411 band observed in lane 9 of panel A, where the liberated AS1411 concentration was approximately 200 nM.


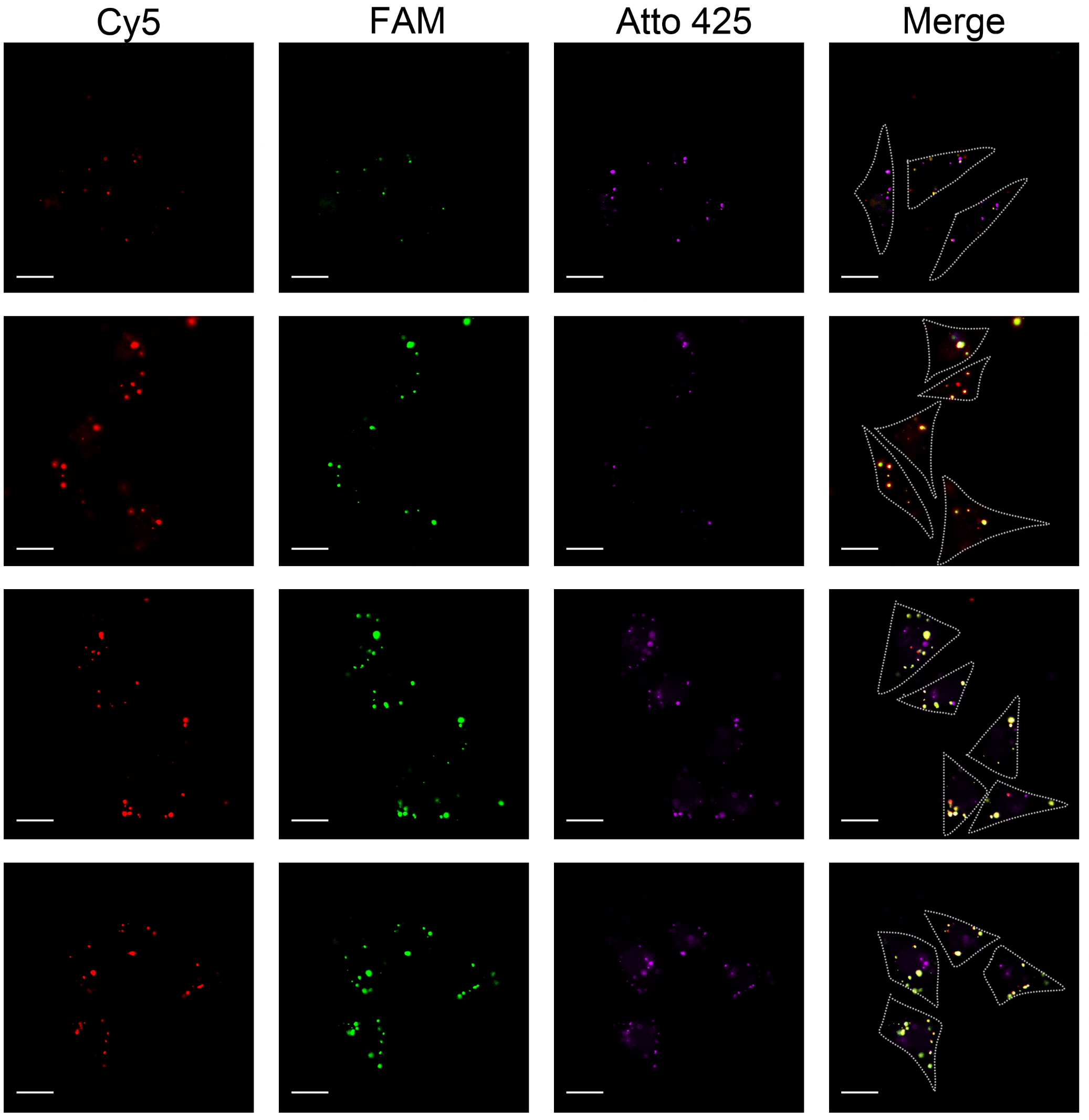


**Figure S27**. Time-dependent CLSM imaging of AS1411-blocker pre-transfected MCF-7 cells treated with the 3S-P system. (A) Images at 1 h. Scale bar: 20 μm.


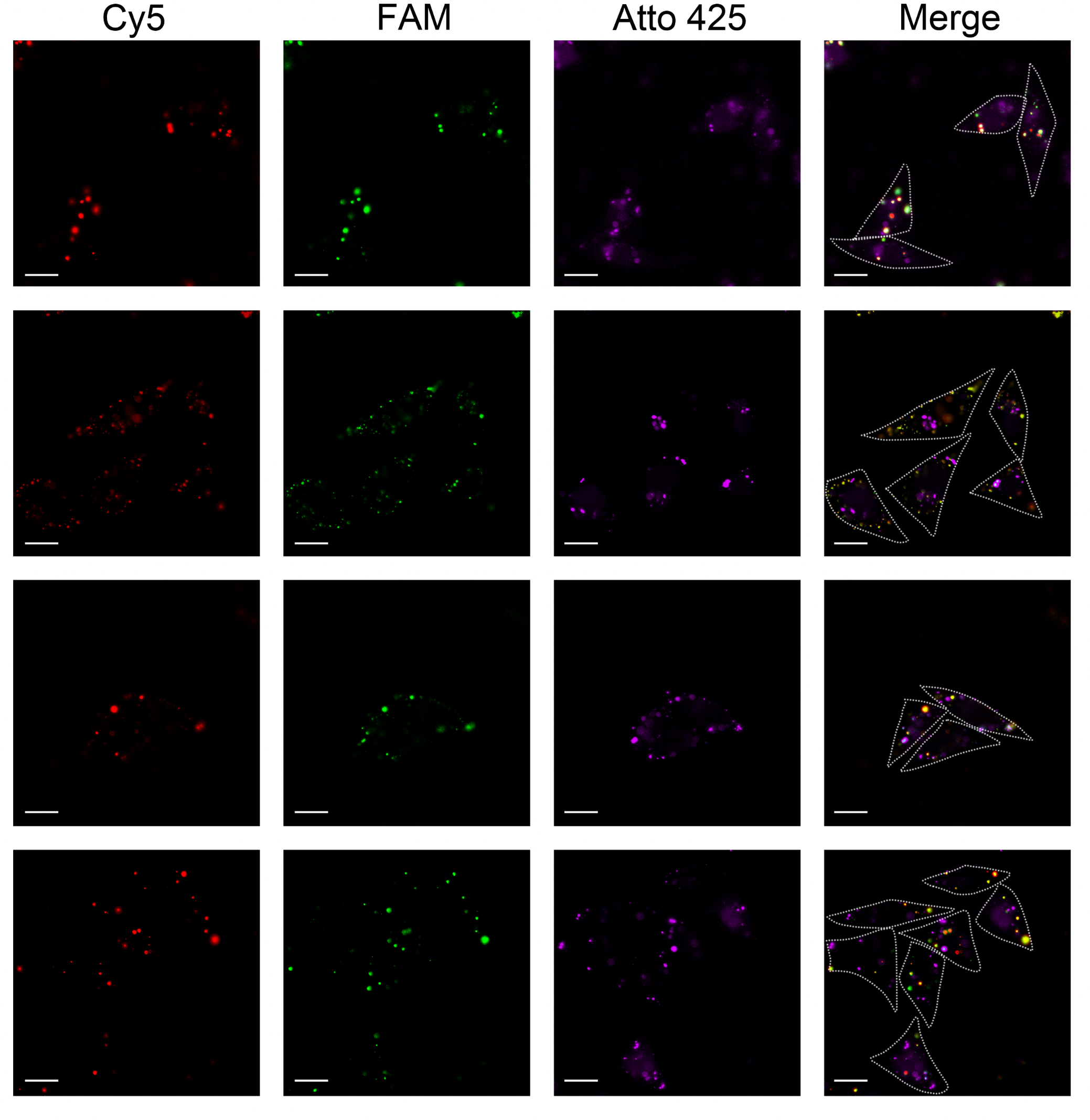


**Figure S27**. (B) Images at 2 h. Scale bar: 20 μm.


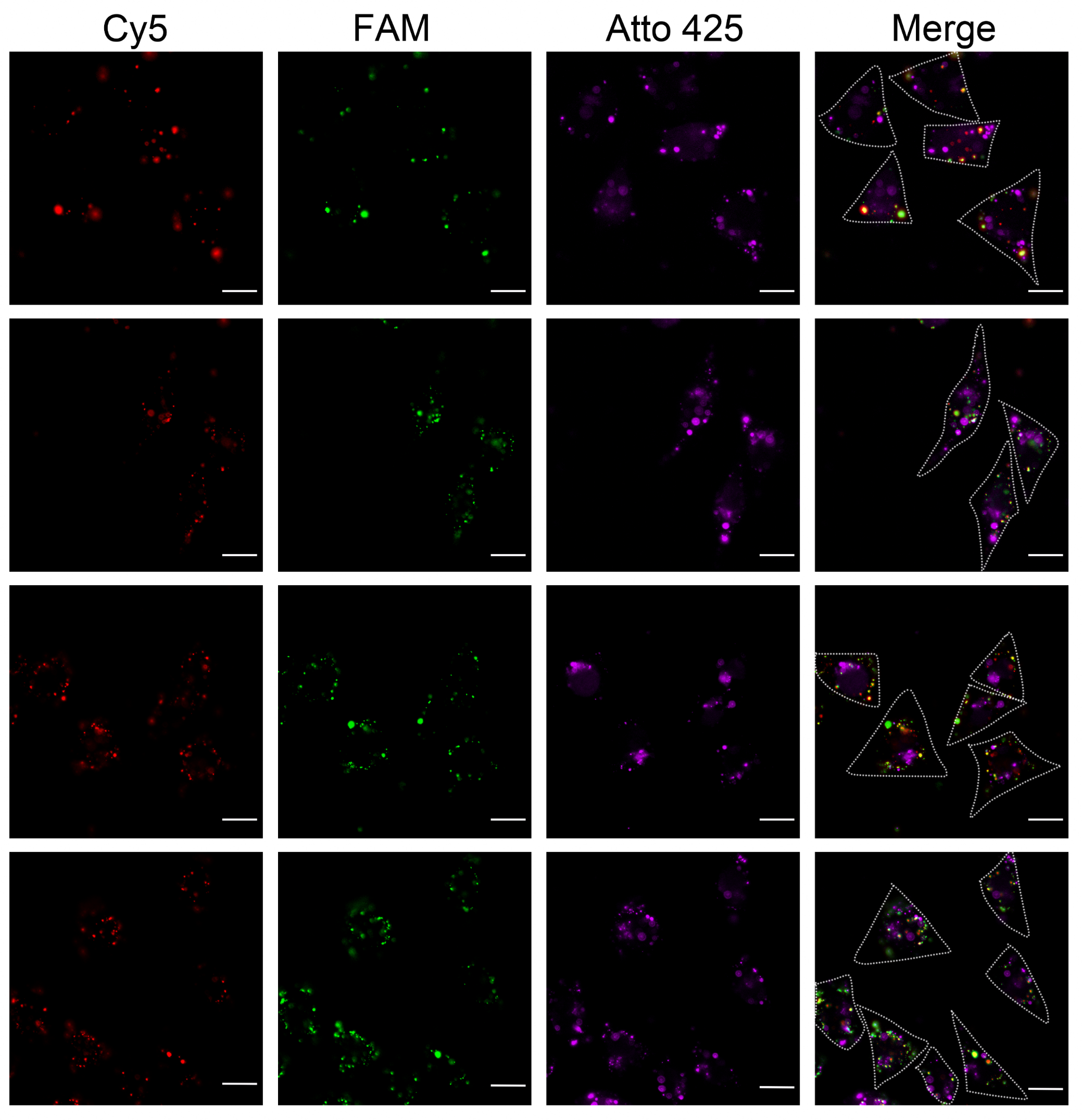


**Figure S27**. (C) Images at 3 h. Scale bar: 20 μm.


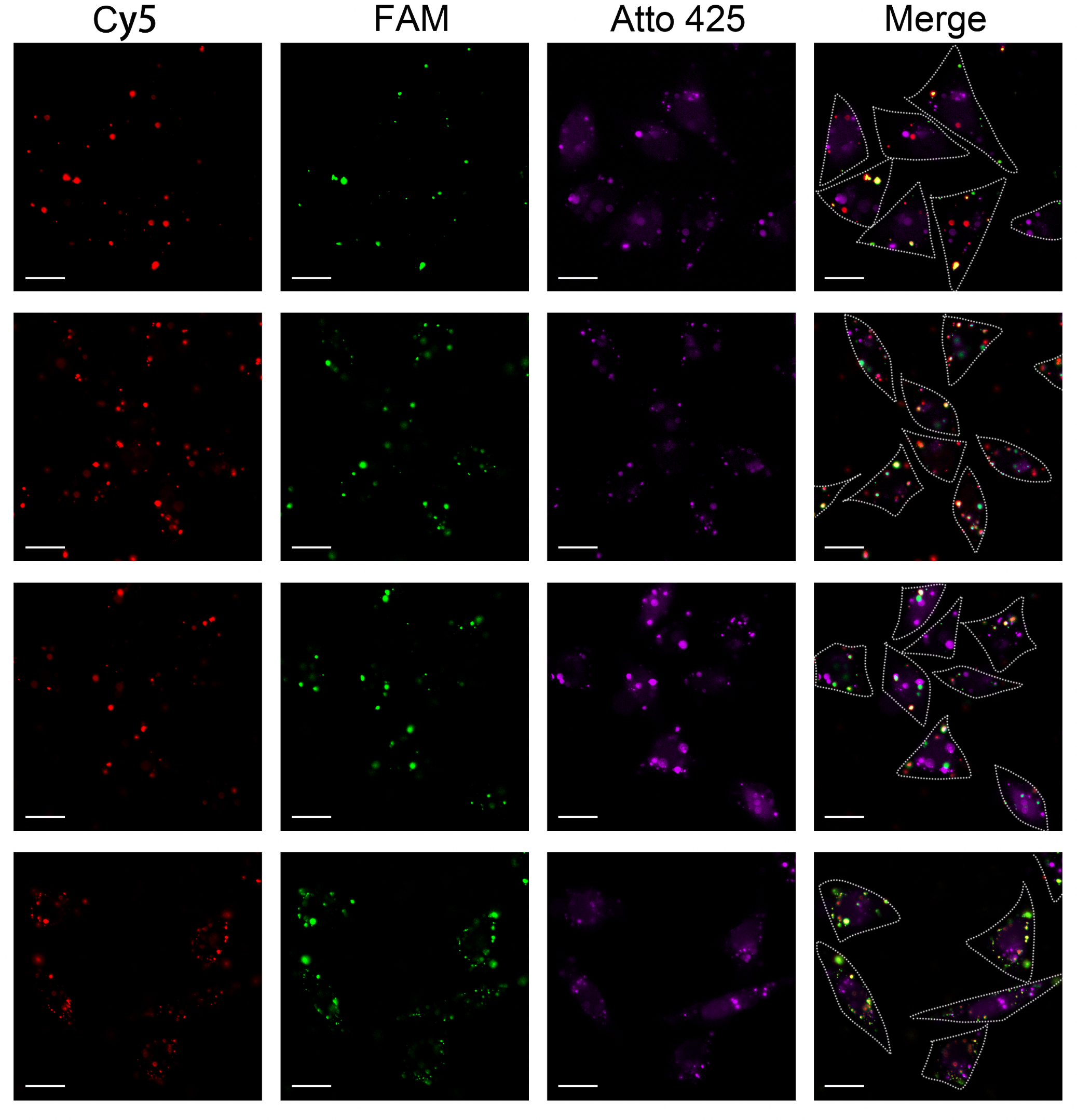


**Figure S27**. (D) Images at 4 h. Scale bar: 20 μm.


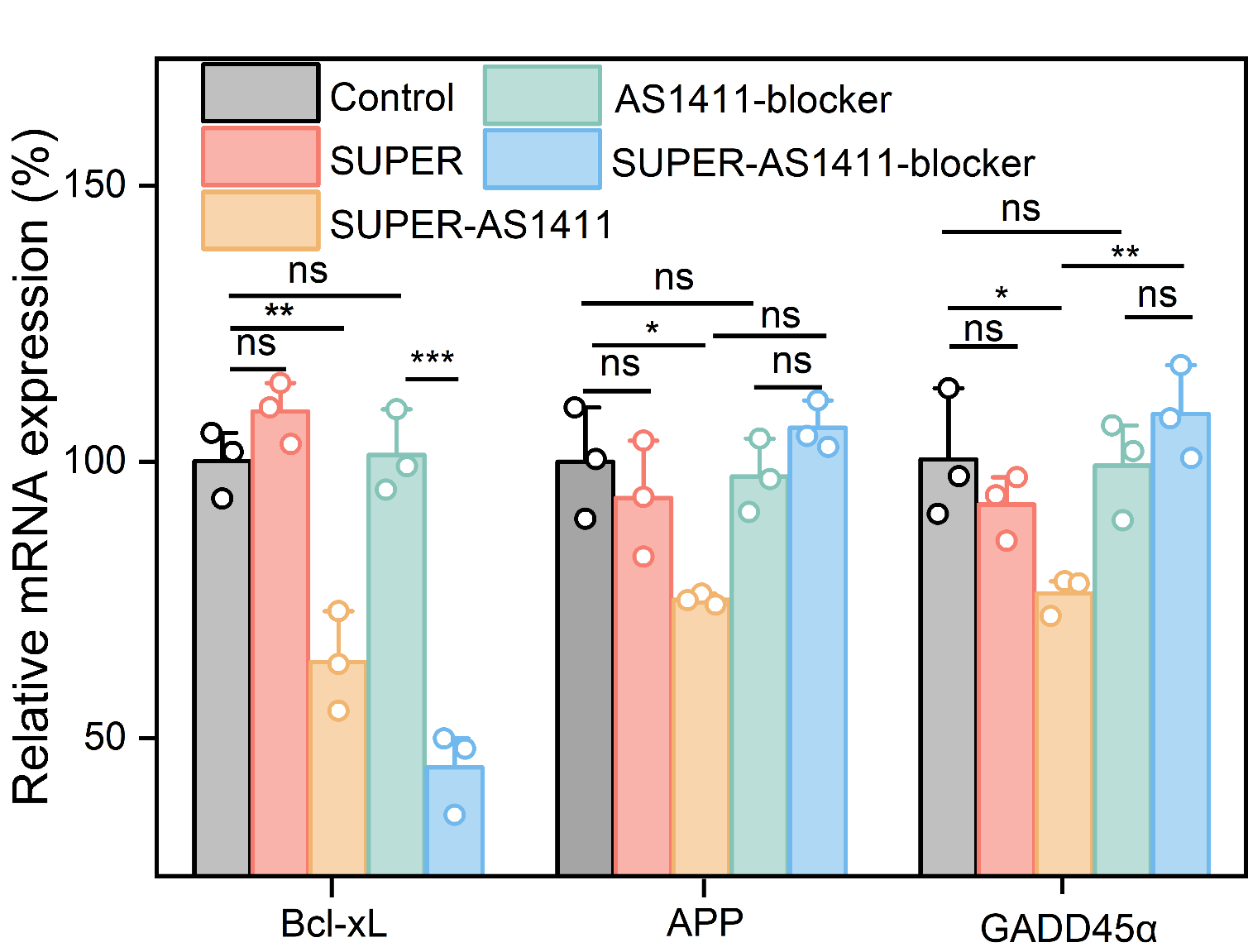


**Figure S28**. Relative mRNA expression levels of Bcl-xL, APP, and GADD45α in HeLa cells treated with different systems. Error bars represent the standard deviation (SD) from three independent experiments. p-values were determined by a two-tailed unpaired t-test. ns: no significance, *p < 0.05, **p < 0.01, ***p < 0.001, **** p < 0.0001. As shown in Figure S27, the expression profiles of Bcl-xL, APP, and GADD45α in HeLa cells exhibited trends highly consistent with those observed in MCF-7 cells. The SUPER system alone did not significantly alter the levels of any of the three transcripts relative to the control group, indicating that the presence of the split DNAzyme does not perturb endogenous mRNA stability in HeLa cells. Upon treatment with SUPER-AS1411, all three nucleolin-binding mRNAs were downregulated (~36.4% decrease in Bcl-xL, ~25.0% in APP, and ~24.3% in GADD45α), confirming AS1411-induced destabilization of nucleolin-associated transcripts in this cell line. In contrast, AS1411-blocker alone caused only marginal reductions in APP and GADD45α and had little effect on Bcl-xL, consistent with the inability of the duplexed aptamer to bind nucleolin. Importantly, the SUPER-AS1411-blocker system selectively decreased Bcl-xL expression by approximately 55.5%, while producing no significant changes in APP or GADD45α levels. This specificity demonstrates that both SUPER-mediated activation and the presence of AS1411 are required to induce targeted degradation of Bcl-xL mRNA, and that the system remains functionally precise even in a different cellular context. These HeLa data corroborate the findings in MCF-7 cells and further support the robustness and generalizability of the SUPER-based activating antenna for selective mRNA silencing.


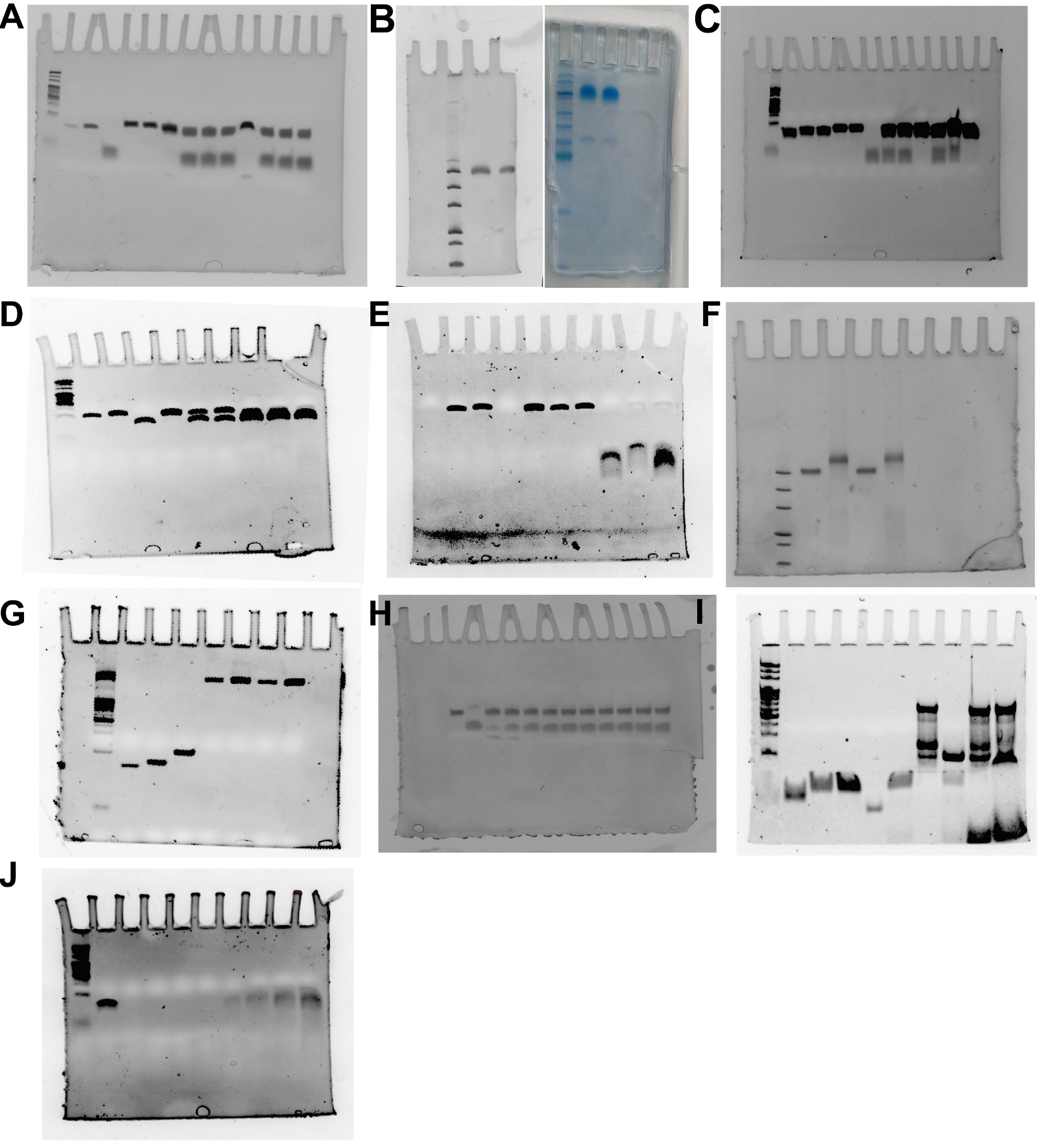


**Figure S29**. All the original gel images in this article. (A) Full-size gel for Figure 1D (top). (B) Full-size gel for Figure 1E. (C) Full-size gel for Figure S3. (D) Full-size gel for Figure S5B (top). (E) Full-size gel for Figure S5B (bottom). (F) Full-size gel for Figure S7. (G) Full-size gel for Figure S8. (H) Full-size gel for Figure S13 (top). (I) Full-size gel for Figure S25A. (J) Full-size gel for Figure S25B.

**Table S1.** The sequences of DNA oligonucleotides

| **Name** | **Sequence (5’-3’)** |
| --- | --- |
| 8-17DNAzyme | TCATTCTCCGAGCCGGTCGAAAACAGTC |
| Substrate (Sub) | Cy5-GACTGTT/rA/GGAATGA-BHQ2 |
| P_0_/P_0_ (0 bp) | TCATTCTCCGAGCCCTGTTCAAAGCTCTGAT |
| P_1_/P_1_ (0 bp) | GGAAGAGTTCATTCACTACGGTCGAAAACAGTC |
| P_2_ | AGAGTTCCACAAAAGTATCGGTCGAAAACAGTC |
| Bcl-xL | ATCAGAGCTTTGAACAGGTAGTGAATGAACTCTTCC |
| Bcl-xS | ATCAGAGCTTTGAACAGGATACTTTTGTGGAACTCT |
| P_0_ (1 bp) | TCATTCTCAGCCCTGTTCAAAGCTCTGAT |
| P_1_ (1 bp) | GGAAGAGTTCATTCACTAGCGGTCGAAAACAGTC |
| P_0_ (2 bp) | TCATTCTCCGAGCCCCTGTTCAAAGCTCTGAT |
| P_1_ (2 bp) | GGAAGAGTTCATTCACTAGGCGGTCGAAAACAG |
| P_0_ (3 bp) | TCATTCTCCGAGCGGGCCTGTTCAAAGCTCTGAT |
| P_1_ (3 bp) | GGAAGAGTTCATTCACTAGGGCGGTCGAAAACAGTC |
| Mismatch xL-1 | ATCAGAGCTTTGAACATACAGTGAATGAACTCTTCC |
| Mismatch xL-2 | ATCAGAGCTAATAACAGGTAGTGAATGAACTCTTCC |
| Mismatch xL-3 | ATCAGAGCTTTGAACAGGTAGTCTGTGAACTCTTCC |
| Mismatch xS-1 | ATCAGAGCTGACAACAGGATACTTTTGTGGAACTCT |
| Mismatch xS-2 | ATCAGAGCTTTGAACAGCGAACTTTTGTGGAACTCT |
| Mismatch xS-3 | ATCAGAGCTTTGAACAGGATACTAAGGTGGAACTCT |
| Mutant-P_0_ | TCATTCTATCAGCCCTGTTCAAAGCTCTGAT |
| Mutant-P_1_ | GGAAGAGTTCATTCACTAAATTCGAAAACAGTC |
| Mutant-P_2_ | AGAGTTCCACAAAAGTATAATTCGAAAACAGTC |
| 1S_0_-P_0_ | GACTGTT/rA/GGAATGATCATTCT  CCGAGCCCTGTTCAAAGCTCTGAT |
| 1S_0_-P_0_  (FAM-labeled) | FAM-GACTGTT/rA/GGAATGATCATTCT  CCGAGCCCTGTTCAAAGCTCTGAT |
| 1S_0_-P_0_  (Cy5/BHQ2-labeled) | BHQ2-GACTGTT/rA/GGAA/iCy5dT/GATCATTCTC  CGAGCCCTGTTCAAAGCTCTGAT |
| 1S_1_-P_1_ | GGAAGAGTTCATTCACTACGGTCGAA  AACAGTCGACTGTT/rA/GGAATGA |
| 1S_1_-P_1_  (FAM-labeled) | GGAAGAGTTCATTCACTACGGTCGAA  AACAGTCGACTGTT/rA/GGAATGA-FAM |
| 1S_1_-P_1_  (FAM/BHQ1-labeled) | GGAAGAGTTCATTCACTACGGTCGAA  AACAGTCGAC/i6FAMdT/GTT/rA/GGAATGA-BHQ1 |
| 1S_2_-P_2_ | AGAGTTCCACAAAAGTATCGGTCGAA  AACAGTC GACTGTT/rA/GGAATGA |
| 1S_2_-P_2_  (Cy3/BHQ-labeled) | AGAGTTCCACAAAAGTATCGGTCGAA  AACAGTC GAC/iCy3dT/GTT/rA/GGAATGA-BHQ2 |
| P_0_  (Biotin-labeled) | Biotin-TTTTTTTCATTCTCCGAGCCCT  GTTCAAAGCTCTGAT |
| P_1_  (Biotin-labeled) | GGAAGAGTTCATTCACTACGGTCGAA  AACAGTCTTTTTT-Biotin |
| P_2_  (Biotin-labeled) | AGAGTTCCACAAAAGTATCGGTCGAAA  ACAGTCTTTTT-Biotin |
| S_0_ | Biotin-TTTTATTTTATTTTATTTTATTTTATTT  T/iCy5dT/GACTGTTrAGGAATGA-BHQ2 |
| S_1_ | Biotin-TTTTATTTTATTTTATTTTATTTTATTT  T/i6FAMdT/GACTGTTrAGGAATGA-BHQ1 |
| S_2_ | Biotin-TTTTATTTTATTTTATTTTATTTTATTT  T/iCy3dT/GACTGTTrAGGAATGA-BHQ2 |
| Anti-P_0_ | ATCAGAGCTTTGAACAGGGCTCGGAGAATGA |
| Anti-P_1_ | GACTGTTTTCGACCGTAGTGAATGAACTCTTCC |
| AS1411 | GGTGGTGGTGGTTGTGGTGGTGGTGG |
| Blocker | CCACCACAACGACTGTT/rA/GGAATGACACCACCACC |
| AS1411  (Atto 425-labeled) | Atto 425-GGTGGTGGTGGTTGTGGTGGTGGTGG |
| Blocker  (BHQ1-labeled) | CCACCACAACGACTGTT/rA/GG  AATGACACCACCACC-BHQ1 |

rA in Substrate probe is adenine ribonucleotide.
